# Large-scale neuroanatomical study uncovers 198 gene associations in mouse brain morphogenesis

**DOI:** 10.1038/s41467-019-11431-2

**Published:** 2019-08-01

**Authors:** Stephan C. Collins, Anna Mikhaleva, Katarina Vrcelj, Valerie E. Vancollie, Christel Wagner, Nestor Demeure, Helen Whitley, Meghna Kannan, Rebecca Balz, Lauren F. E. Anthony, Andrew Edwards, Hervé Moine, Jacqueline K. White, David J. Adams, Alexandre Reymond, Christopher J. Lelliott, Caleb Webber, Binnaz Yalcin

**Affiliations:** 1 Institut de Génétique et de Biologie Moléculaire et Cellulaire, 67404 Illkirch, France; 2 Centre National de la Recherche Scientifique, UMR7104, 67404 Illkirch, France; 3 Institut National de la Santé et de la Recherche Médicale, U964, 67404 Illkirch, France; 4 Université de Strasbourg, 67404 Illkirch, France; 5 Inserm UMR1231 GAD, University of Bourgogne Franche-Comté, 21000 Dijon, France; 6 Center for Integrative Genomics, University of Lausanne, 1015 Lausanne, Switzerland; 7 Department of Physiology, Anatomy and Genetics, University of Oxford, Oxford, OX1 3PT UK; 8 Wellcome Sanger Institute, Hinxton, Cambridge, CB10 1SA UK; 9 Woodland View Hospital, NHS Ayrshire and Arran, Irvine, KA12 8SS Scotland; 10 Wellcome Centre for Human Genetics, Oxford, OX3 7BN UK; 11 UK Dementia Research Institute, University of Cardiff, Cardiff, CF24 2HQ UK

**Keywords:** Genetics of the nervous system, Developmental disorders

## Abstract

Brain morphogenesis is an important process contributing to higher-order cognition, however our knowledge about its biological basis is largely incomplete. Here we analyze 118 neuroanatomical parameters in 1,566 mutant mouse lines and identify 198 genes whose disruptions yield NeuroAnatomical Phenotypes (NAPs), mostly affecting structures implicated in brain connectivity. Groups of functionally similar NAP genes participate in pathways involving the cytoskeleton, the cell cycle and the synapse, display distinct fetal and postnatal brain expression dynamics and importantly, their disruption can yield convergent phenotypic patterns. 17% of human unique orthologues of mouse NAP genes are known loci for cognitive dysfunction. The remaining 83% constitute a vast pool of genes newly implicated in brain architecture, providing the largest study of mouse NAP genes and pathways. This offers a complementary resource to human genetic studies and predict that many more genes could be involved in mammalian brain morphogenesis.

## Abstract (web summary)

Brain morphogenesis is an important process contributing to higher-order cognition, however our knowledge about its biological basis is largely incomplete. Here, authors analyzed 118 neuroanatomical parameters in 1,566 mutant mouse lines to identify 198 genes whose disruptions yield neuroanatomical phenotypes

## Introduction

Brain morphogenesis is a complex process requiring coordinated development of multi-scale structures and relies on a system of sophisticated cues, which together contribute to the proper formation of neural circuits and higher-order cognitive functions. Genetic inheritance has a significant role in brain morphogenesis as shown in twin studies^1^. How many genes influence brain morphogenesis and what functional systems these genes operate in are important unsolved problems in developmental biology.

Several recent genome-wide association studies identified common genetic variants at five loci that together explained between 15% and 25% of the variance influencing intracranial volume^2,3^. However, malformations of brain development, affecting about 0.5% of children^4^, are due to rare mutations of large effect size that are frequent causes of intellectual disability (ID)^5,6^, autism spectrum disorders (ASDs)^7,8^, and schizophrenia^9^. These disorders originate from defects in neurogenesis, cell survival, neuronal migration, axon guidance, and synaptogenesis, and while our capacity to detect genetic variants has significantly improved^10^, distinguishing pathogenic mutations from normal variation remains an issue^11^. Their disabling nature together with the fact that as many as 60% of cases are of unknown genetic etiology^6^ also calls for ways to improve clinical identification and functional stratification, as both are key for better treatment.

Given that combined genetic and large-scale anatomical analysis of the human brain is technically challenging at high resolution, we turned to the mouse where both the environment and genetic background are controlled and where cell-level resolution is achievable using histology. Although the complex cortical folding of the human brain is absent in the naturally lissencephalic rodent brain, humans and rodents share neurodevelopmental principles. This view is supported by the identification of mutations in mice even before their implication in human cerebral developmental disorders, such as *TUBA1A*^12^ and *EML1*^13^, demonstrating that mutations in the mouse translate to the clinic^14^. Here we utilize a reverse-genetic screen in mice and perform a systematic and accurate assessment of neuroanatomical defects in 1566 genetic mutant lines in order to identify key genes and functional pathways modulating brain morphogenesis. We then show how this resource can help gain insights into the biological mechanisms leading to neurodevelopmental and psychiatric disorders.

## Results

### Gene identification for NeuroAnatomical Phenotypes (NAPs)

Through collaboration with the Sanger Institute Mouse Genetics Project, a partner of the International Mouse Phenotyping Consortium (IMPC), we obtained brain samples from 4796 adult male mice for heterozygous and/or homozygous or hemizygous mutations (Supplementary Notes), as well as 1418 matched wild types (WTs). In all, 88% of mouse mutants were generated using the Knockout-first allele method^15^ (Supplementary Data 1; Supplementary Notes). To maximize the number of alleles tested, only males were analyzed in this study despite evidence of sexual dimorphism in mammalian brain-related traits^16^.

This dataset represents 1566 alleles, each studied with three biological replicates, from 1446 unique genes (Supplementary Notes), of which 1394 are protein coding, 32 single or multiple RNA genes, 10 CpG islands, 4 unclassified, 3 chromosomal deletions, 2 pseudogenes, and 1 gene segment (Supplementary Data 1). Multiple constructions were analyzed for 105 of these genes including allelic variants at 43 loci, 29 studied both at heterozygous and homozygous state, 19 at different age points (mainly 6 and 16 weeks of age), and the remaining 29 were a mixture of these or additional controls (Supplementary Data 2; Supplementary Notes). Overall, 80% of alleles were maintained on a pure inbred C57BL/6N background, 18% on mixed C57BL/6 backgrounds, and 2% with other genetic backgrounds (129, CBA and C3Fe), the latter being always analyzed alongside matched controls since the neuroanatomy of these strains of mice can significantly differ from one another (Supplementary Notes).

**Fig. 1:**
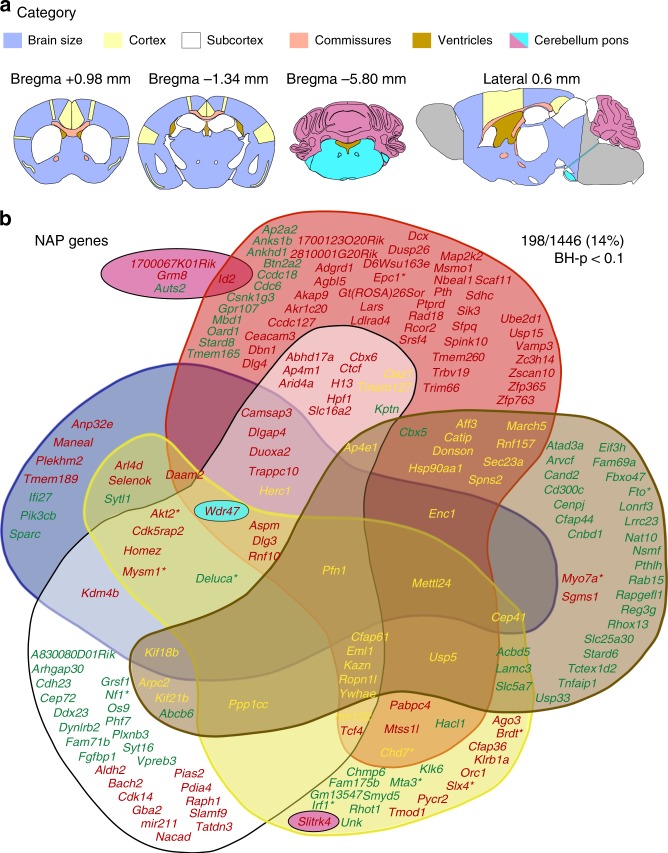
Gene identification for neuroanatomical phenotypes. **a** One hundred and eighteen brain parameters (Supplementary Data 4; Supplementary Fig. 2) are grouped into six categories (brain size, cortex, subcortex, commissures, ventricles and cerebellum/pons) on coronal and sagittal sections at the indicated positions. **b** NeuroAnatomical Phenotype (NAP) genes (mouse genes whose disruptions yield a neuroanatomical defect) are positioned on each category and color-coded. Red font corresponds to decrease in structure size, green to increase, yellow to both, and asterisks refer to the 6-week dataset (Supplementary Notes). BH-*p* < 0.1 corresponds to the adjusted Benjamini–Hochberg *p* value using a linear mixed model

Neuroanatomical data were collected down to cell-level resolution blind to the genotype using a histological pipeline (Supplementary Fig. 1a; Supplementary Notes) on symmetrical and stereotaxic planes of the adult mouse brain at Bregma +0.98 mm, −1.34 mm, and −5.80 mm as well as at Lateral 0.60 mm (named critical sections)^17^. The sagittal plane offers benefits we discussed elsewhere^18^, while essentially covering and enabling us to detect NAP variation in the same structures present on coronal sections^19^. Eighty-five co-variates (Supplementary Data 3) and 118 brain morphological parameters mainly consisting of area (63%) and length (36%) measurements of anatomical structures (Supplementary Fig. 2; Supplementary Data 4) were quantified (Supplementary Data 5) and stored into a relational database (Supplementary Fig. 3; Supplementary Notes). These parameters encompass six main categories: brain size, commissures (callosal, anterior, hippocampal, fornix, and stria medullaris), ventricles (lateral, third, and fourth), cortex (motor, somatosensory, cingulate, piriform, retrosplenial, and temporal), subcortex (hippocampus, amygdala, tracts, caudate putamen, internal capsule, habenula, fimbria, thalamus, hypothalamus, substantia nigra, subiculum, and colliculus), and cerebellum (granule layer, cerebellar nuclei, number of folia, pons, nerves, and pontine nuclei) (Fig. 1a; Supplementary Data 4). A two-dimensional (2D) map of brain parameters from WT animals plotted using a dimensionality reduction method (t-Distributed Stochastic Neighbor Embedding (t-SNE)) revealed the expected clusters of related anatomical features within and across critical sections (Supplementary Fig. 4a) arguing that data structure was preserved and sound. Brain structural correlation analysis is also provided in Supplementary Fig. 4b–d on raw and normalized data (to the total brain area) sets (see Supplementary Notes for more details).

A standardized statistical pipeline for the detection of neuroanatomical phenotypes was developed in R using PhenStat, a package providing a variety of statistical methods for the analysis of large-scale phenotypic associations from the IMPC^20^ (Supplementary Fig. 5; Supplementary Notes). Using PhenStat’s linear mixed model (LMM) framework (https://bioconductor.org/packages/release/bioc/vignettes/PhenStat/inst/doc/PhenStatUsersGuide.pdf), the necropsy date of the animal was computed as a random effect variable in order to account for temporal variation that may have occurred since the start of the study in 2009. This achieved the best fitting model (highest Maximum Likelihood Estimate score) against other models taking into account co-variates such as body or brain weights and total brain area (Supplementary Fig. 5; Supplementary Notes). Alleles maintained on mixed inbred strain backgrounds (2% of the data) were analyzed independently using Student’s *t* test (Supplementary Data 6). The study’s statistics are detailed in Supplementary Notes.

We identified 198 genes associated with neuroanatomical defects (Fig. 1b; Supplementary Data 7) at the adjusted Benjamini–Hochberg *p* value (BH-p) of <0.1, hereafter named as NAP genes. Brain abnormalities were annotated using existing or newly created Mammalian Phenotype Ontology (MPO) terms (Supplementary Data 8). A heat map of all tested genes is provided in Supplementary Data 9. Percentage change, *z*-score, and unadjusted and adjusted *p* values are listed in Supplementary Data 10. Commissures were most affected (32%), followed by subcortical malformations (21%), ventricles (18%; frequently enlarged), cortical anomalies (17%), brain size defects (10%), and cerebellar/pons anomalies (2%) (Fig. 1b).

**Fig. 2:**
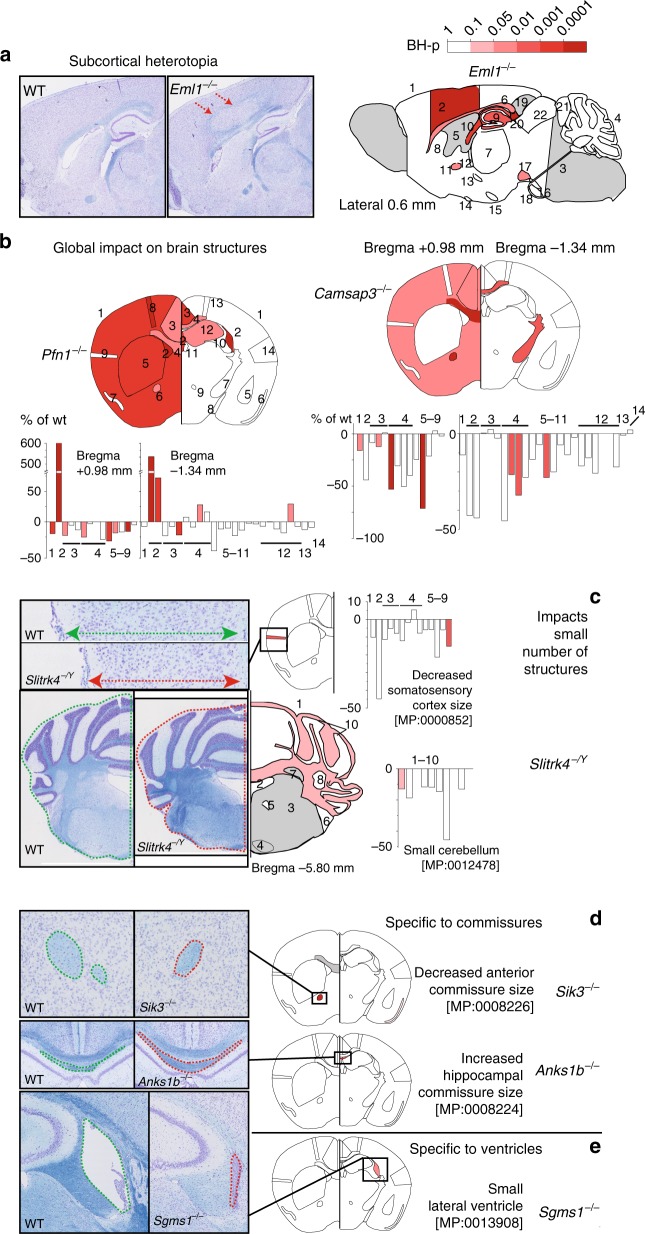
Impact of mutations on the brain architecture. **a** Example of cortical heterotopia in *Eml1*^*−/−*^ (left) (image width 0.6 cm) and other affected brain regions mapped on a schematic representation of the sagittal plane at Lateral 0.6 mm. **b** Mutations having a global impact on the brain architecture (*Pfn1*^*+/−*^ and *Camsap3*^*−/−*^) with brain parameters mapped on a schematic representation of the two coronal sections at Bregma +0.98 mm and Bregma −1.34 mm. Bar graphs detail which regions are affected using a color code corresponding to the adjusted Benjamini–Hochberg *p* value (BH-p). **c** Example of a mutation affecting a small number of parameters: the somatosensory cortex at Bregma +0.98 mm (red arrow 0.09 cm) and the cerebellum at Bregma −5.80 mm (*Slitrk4*^*−/Y*^) (image width 0.3 cm). **d**, **e** Examples of mutations having a specific impact on: **d** the commissures (*Sik3*^*−/−*^ and *Ank1b*^*−/−*^) (image width 0.06 cm for *Sik3*^*−/−*^ and *Ank1b*^*−/−*^) and **e** the ventricles (*Sgms1*^*−/−*^) (image width 0.1 cm for *Sgms1*^*−/−*^). Details of measurements appear in Supplementary Fig. 2. MP refers to the Mammalian Phenotype terms used to annotate the brain abnormalities (Supplementary Data 8)

The most severe cortical malformation detected is an occurrence (*Eml1*^*−/−*^) of subcortical heterotopia with thinner homotopic cortex and abnormal fiber tracts surrounding it (Fig. 2a) reminiscent of a previous *Eml1*-spontaneous mouse mutant^21^, but our screen identified additional defects: corpus callosum dysgenesis, abnormal hippocampus, and enlarged fimbria. *Eml1* is one of the 51% of NAP genes that impacted on two or more brain categories. Examples of global impact on brain architecture include *Camsap3*^*−/−*^ and *Pfn1*^*+/−*^ (Fig. 2b). *Camsap3*^*−/−*^ is a representative example of microcephaly where the total brain area, the cingulate cortex, the genu of the corpus callosum, the anterior commissure, the soma of the corpus callosum, and the internal capsule were all reduced in size, ranging from −12% to −71% (for details, see Supplementary Data 10). *Pfn1*^*+/−*^ is an illustrative example of extreme but complex hydrocephaly where other regions are making up for lost space: the total brain area, the cingulate cortex, the genu of the corpus callosum, the caudate putamen, the anterior commissure, the motor cortex, and the retrosplenial cortex were all reduced in size, ranging from −16% to −32% (BH-p < 0.1; LMM), while the ventricles and the height of the corpus callosum were increased ranging from +28% to +633% (BH-p < 0.02; LMM). Other examples of models with global impacts include *Akap9*^*+/−*^, *Cbx6*^*−/−*^, *Daam2*^*−/−*^, *Mysm1*^*+/−*^, *Rnf10*^*−/−*^, *Ropn1l*^*−/−*^, and *Trappc10*^*−/−*^ (Supplementary Data 9).

On the other hand, *Abcb6*^*−/−*^, *Slitrk4*^*−/Y*^, and *Sytl1*^*−/−*^ had an impact on the size of two brain regions (see Fig. 2c for *Slitrk4*^*−/Y*^: −15.2%, BH-p = 0.009 for the somatosensory cortex and −12.9%, BH-p = 0.07 for the cerebellum; LMM). The remaining 49% caused specific abnormalities pertaining to a single category, of which 80% affected a single parameter. For example, *Sik3*^*−/−*^ altered the anterior commissure (−39.5%, BH-p = 1.98E-08; LMM), *Anks1b*^*−/−*^ the dorsal hippocampal commissure (+91.4%, BH-p = 1.24E-06; LMM) (Fig. 2d), and *Sgms1*^*−/−*^ the size of the ventricles (−167%, BH-p = 0.01; LMM) (Fig. 2e). In all, 46% of NAP genes decreased the size of the affected structures, while 38% increased their sizes (mainly ventricles) and only 16% had bidirectional effects (Fig. 1b). We defined four severity groups based on the percentage change relative to WTs (Supplementary Fig. 1b): mild (0–10%), moderate (10–20%), severe (20–40%), and very severe (>40%), the latter two groups being the most common with the maximum being 727% for the ventricles in *Cep41*^*−/−*^.

The vast majority of NAP genes (94%) have never been previously assessed and associated with brain anatomy in mice, for example, loss of *Smyd5*, a lysine methyltransferase gene, increases the retrosplenial granular cortex by 17.5% (BH-p = 0.01; LMM). The remaining reproduced previous mouse reports (Supplementary Data 1), for example, inactivation of *Dlg3*, a postsynaptic gene associating with GluN2 subunits of *N*-methyl-d-aspartate (NMDA) receptors, yield a smaller cortex and white matter structures as shown previously^22^ but also decreases hippocampal size (−25.4%, BH-p = 0.08; LMM).

To assess experimental sensitivity, we compared the 1248 non-NAP genes and their phenotypic associations against existing MPO annotations from the Mouse Genome Informatics (MGI) database^23^ and identified only one gene reported previously (*Trappc9*) for which we did, however, detect a low-confidence neuroanatomical change. We demonstrated experimental reproducibility using technical repeats of 120 mutant lines engineered twice using various constructions, which showed consistent results, with the exception of *Fundc1* (Supplementary Data 2).

### Relevance to human neurodevelopmental diseases

As the following analyses required full datasets, a Bayesian mixed model approach^24^ was used to impute missing data (Supplementary Data 11) in a subset comprising 1380 alleles representing samples with C57BL/6 background at similar age (Supplementary Data 12). The same statistical framework than for non-imputed datasets was used (Supplementary Data 13) yielding a consistent set of 196 NAP genes at the adjusted *p* value (BH-p) of <0.1 (Supplementary Data 14; Supplementary Fig. 6; Supplementary Notes). Unique human (1:1) orthologs were identified for 173 of these 196 mouse NAP genes.

**Fig. 3:**
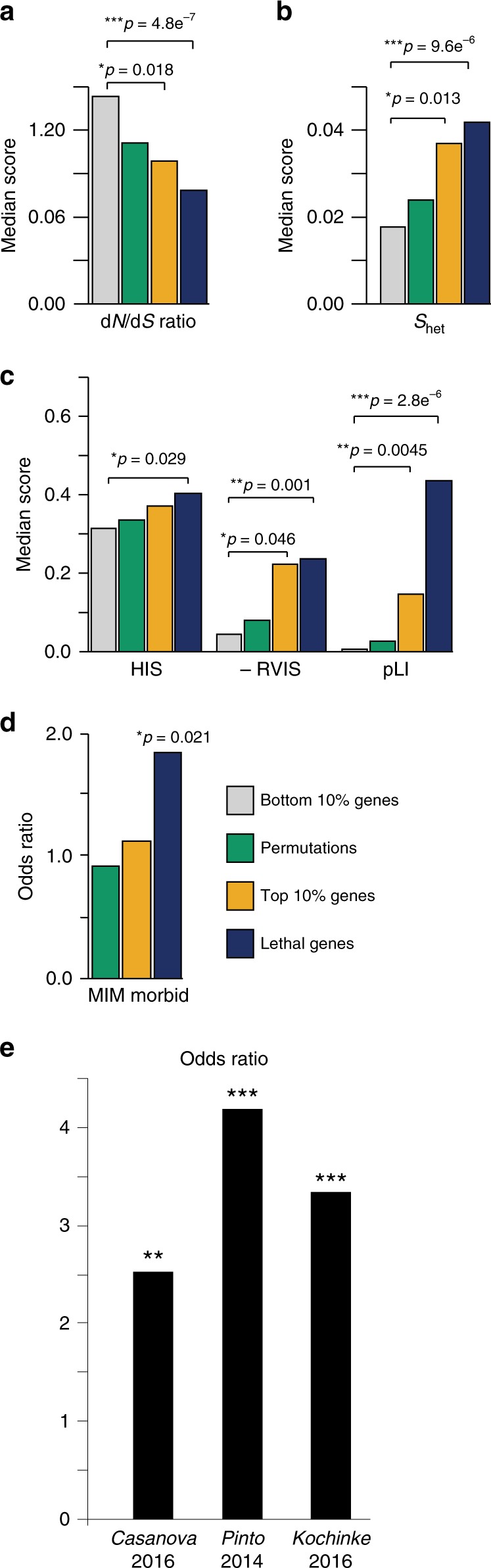
Predicted gene deleteriousness in humans. **a** Mouse lethal genes^51^ and the top 10% of mutants with the largest neuroanatomical abnormalities are compared to the bottom 10% and to randomly permuted sets of these genes. The dN/dS ratio examines selection pressures by comparing the rate of synonymous and non-synonymous substitutions. **b**, **c** Comparison of the gene properties of the human orthologs of lethal genes^51^ and of the top 10% of mutants with the largest neuroanatomical abnormalities as compared to genes in the bottom 10% and to randomly permuted sets of these genes. *s*_het_ (Selection coefficient associated with the loss of heterozygosity), RVIS (Residual Variation Intolerance Score), and pLI (probability of being Loss of function Intolerant) are all indicators of purifying selection pressure, while HIS are predicted HaploInsufficiency Scores (Supplementary Notes). **d** The overlap between gene sets from A and B with MIM Morbid Map genes. **e** Enrichments of intellectual disability-associated genes from three independent publications and developmental disorder-associated genes (Supplementary Notes) among human orthologs of NeuroAnatomical Phenotype (NAP) genes compared to human orthologs of non-NAP. *0.05 < *p* < 0.01; **0.01 < *p* < 0.001; ***p < 0.001 (right-tailed Mann–Whitney *U* test for **a**–**d** and right-tailed Fisher’s test for **e**)

To examine the relevance of genes associated with mouse neuroanatomical phenotypes to humans, we first compared the features of human unique orthologs of genes whose disruption in mouse models yielded the largest neuroanatomical variation (top 10%) to those study genes showing the least (bottom 10%) and evaluated differences to randomly permutated gene sets (Supplementary Notes). The human orthologs of top 10% genes (i) undergo strong negative selection (dN/dS), (ii) have relatively high selection coefficients (*s*_het_), (iii) are more likely to be haploinsufficient (HIS), (iv) are depleted of genetic variation (RVIS (Residual Variation Intolerance Score)), and (v) are predicted to be intolerant to protein-truncating mutations (pLI (probability of being Loss of function Intolerant)) (Fig. 3a–c). More generally, we found a significant correlation between three of these measures and the strength of brain abnormalities (defined as the maximum absolute *z*-score deviation from WT mice) induced by disruptions of the associated mouse orthologs (*p* = 0.050, *p* = 0.038, and *p* = 7.7E10−5 for *s*_het_, RVIS, and pLI, respectively; linear regression). While not globally enriched in known human disease genes (Fig. 3d), we found an excess of ID-associated genes from three independently curated gene lists^25–27^ (*p* < 0.005 for all sets; right-tailed Fisher test) (Fig. 3e). Although no excess of autism genes was found (Supplementary Fig. 6), we observed an overrepresentation of a subset of ID-associated genes comorbid with ASD (*p* = 0.0015 and odds ratio = 8.50; right-tailed Fisher test)^25^.

Next, using the manually curated catalog of 1108 primary human ID-associated genes (SysID database; https://sysid.cmbi.umcn.nl/; June 2018 download), 56% (627) of which account for ID without brain malformations and 43% (481) for ID co-occurring with brain malformations^26^, we identified an overlap of 30 NAP genes (Supplementary Data 15) of human unique orthologs of genes whose disruption in mouse models yielded neuroanatomical phenotypes (BH-p < 0.1; LMM). Nineteen of the 30 genes (*AP4E1*, *AP4M1*, *ASPM*, *AUTS2*, *CDK5RAP2*, *CENPJ*, *CEP41*, *CTCF*, *DCX*, *DONSON*, *EHMT1*, *HERC1*, *KPTN*, *LAMC3*, *MCPH1*, *ORC1*, *SLC16A2*, *TCF4*, and *TMEM165*) are linked to highly or fully penetrant brain structural abnormalities (mainly microcephaly/macrocephaly) in many patients and for which 4 genes (*ASPM*, *CENPJ*, *DCX*, and *LAMC3*) had been previously reported using mouse models, while the remaining 15 are new in vivo mouse models exhibiting equivalent neuroanatomical defects to humans (with the exception of *DONSON*). The identification of finer-scale structural anomalies pertaining to the commissures in these models further supports brain connectivity perturbation as a common disease mechanism for ID. Eleven of the 30 genes (*ADAR*, *ANO10*, *C12orf4*, *CEP120*, *DLG3*, *DLG4*, *GBA2*, *GTPBP3*, *PSPH*, *SLC5A7*, and *ZC3H14*) are primary ID genes annotated as not known to be associated with structural brain malformations, although when we used a different source (OMIM or Pubmed), low-penetrance structural brain anomalies were, however, reported for some of these genes (Supplementary Data 15).

Of the remaining 143 (83%) human unique orthologs of mouse NAP genes that did not overlap with primary ID-associated disease genes, our data may help in improving clinical interpretation especially if the identification of additional cases proves difficult or when the pathogenicity of variants is uncertain. For example, a novo missense mutation in *CAMSAP3*^28^, a gene involved in the regulation of microtubules dynamics, segregates in one patient with a nervous system phenotype in the Deciphering Developmental Disorders study^29^. *Camsap3* mutation in mice yielded major neuroanatomical defects, including microcephaly and thin corpus callosum. Our data can also help in dissecting copy number variation regions associated with brain defects in neurodevelopmental cohorts (Supplementary Data 16). For example, *ARVCF*, localized to the 22q11.21 region has been linked to schizophrenia^30^. Our study associates disruption of *Arvcf* with abnormalities in the striatum (smaller), while in the Simons Simplex Collection^31^ we identified a de novo nonsense mutation affecting *ARVCF* in an ASD patient presenting with microcephaly. These observations suggest that the less-studied gene *ARVCF* may have a prominent etiological role in the 22q11.21 neurodevelopmental disease-associated phenotypes.

### Functional characterization of NAP genes

Genomic data sources including gene expression, protein–protein interactions (PPIs) and functional mouse and human datasets were used to identify functional convergence among mouse NAP genes derived from our largest and most powerful dataset on coronal sections (Supplementary Notes). Randomly permutated gene sets drawn from the genomes were used as controls.

**Fig. 4:**
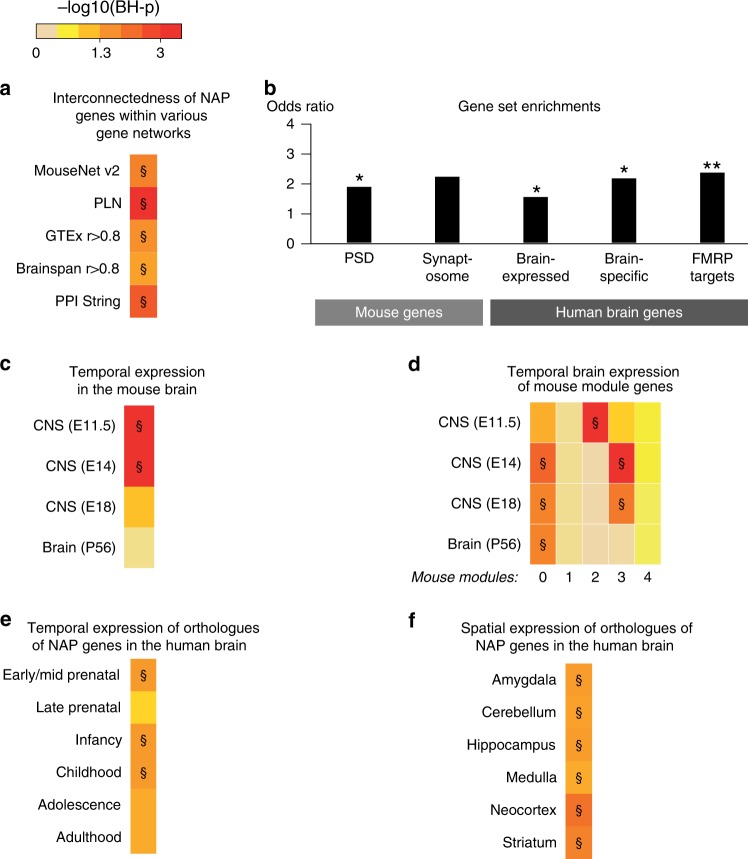
Functional annotation of mouse NeuroAnatomical Phenotype (NAP) genes and their unique 1:1 human orthologs. All heat maps use the adjusted *p* value color key shown at the top of the figure with double S (§) referring to BH-p < 0.05 (permutation test). **a** Interconnectedness of mouse NAP genes and their human orthologs within various gene networks as compared to randomly sampled genes, matched for coding DNA sequence (CDS) and network connectivity. PLN Phenotypic Linkage Network, expression data GTEx, BrainSpan, PPI protein–protein interaction. MouseNet v2 uses mouse genes, while the remaining networks use unique human 1:1 orthologs. **b** Brain, synaptic, and Fragile X Mental Retardation Protein target gene enrichments observed among mouse NAP genes and their human orthologs compared to randomly sampled genes. Gene sets are described in Supplementary Notes. *0.05 < *p* < 0.01; **0.01 < *p* < 0.001 (right-tailed Fisher’s test). **c** NAP genes’ expression in the mouse developing and postnatal central nervous system (CNS) and **d** their sub-clustering into groups of genes (modules) with distinct spatiotemporal expression dynamics in the mouse embryonic CNS or adult brain, compared to 1000 permuted gene sets drawn from the genome and matched for CDS to NAP genes. **e** Human NAP orthologs’ expression in the brain at six human developmental stages and **f** in six human brain regions (BrainSpan). Results are compared to randomly sampled genes matched for CDS to NAP orthologs (**e**, **f**)

Our 158 mouse coronal NAP genes showed a strong interconnectedness within a large-scale functional gene network (Fig. 4a; MouseNet) supporting overall functional similarities and participation in shared molecular pathways. Gene ontology (GO) enrichment analysis revealed that mouse NAP genes are localized to the cytoskeleton and microtubules (Supplementary Fig. 7). Roles in synaptic transmission were supported by an excess of postsynaptic density (PSD) and Fragile X Mental Retardation Protein (FMRP) target genes (*p* = 0.017 and *p* = 0.0035, respectively; Fig. 4b; right-tailed Fisher test). Mouse NAP genes are strongly expressed in the developing central nervous system (CNS), and in particular, upregulated during early/mid (E11.5–E14) prenatal development (Fig. 4c). Within the co-expression network (Supplementary Fig. 8), the genes associate with five separate clusters (named mouse modules) with distinct fetal- and adult-expressed brain temporal expression dynamics (Fig. 4d).

Unique human (1:1) orthologs were identified for 147 of the 158 mouse NAP genes (Supplementary Data 1; Supplementary Notes) and displayed similar functional annotations to mouse genes (Supplementary Fig. 7). They are expressed in the human brain (Fig. 4b), and in particular, upregulated not only during early/mid prenatal development (Fig. 4e), consistent with the mouse dataset, but also throughout infancy and childhood (Fig. 4e) but do not show region-specific expression (Fig. 4f). Human orthologs of mouse NAP genes cluster strongly within a Phenotypic Linkage Network (PLN; randomisations *p* < 0.001; Fig. 4a), an integrated gene network constructed by combining several human genomic data sources^32^. These results are replicated using a PPI network and high-coverage unbiased body-wide and brain developmental gene co-expression networks (Fig. 4a; Supplementary Data 17).

**Fig. 5:**
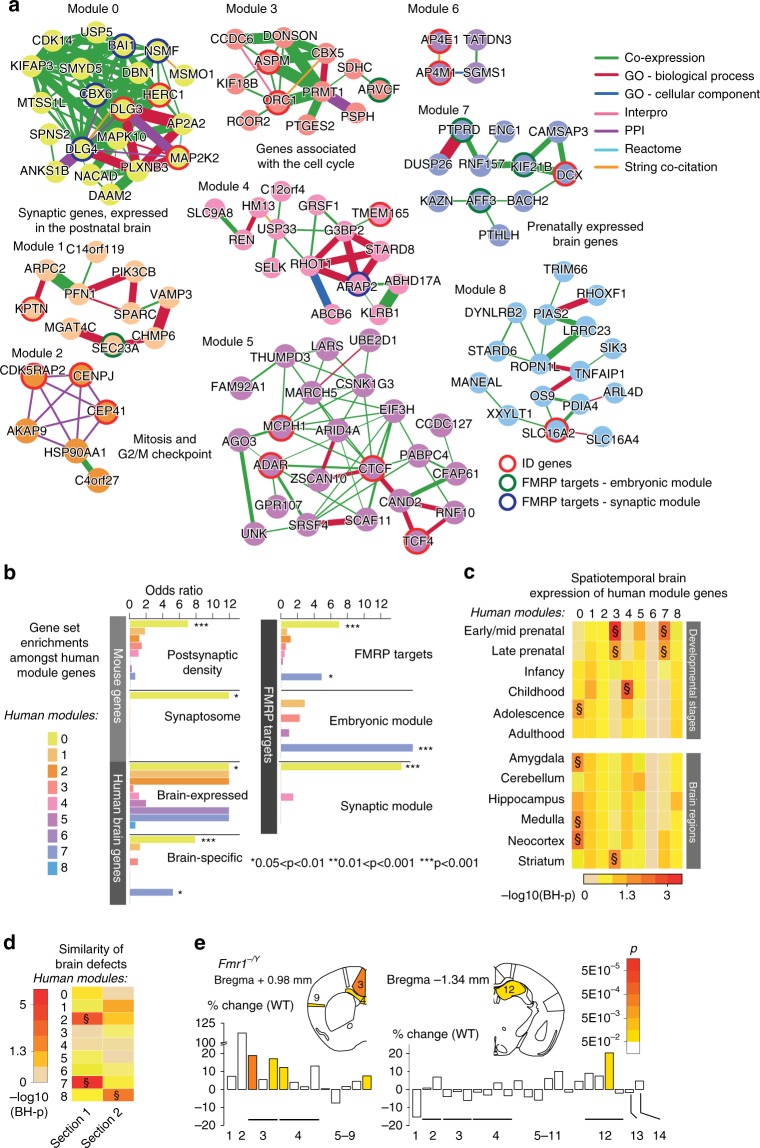
Functional characterization of human gene modules. **a** The PLN (Phenotypic Linkage Network) identifies 381 functional links between 121 human orthologs of NeuroAnatomical Phenotype (NAP) genes, which partitioned into the 9 modules of closely related genes (illustrated by the color of the nodes). The thickness of each edge is proportional to the functional similarity score, as given by the PLN, and the color of each edge indicates the largest contributing information source. Red borders depict known ID-associated genes, whereas blue and green borders refer to the set of embryonically expressed and mature Fragile X Mental Retardation Protein (FMRP) target genes, respectively. Module descriptions were added, where clearly discernable. **b** Gene set enrichment analysis across the nine human modules. **c** Spatiotemporal expression dynamics of module genes compared to the remaining NAP orthologs. **d** The heat map depicts the similarity of brain abnormalities caused by module genes in the critical sections 1 and 2 (Bregma +0.98 mm and −1.34 mm, respectively). The color code corresponds to the adjusted *p* value with double S (§) referring to BH-p < 0.05 (**c**, **d**; permutation test). **e** New neuroanatomical study of *Fmr1*^*−/Y*^ in the coronal plane (*n* = 4 wild types (WTs) and *n* = 6 *Fmr1*^*−/Y*^, male). Top: Schematic representation of the affected brain parameters at Bregma +0.98 mm and −1.34 mm. Numbers refer to the same brain parameters from Fig. 2b and Supplementary Fig. 2b, c. The color code indicates the unadjusted *p* value. Bottom: Histograms showing the percentage of increase/decrease of the brain parameters compared to WTs

Sub-clustering of the 147 unique human orthologs within the PLN network reveals nine functionally distinct gene modules (Fig. 5a). Similar mouse modules were uncovered when grouping NAP genes based on gene expression dynamics across the developing mouse CNS (Supplementary Fig. 8), reinforcing the relevance of translational studies between human and mouse. The human module 2 was enriched for cell cycle-related GO annotations specifically the G2/M checkpoint (Fig. 5a; Supplementary Data 18), implicating these genes in early neurodevelopmental processes. By contrast, human modules 0 and 7 exhibit an excess of brain-specific (*p* = 0.002 and *p* = 0.045, respectively; right-tailed Fisher’s test) and human orthologs of FMRP target genes (*p* = 7.81E−04 and *p* = 0.030, respectively; Fig. 5b; right-tailed Fisher test). Moreover, module 0 is also enriched for human orthologs of mouse PSD and synaptosome genes (*p* = 7.81E−04 and *p* = 0.019, respectively; Fig. 5b; right-tailed Fisher’s test) and the associated genes are functionally annotated with the GO terms PSD (BH-p = 0.0015; right-tailed hypergeometric test), cell projection (BH-p = 0.040; right-tailed hypergeometric test), and cytoskeleton (BH-p = 0.026; right-tailed hypergeometric test) (Supplementary Data 18). While both modules possess neuronal functions, genes from module 0 are upregulated postnatally, while those in module 7 are upregulated in the fetal brain (Fig. 5c), suggesting that each module contributes to distinct neurodevelopmental and mature synaptic functions. Accordingly, modules 7 and 0 are enriched in different subsets of fetally and postnatally expressed FMRP target genes (*p* = 0.0038 and *p* = 0.0055, respectively; Fig. 5b; right-tailed Fisher test). In humans, disruptions of genes within the same neurodevelopmental pathway cause a similar pattern of pleiotropic phenotypes^33^. Correspondingly, while no specific neuroanatomical abnormality was overrepresented among mouse models of orthologs belonging to the same human functional modules, an overall phenotypic convergence was observed for modules 2, 7, and 8 (Fig. 5d; Supplementary Notes) revealing neurodevelopmental pathway/phenotype relationships. Specifically, mouse orthologs of modules 2 and 7 yielded more similar brain abnormalities than other NAP genes across Bregma +0.98 mm but not Bregma −1.34 mm, while the opposite pattern was found for module 8 orthologs (Fig. 5d). Congruently, while genes from human modules 2 and 7 are more strongly expressed across tissues mapping to Bregma +0.98 mm than Bregma −1.34 mm, those from module 8 are more highly expressed across Bregma −1.34 mm tissues (Fig. 5c).

FMRP-associated genes were found in excess in modules 0 and 7 (Fig. 5b). We hypothesized that inactivation of mouse *Fmr1* gene itself would be associated with brain defects. Human patients show a larger striatum and a tendency toward large head size^34^. However, previous mouse MRI studies of *Fmr1*^*−/Y*^ revealed differences depending on the genetic background strain. *Fmr1*^*−/Y*^ C57BL/6J did not exhibit neuroanatomical defects^35^, yet *Fmr1*^*−/Y*^ FVB revealed a smaller striatum^36^. To overcome these discrepancies, we studied an *Fmr1*^*−/Y*^ model on a mixed C57BL/6J/FVB background using techniques developed in this study (Supplementary Notes). We detected a significant enlargement of the cingulate cortex (+18.8%, *p* = 7.6E-04; LMM) and the hippocampus (+20%, *p* = 0.03; LMM) (Fig. 5e), while the total brain area was marginally increased (+4.3%, p = 0.08; LMM). A separate sagittal sectioning protocol that examined 22 additional brain regions showed increased dentate gyrus and pyramidal area of the hippocampus (+14.4%, *p* = 8.3E-04 and +24.2%, *p* = 1.7E-03, respectively; LMM) (Supplementary Fig. 8).

Finally, we assessed whether mouse NAP genes are associated with whole-body traits. Among 26 categories of traits tested (MGI), 3 showed excess in behavioral/neurological, growth, and adipose traits (BH-p = 0.039, 0.0007 and 2.92E−05, respectively; right-tailed Fisher test) (Supplementary Fig. 9). This was confirmed when removing known-ID genes; however, we were unable to detect an association between local neuroanatomical defects and specific behavioral measures. All together, these mouse findings provide mechanistic insights into known disease-causing genes and simultaneously provide in vivo models for validation and interpretation of future cognitive disorders, such as ID.

## Discussion

Our study shows the importance of performing systematic neuroanatomical characterization in mutants as it offers the first anatomical genotype/phenotype map of the mouse brain with the identification of 198 NAP genes covering a variety of brain malformations. The commissures are most affected and microcephaly is more common than macrocephaly, in agreement with human studies^37^.

Our findings are important in at least four respects. First, 94% of NAP genes have never been previously associated with mouse brain anatomy. Assessment of brain sections usually relies on qualitative evaluation by the human eye, for example, corpus callosum agenesis or hydrocephalus. Our quantitative and ultra-standardized approach allows us to detect many more features and more subtle phenotypes. For example, our analysis of the mouse model of Fragile X is the first to recapitulate the human brain overgrowth defects^38^ with a larger hippocampal size of about 20%. It is possible that our stringent criteria for evaluating brain symmetry and positioning increased the sensitivity to detect phenotypes where other neuroanatomical studies have not, especially for the hippocampus whose size depends largely on these two criteria.

Our study indicates that genetic mutations causing neuroanatomical defects impact the overall equilibrium of the brain architecture. This is consistent with the fact that about half of NAP genes had a global impact on brain morphology. For example, ablation of *Rnf10*, which was recently linked to neuronal synaptic activity via its interaction with GluN2A subunit of NMDA receptors^39^, perturbs the whole brain. Physical pressures also exist in the brain and the most obvious example is hydrocephalus. When hydrocephalus develops, as the ventricles expand the distance between them decreases, impacting on the width of the corpus callosum.

A neuroanatomical baseline reference is provided for 991 genetically identical WTs on the C57BL/6N background, showing little inter-individual variation in most brain regions. However, the size of the amygdala, whose heritability was the lowest in a human study using twins^40^, displayed correspondingly higher variation in our WTs. This might explain our inability to detect genes associated with this structure. As a result, t-SNE 2D map grouped the amygdala with other undefined regions, but the vast majority of measures did cluster in coherent brain structures.

Second, our NAP genes converge onto a small number of groups (modules) of functionally similar genes participating in shared cellular pathways involving the actin and microtubule cytoskeleton, and the synapse, and the disruption of genes within the same module can yield a similar pattern of neuroanatomical abnormalities. While synapse biology is implicated in ASD^8^ and schizophrenia^9^, it is only emerging in ID^41^. Our study indicates that mechanisms confined to subcellular compartments as subtle as dendritic spines can translate into major neuroanatomical features. For example, *MAPK10*, a candidate gene for ID^26^, which yields a thick piriform cortex, has been shown to bind to a set of postsynaptic proteins^42^. *Anks1b*^*−/−*^ associated with a thickening of the dorsal hippocampal commissure has been implicated in the regulation of hippocampal synaptic transmission by controlling GluN2B subunit localization^43^ and reported as the most upregulated protein in synaptic fractions from *Fmr1*^*−/Y*^ mice^44^. These examples highlight that our measurements expose defects in neuronal circuitry and synaptic plasticity. Our study reveals an enrichment of NAP genes toward FMRP targets and describes distinct spatiotemporal expression signatures that follow a caudal to rostral direction. This suggests that the sub-compartmentalization of brain structures^45^ is under strong genetic control.

Third, our in vivo mouse models show a great capacity for novel disease gene validation with 83% of NAP genes not yet linked to any cognitive disorders, constituting a new pool of candidate genes testable against human diseases. During the course of our study, several of our mouse models for previously unknown NAP genes (for example, *Kptn*, *Donson*, or *Aff3*) were subsequently associated with causative mutations in recent human studies^46–48^, suggesting that other genes identified in this study may be validated in future studies. Also, it is noteworthy that the rate at which new cases are diagnosed remains low despite most cases being genetic in nature^29^. Ultimately, our models offer a tangible chance of improving this discovery rate, addressing the problem of missing pathogenicity that most large-scale sequencing studies encounter and helping with disease stratification.

Importantly, our study justifies the use of brain anatomy as a common denominator of cognition, offering endophenotypes for neurological or psychiatric disorders. For example, mutations of *SLITRK4* have been recently linked to schizophrenia through a synaptic dysfunction mechanism^49^. In our study, *Slitrk4*^*−/Y*^ displayed a smaller somatosensory cortex, in line with the underpinnings of sensory processing deficits in patients with schizophrenia. Our resource can also help decipher the etiology of several important human birth defects causing perinatal or in utero death and for which knowledge is lagging behind because of ethical considerations and/or lack of biological material. Only four genes (*AP1S2*, *CCDC88C*, *L1CAM*, and *MPDZ*) have been associated so far with congenital hydrocephalus, a pathology affecting 1:1000 live births^50^. Our top candidate gene for congenital hydrocephalus is *Atad3a*, associated with severe enlargement of the ventricles in the heterozygous state and previously reported to be lethal when homozygously deleted^51^. Interestingly, Harel and colleagues have recently reported a biallelic deletion of *ATAD3A* associated with infantile lethality^52^.

In conclusion, our study represents the largest atlas of the causal link between gene mutation and its associated neuroanatomical features to date. It contributes a breadth of new knowledge on the genetics of brain morphogenesis and their underlying networks, providing evidence as to the importance of the cytoskeleton and the synapse in how the brain forms its structures in a highly specific spatiotemporal manner, and adequately complements human genetic studies of neurodevelopmental and neuropsychiatric disorders.

## Methods

We provide a summary of our main methods below. A detailed description and any associated references are available in Supplementary Notes.

### Study samples and gene selection

A total of 6214 mice were analyzed corresponding to 1566 allelic constructions for 1446 unique genes. Information related to the construction of the mutant line such as allele, genotype, promoter, cassette, diet, and background strain is provided in Supplementary Data 5. Most mouse mutants were generated using the Knockout-first allele method^15^. The strategy relies on the identification of an exon common to all transcript variants, upstream of which a LacZ cassette was inserted to make a constitutive knockout. A subset of mutants were generated using CRISPR/Cas9 methodology, similar to previously reported method^53^. Genes were selected for entry into the pipeline from requests by WTSI faculty and collaborators working on a wide variety of fields, including cancer, metabolism, infection, immunology, behavior, and human evolution, but without a priori interest in the data generated from the brain histopathology screen. In addition, genes without prior characterization were assigned by the IMPC to WTSI for phenotyping. Assessed genes were distributed on all chromosomes except Y with no bias on their genomic distribution. One hundred and five genes were studied multiple times for validation purposes (see Supplementary Data 2 for details).

### Data collection

Supplementary Fig. 1 shows the overall experimental workflow. All standard operating procedures are described in more detail elsewhere^17,18^. In general, brain samples were immersion-fixed in 10% formalin for at least 48 h, before paraffin embedding and sectioning at 5-μm thickness. Three coronal and one sagittal sections were stereotatically defined and named as critical sections. These critical sections (Bregma +0.98 mm, Bregma −1.34 mm, Bregma −5.80 mm, and Lateral 0.60 mm) were double-stained (Luxol Fast Blue for myelin and Cresyl violet for neurons) and scanned at cell-level resolution using the Nanozoomer whole-slide scanner 2.0HT C9600 series (Hamamatsu Photonics, Shizuoka, Japan). A total of 85 co-variates, for example, sample processing dates and usernames, were collected at every step of the procedure (Supplementary Data 3), as well as 118 brain morphological parameters of 77 area and 39 length measurements and the number of cerebellar folia (Supplementary Data 4 and Supplementary Fig. 2). All samples were also systematically assessed for cellular ectopia (misplaced neurons).

### Quality control

Brain images were systematically assessed to keep track of potential drifts and ensure high quality. Each image was scored based on four criteria: (1) suitability for analysis, (2) adequacy of the intensity and contrast of the staining to properly delineate brain structures, (3) symmetry, and (4) sectioning precision. By contrast to more conventional histopathological screens that often rely on qualitative assessment, we used a quantitative approach where each section had to pass well-defined stereotaxic coordinates defined according to the Mouse Brain Atlas^54^ before image analysis. To do this, we recorded how close the image to be analyzed was to the critical section (that is, at the precise stereotaxic position) (Supplementary Fig. 5). Data quality being crucial for the interpretation of large-scale projects, a thorough quality-control process was designed and implemented at multiple steps of the experimental procedure. A lot of care was given to control human errors and false-positive findings using an in-house quality-control pipeline within a FileMaker Pro framework (Supplementary Fig. 5). A semi-manual stepwise approach was implemented to standardize and facilitate data cleaning process.

### Statistical framework for gene identification

To account for temporal variation that may have occurred since the start of the study in 2009, we used a LMM framework computing the necropsy date of the animal as a random variable. The model was fitted in R using a parallelized version of PhenStat (version 2.2.4), a package developed for statistical analysis of large-scale phenotypic data from the IMPC^20^. Supplementary Data 10 provides association and percentage change data for 1566 assessed alleles across 48 left and right combined for coronal and 40 for sagittal parameters. Relevant Mammalian Phenotype (MP) terms were used to describe neuroanatomical defects, and when needed, new MP terms were created specifically for this project through collaboration with the Jackson Laboratory (http://www.informatics.jax.org/) (Supplementary Data 8). Multiple testing corrections were performed using the Benjamini–Hochberg (BH) method. From this, five gene lists were generated based on 1, 5, 10, 15, and 20% false discovery rate (FDR) (see column C in Supplementary Data 10). To control for FDR, a set of 100 permutations was run within each subproject. The average number of significant genes in a set of 100 permutations was compared to the results of the true data. Gene association was considered as significant when the adjusted *p* value was <0.1.

### Data imputation and imputed datasets

A newly developed imputation tool (PHENIX version 1.0^24^), based on a Bayesian multiple-phenotype mixed model method, was used to overcome the problem of missing data in downstream analyses that relied on full datasets. This resulted in a new imputed dataset made of 1380 alleles from 1306 unique genes, totaling 5281 mouse samples (Supplementary Data 12). The same statistical pipeline as the one applied to non-imputed data was used (Supplementary Data 13), producing a new gene-association list (Supplementary Data 14).

### Genes causing strongest and weakest NAP

*Z*-score deviations of mutant gene replicates from the average of matched controls were estimated for each brain parameter list (Supplementary Data 14). Considering only the 960 mutant genes with 1:1 human orthologs, we selected the 10% of genes associated with the highest (top) and lowest (bottom) absolute *z*-score values across features (*n* = 96). We formed 1000 permuted sets of top 10% genes by randomly drawing the same number of genes from human mutant orthologs (*n* = 96). Consistent results were found using 1%, 5 and 15% top and bottom, and when defining them using cumulative absolute *z*-scores across brain parameters, although the signals were weaker.

### Phenotypic similarity

Considering 1:1 human orthologs of NAP genes, we re-scored significant abnormalities (BH-p ≤ 0.05) as 1 (presence) or otherwise 0 (BH-p > 0.05; absence) for each gene and brain parameter. The similarity of defects caused by the disruption of pairwise gene combinations was calculated using a simplified version of the *Goodall3* index^55^. Given the low incidence of significant abnormalities post BH correction (i.e., mean of 0.23 ± 0.92 defects per gene), we examined the shared presence of abnormalities between gene pairs, weighted by their overall frequency. This simplification generated a more conservative index, whereby strong similarity was not driven by genes lacking neuroanatomical defects. The new similarity index was given by:

$$S(i,j)_k = \left\{ {\begin{array}{*{20}{c}} {1 - f_k^2,} {{\mathrm{if}}\;k\;{\hbox{is present in both genes}}} \\ {0,} {{\mathrm{if}}\;k\;{\hbox{is absent in both or present in one}}} \end{array}} \right.$$

where *S*(*i,j*)_*k*_ is the Goodall3 measure between genes *i* and *j* for brain feature *k* and *f*_*k*_ is the proportion of NAP orthologs associated with abnormalities in *k*. The overall phenotypic similarity of two human NAP orthologs was estimated as the average similarity across Bregma +0.98 mm and −1.34 mm.

### Gene networks

The mouse and human gene networks downloaded or constructed (and associated references) are summarized in Supplementary Data 21. We downloaded MouseNet v2, a functional mouse gene network incorporating various mouse -omics resources (i.e., PPI, gene expression data, functional annotations, and homology data)^56^. We constructed human body-wide and brain spatiotemporal gene co-expression networks, using GTEx^57^ and BrainSpan^58^ RNA sequencing data across 51 bodily tissues and across 27 brain tissues spanning 12 developmental stages, respectively. Genes with FPKM (Fragments Per Kilobase of transcript per Million mapped reads) or RPKM (Reads Per Kilobase of transcript per Million mapped reads) values <1 in >95% of samples were excluded and gene co-expression networks were derived by estimating the correlation of expression patterns of pairs of protein-coding gene across all associated samples, using Pearson’s coefficients^59^. Human PPI networks were downloaded from String^60^. Lastly, by combining various human genomic datasets (i.e., PPIs, GO, MGI, KEGG, Reactome, and gene expression data), we constructed an integrated gene network, termed PLN, wherein pairs of gene are assigned scores, reflecting their likelihood of functional interaction, based on multiple lines of evidence^32^.

### Clustering and module identification

To examine the network interconnectedness of a set of genes, we compared the sum of their network links to that of permuted gene sets, constructed by randomly sampling the same number of human genes, matched for CDS and network connectivity^32^. By running 1000 permutations, we derived an empirical *p* value, reflecting the fraction of randomized gene sets, which are more interconnected in the evaluated network, than the set of interest. We identified modules of strongly interconnected genes by partitioning gene networks using the Louvain algorithm^61^. This greedy optimization method attempts to maximize the modularity of a network division (strength of connections inside modules as compared to between modules). The algorithm was implemented in Gephi using a resolution parameter of 1.

### Ethical considerations in animal use

The care and use of mice in the Wellcome Sanger Institute study was carried out in accordance with UK Home Office regulations, UK Animals (Scientific Procedures) Act of 1986 under two UK Home Office licenses (80/2485 and P77453634) that approved this work, which were reviewed regularly by the Wellcome Sanger Institute Animal Welfare and Ethical Review Body.

### Reporting summary

Further information on research design is available in the Nature Research Reporting Summary linked to this article.

## Supplementary information

Supplementary Information

Peer Review

Reporting Summary

Description of Additional Supplementary Files

Supplementary Data 1

Supplementary Data 2

Supplementary Data 3

Supplementary Data 4

Supplementary Data 5

Supplementary Data 6

Supplementary Data 7

Supplementary Data 8

Supplementary Data 9

Supplementary Data 10

Supplementary Data 11

Supplementary Data 12

Supplementary Data 13

Supplementary Data 14

Supplementary Data 15

Supplementary Data 16

Supplementary Data 17

Supplementary Data 18

Supplementary Data 19

Supplementary Data 20

Supplementary Data 21

## Data Availability

We have provided all the data produced in this study within the article and its associated supplementary information and data files.

## Code availability

All bioinformatics analyses for this study were performed using R. PhenStat and PHENIX packages are available for free download from https://www.bioconductor.org/packages/devel/bioc/html/PhenStat.html and https://mathgen.stats.ox.ac.uk/genetics_software/phenix/phenix.html. In-house R scripts used for the identification of genes are available upon request.
