# Supplementary material for: Large-scale neuroanatomical study uncovers 198 gene associations in mouse brain morphogenesis
Source: Nat Commun. 2019 Aug 1;10:3465. doi: 10.1038/s41467-019-11431-2 (PMC6671969; doi:10.1038/s41467-019-11431-2)
Supplement: Supplementary file 1 — Supplementary Information [file 41467_2019_11431_MOESM1_ESM.pdf]

SUPPLEMENTARY INFORMATION

Large-scale neuroanatomical study uncovers 198 gene associations in mouse brain morphogenesis

Stephan C. Collins,[1,2,3,4,5,12] Anna Mikhaleva,[6,12] Katarina Vrcelj,[7,12] Valerie E. Vancollie,[8,12] Christel Wagner,[1,2,3,4] Nestor Demeure,[1,2,3,4] Helen Whitley,[1,2,3,4] Meghna Kannan,[1,2,3,4] Rebecca Balz,[6] Lauren F.E. Anthony,[8] Andrew Edwards,[9,10] Hervé Moine,[1,2,3,4] Jacqueline K. White,[8] David J. Adams,[8] Alexandre Reymond,[6] Christopher J. Lelliott,[8] Caleb Webber,[7,11] and Binnaz Yalcin[1,2,3,4,6,*]

[1]Institut de Génétique et de Biologie Moléculaire et Cellulaire, 67404 Illkirch, France
[2]Centre National de la Recherche Scientifique, UMR7104, 67404 Illkirch, France
[3]Institut National de la Santé et de la Recherche Médicale, U964, 67404 Illkirch, France
[4]Université de Strasbourg, 67404 Illkirch, France
[5]Inserm UMR1231 GAD, University of Bourgogne Franche-Comté, 21000 Dijon, France
[6]Center for Integrative Genomics, University of Lausanne, CH-1015 Lausanne, Switzerland
[7]Department of Physiology, Anatomy and Genetics, University of Oxford, OX1 3PT Oxford, United Kingdom
[8]Wellcome Trust Sanger Institute, Hinxton, CB10 1SA Cambridge, United Kingdom
[9]Woodland view Hospital, NHS Ayrshire and Arran, Irvine KA128SS, Scotland
[10]Wellcome Trust Centre for Human Genetics, OX3 7BN Oxford, United Kingdom
[11]UK Dementia Research Institute, University of Cardiff, CF24 2HQ Cardiff, United Kingdom
[12] These authors contributed equally

[*]Correspondence: Binnaz.Yalcin@igbmc.fr

TABLE OF CONTENTS

SUPPLEMENTARY FIGURES 3

SUPPLEMENTARY NOTES 14

EXPERIMENTAL MODEL AND SUBJECT DETAILS 14

ANIMALS, HOUSING AND HUSBANDRY 14
STUDY SAMPLES 16
BRAIN COLLECTIONS AND QUALITY CONTROL 18
CORONAL SIX-WEEK OLD DATASET 20
CORONAL SIXTEEN-WEEK OLD DATASET 20
SAGITTAL SIXTEEN-WEEK OLD DATASET 21
FMR1$^{-/Y}$ MOUSE MODEL 21
DATA COLLECTION 22
DEVELOPMENT OF A RELATIONAL DATABASE 22
IMAGE QUALITY CONTROL 26
DATA QUALITY CONTROL 28

QUANTIFICATION AND STATISTICAL METHODS 30

STATISTICAL FRAMEWORK FOR GENE IDENTIFICATION 30
DATA IMPUTATION AND IMPUTED DATASETS 35
INTER-REGIONAL BRAIN RELATIONS 37
T-DISTRIBUTED STOCHASTIC NEIGHBOR EMBEDDING 37
GENE LISTS ENRICHMENT 38
GENE EXPRESSION DATASETS 41
GENE NETWORKS 44
NETWORK CLUSTERING 44
MODULE IDENTIFICATION 45

SUMMARY OF KEY RESOURCES TABLE 46

SUPPLEMENTARY REFERENCES 48

SUPPLEMENTARY FIGURES

Supplementary Figure 1

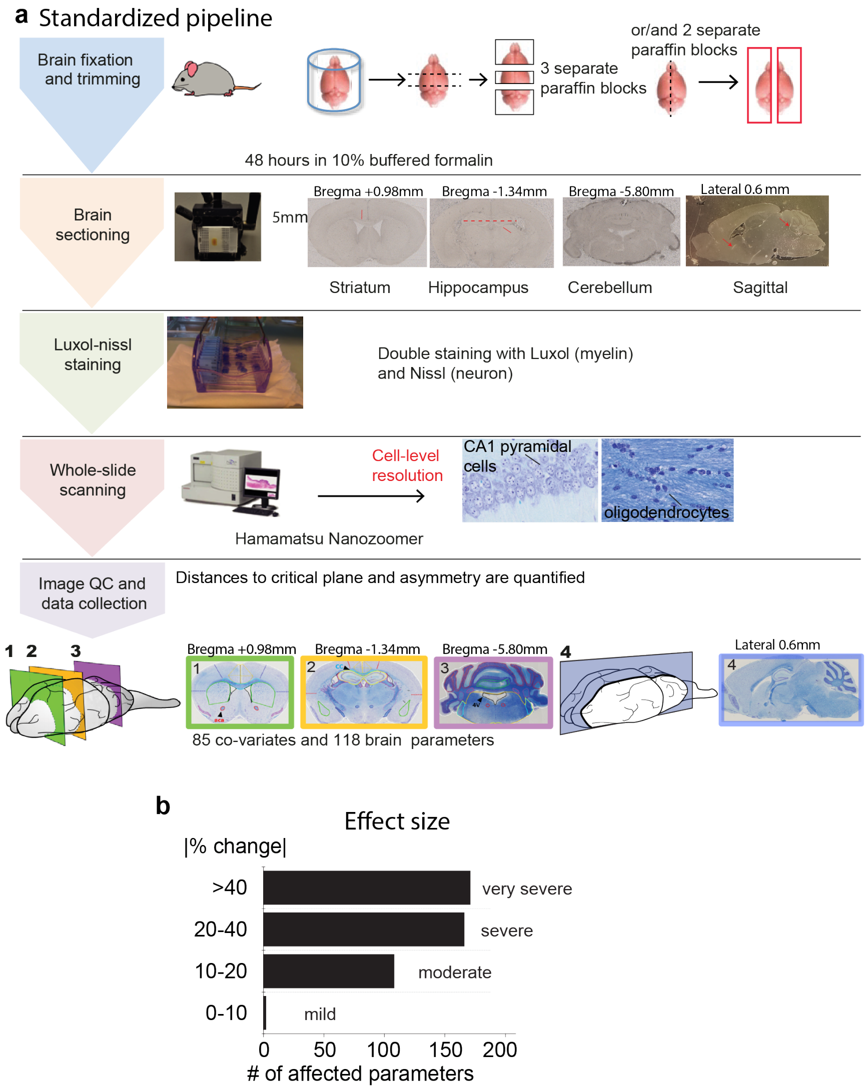

Supplementary Figure 1. Standardized workflow for phenotyping of the adult mouse brain (a) The experimental procedure consists of five main steps. Each brain is fixed in 10% buffered formalin for at least 48 hours and trimmed for paraffin embedding (step 1). Careful and precise sectioning is a prerequisite in this protocol, sections must be symmetrical and match well defined anatomical features (step 2). Then, each section is double-stained using Nissl (violet) for neurons and Luxol (blue) for myelin, generating a color contrast between white and grey matter (step 3). Whole slides are scanned using a digital slide Hamamatsu Nanozoomer scanner (2.0HT C9600 series), producing whole brain images at cell-level resolution (step 4). Finally, the data is collected using a set of 85 experimental co-variates (**Supplementary Data 3**) and 118 brain parameters (**Supplementary Data 4**; **Supplementary Fig. 2**), and extensive quality control checks and critical evaluation of the entire dataset are carried out (step 5). **(b)** Classification of affected structures based on percentage change (absolute value) relative to WTs as mild, moderate, severe or very severe. For more details, see **Supplementary Notes**.

Supplementary Figure 2

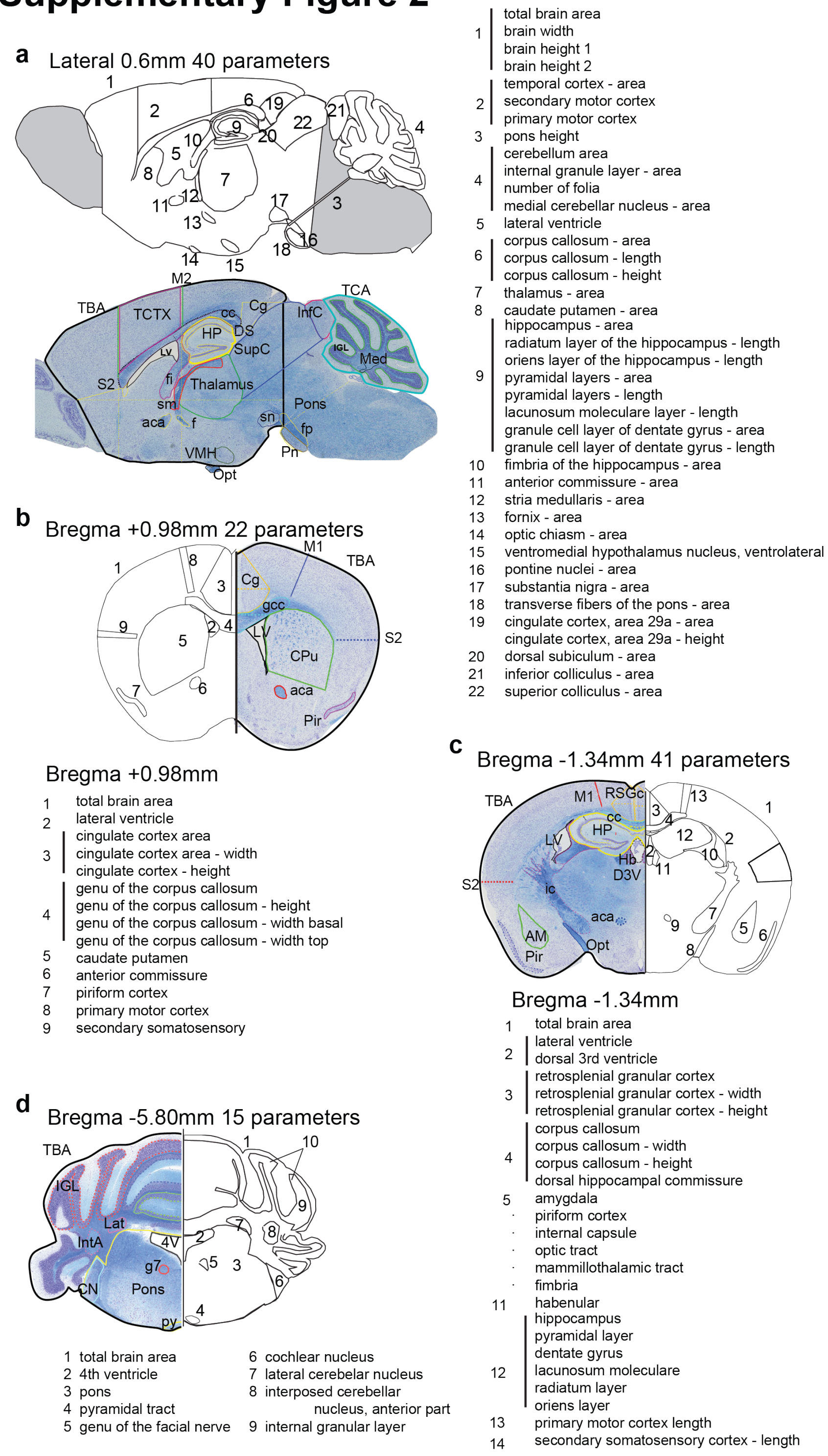

Supplementary Figure 2. Assessed neuroanatomical parameters A total of 118 brain parameters were quantified across three stereotactically defined coronal sections and one sagittal section (descriptive statistics in **Supplementary Data 4**). 40, 22, 41, and 15 parameters are measured at Lateral 0.60mm, Bregma +0.98mm, Bregma -1.34mm, and Bregma -5.80mm, respectively. Numbers correspond to established neuroanatomical structures and are occasionally broken down in several measurements, for example, the hippocampus is characterized by 8 independent measurements at Lateral 0.6mm.

Supplementary Figure 3

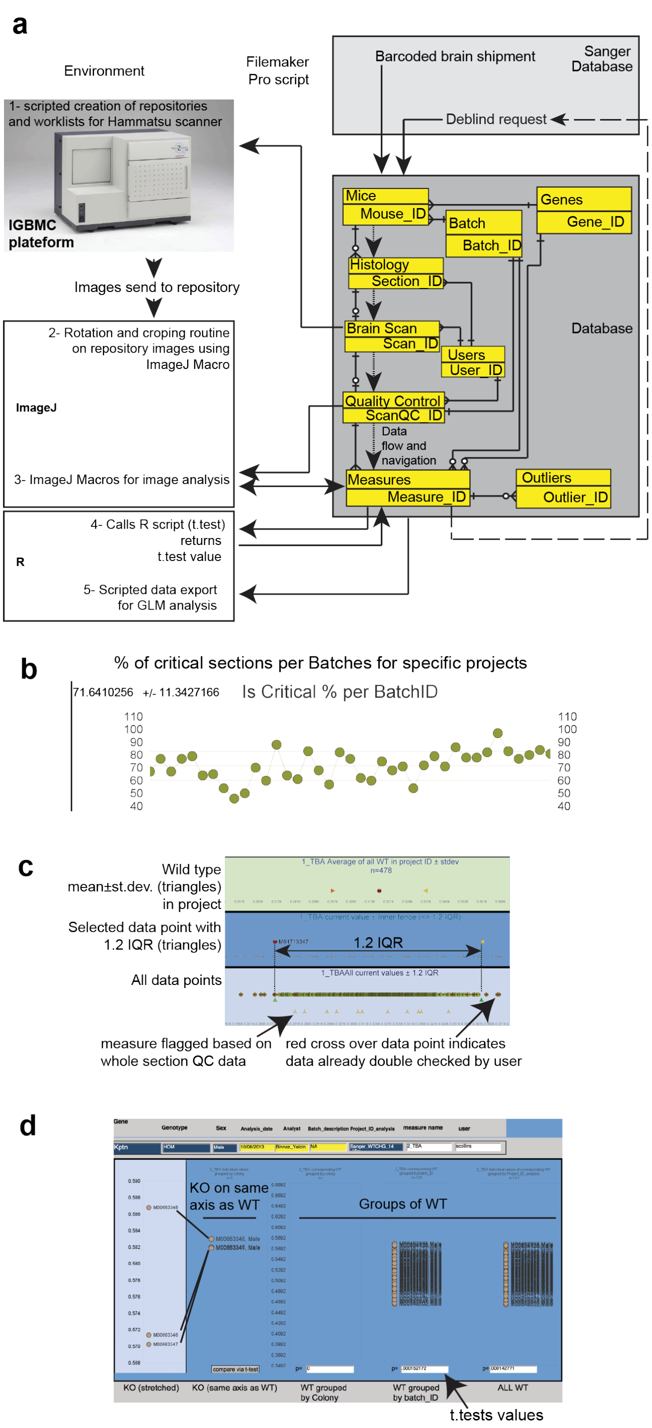

Supplementary Figure 3. Database (a) Relational database design and quality control interface. The general design of the database (dark grey box) consists of nine tables each containing a primary key. Only the main relationships are indicated using E-R diagram conventions (O: zero, -: one, <: many). A navigation bar on each layout and table was designed according to the workflow (dotted arrows) of the histological pipeline to enhance the user interface. The database is able to interact with its environment through a combination of FileMaker and other software (ImageJ and R) scripts. **(b)** As part of the quality control tools for management purposes, this screen capture shows the percentage of critical sectioning achieved by batches of brain sections. Similar graphs are produced for other quality control checks such as staining quality, brain symmetry and image quality. The list of lab members who have prepared the samples may be listed on screen (but not shown here) allowing troubleshooting if quality falls below acceptable standards. **(c)** A screen shot of a data curation process. A FileMaker Pro script detects all data points falling outside the 1.2 interquartile range (IQR) for a given batch or project (user definable) for all or a subset of parameters and flags the suspicious measures unless it has been manually validated previously. The user may then validate each flagged outlier by the click of a button, which activates an ImageJ macro opening the picture analyzed and the ROIs (regions of interest), which need verification. If changes are made to the ROI, the new ROI coordinates are saved together with new measures in the database. **(d)** Implementation of Student's t-tests within the FileMaker Pro relational database

Supplementary Figure 4

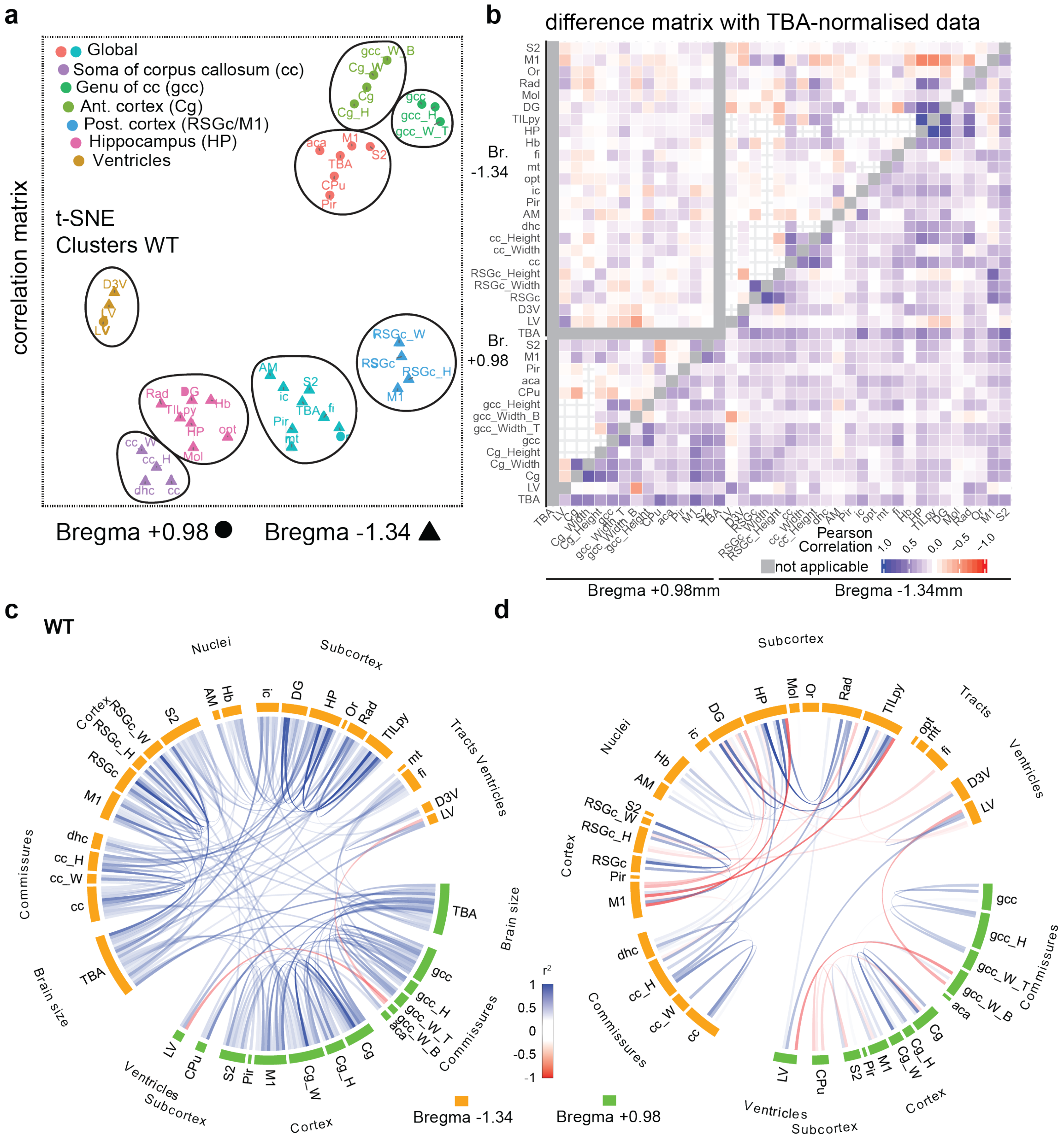

Supplementary Figure 4. Neuroanatomical correlation matrices and t-SNE (a) T-SNE clusters: t-Distributed Stochastic Neighbor Embedding 2D-map based on phenotypic similarities in WTs. **(b-d)** Pearson correlations between brain parameters in WTs. **(b)** Difference matrix of TBA normalized Pearson correlation coefficients subtracted from non-normalized WTs (upper triangle), and Pearson correlation coefficients from non-normalized WTs (lower triangle). Parameters are from coronal sections only (largest data set) at Bregma +0.98mm and -1.34mm. **(c)** Top one hundred correlations between brain parameters in WTs shown on a chord diagram. Parameters are displayed around the circle, separated into 2 sections (green indicates section 1 at Bregma +0.98mm and orange section 2 at Bregma -1.34mm). **(d)** Top one hundred correlations from TBA normalized Pearson correlation coefficients subtracted from non-normalized WTs. Blue and red arcs/boxes denote positive or negative correlations, respectively. The strength of the correlation is color-coded. Acronyms are described in **Supplementary Data 4**.

Supplementary Figure 5

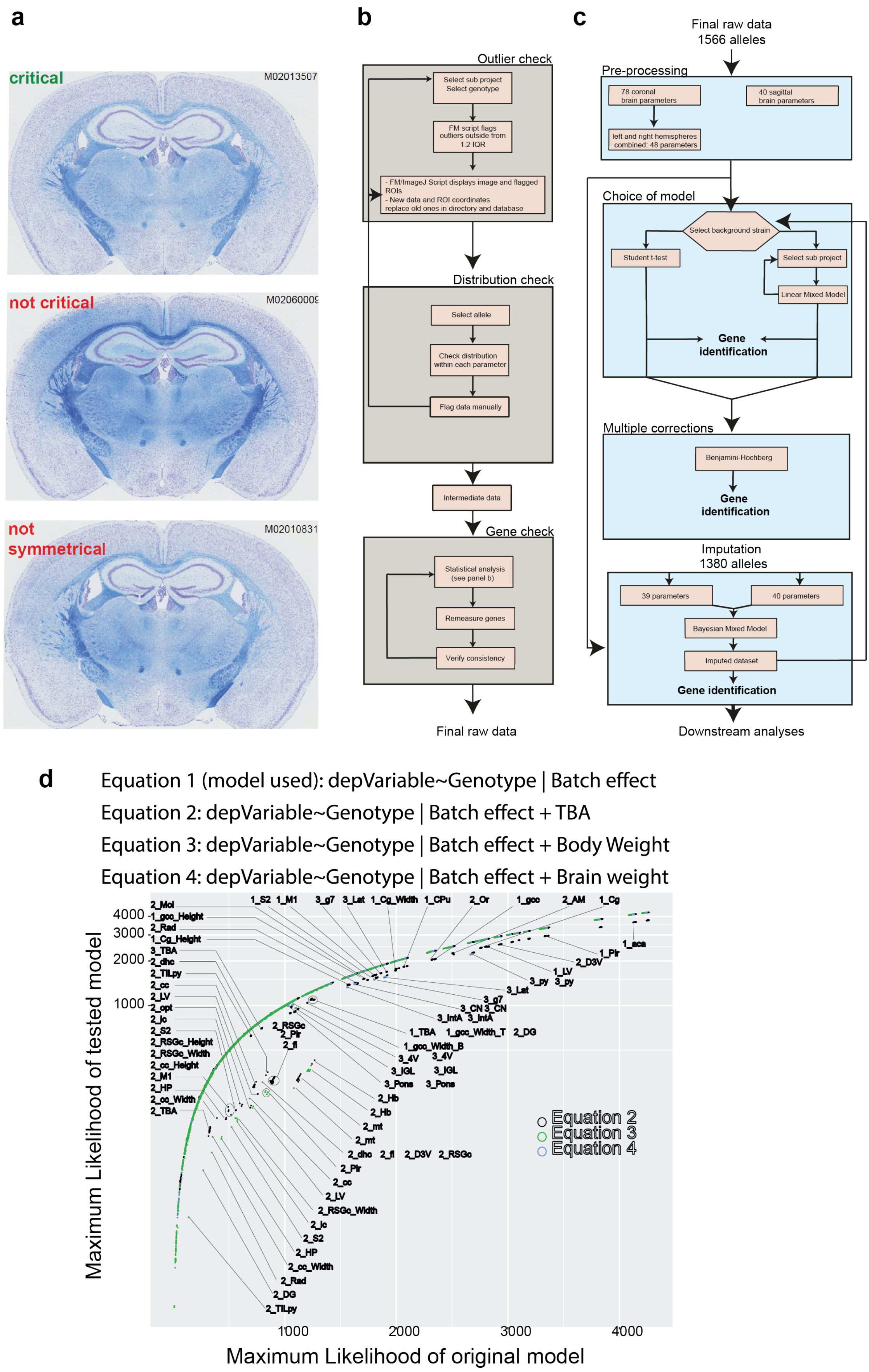

Supplementary Figure 5. Quality control, statistical pipeline and model validation (a) Image quality control. Top: Brain image that matches perfectly the defined position at Bregma -1.34mm and is symmetrical. Middle: Brain image that is not critical at a distance of 120μm posterior to the critical section. Bottom: Brain image that is critical on the right hemisphere, but not on the left hemisphere revealing a small asymmetry of about 60μm anterior to the critical section. **(b)** Data quality control was performed using a standardized framework. Data were first checked for outliers falling outside a 1.2 IQR rule. For each gene and parameter, data distribution was then manually checked against batch and colony matched controls. Focusing on NAP genes, a new experimenter (always the same person) systematically re-measured each parameter in an effort to identify any potential experimenter biases. **(c)** The statistical framework consisted of four steps. 1) Pre-processing: To analyze all genes with respect of their genotype and construction, a variable collating gene, genotype and construct information was created from the raw data. 2) Choice of model: A linear mixed model was fitted in R (PhenStat package[1]) using the necropsy date as a random temporal variablet. 3) Multiple correction testing was applied using the Benjamini Hochberg method in an effort to control the false positive rate. 4) Imputation: a Bayesian multiple-phenotype mixed model method was used to overcome the problem of missing data in the downstream analyses. **(d)** Co-variate structure was tested for effect and the resulting Maximum Likelihood Estimate (MLE) was plotted against the MLE of mixed models without co-variates. Each point corresponds to a pair of allele and phenotype combination where X corresponding to the MLE of Equation 1 and Y to MLE of tested equation. The use of a logarithm scale was chosen to allow enough place to indicate the names of parameters affected by co-variates. For more details, see **Supplementary Notes**.

Supplementary Figure 6

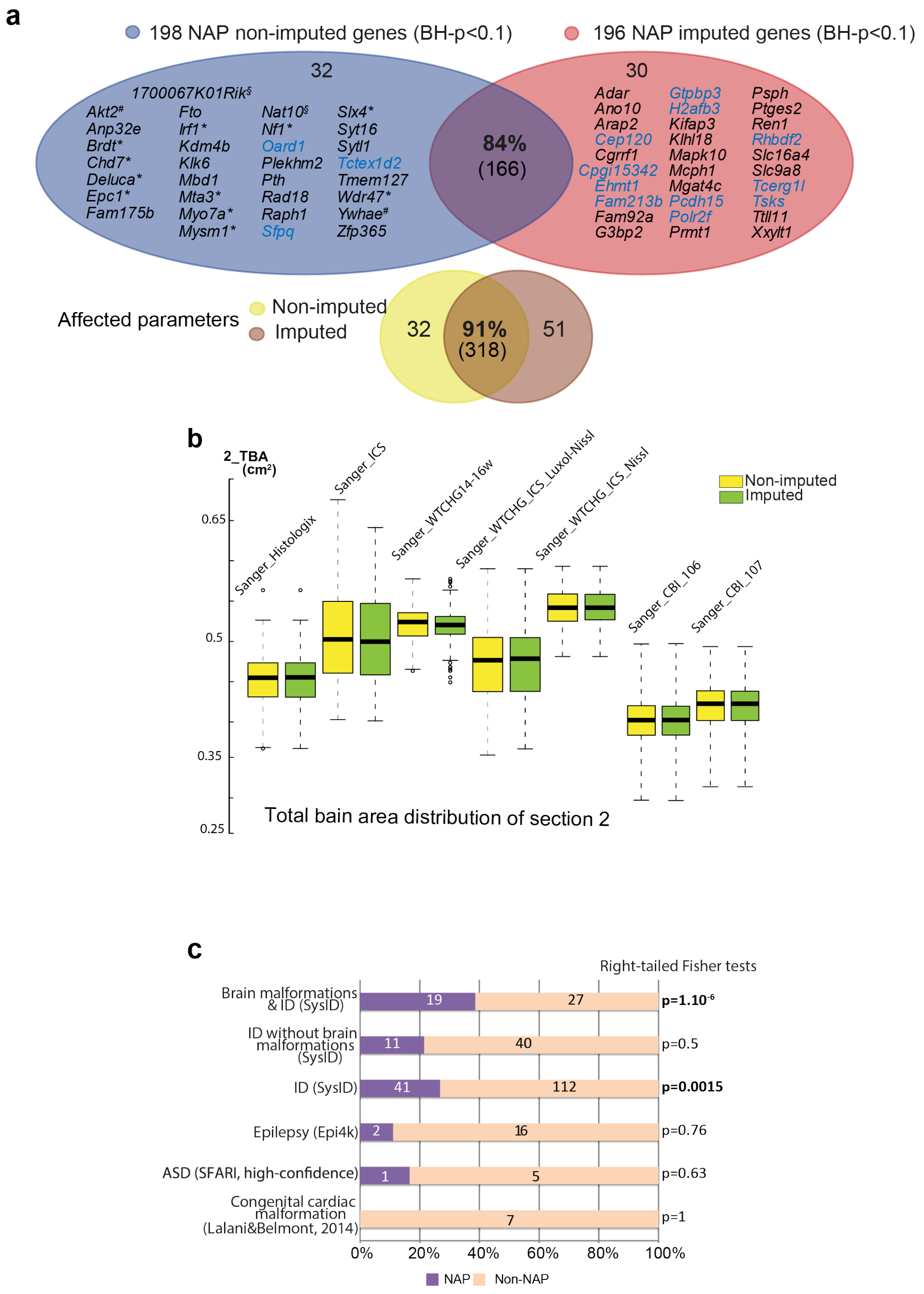

Supplementary Figure 6. Imputation and human disease gene lists (a) Assessment of genes and parameters in the imputed dataset. The overlap is shown for both NAP genes and parameters between imputed versus non-imputed for adjusted p-value threshold of less than 0.1. **(b)** Assessment boxplots of brain size distribution in the imputed versus non-imputed data across the various subprojects. **(c)** Enrichment analysis of mouse NAP genes (this study) with various studies of neurological disorders: SysID[2] (June 2018 download), Epi4K (https://www.epi4k.org/), SFARI (https://www.sfari.org/) and congenital cardiac malformatitons used as negative control[3].

Supplementary Figure 7

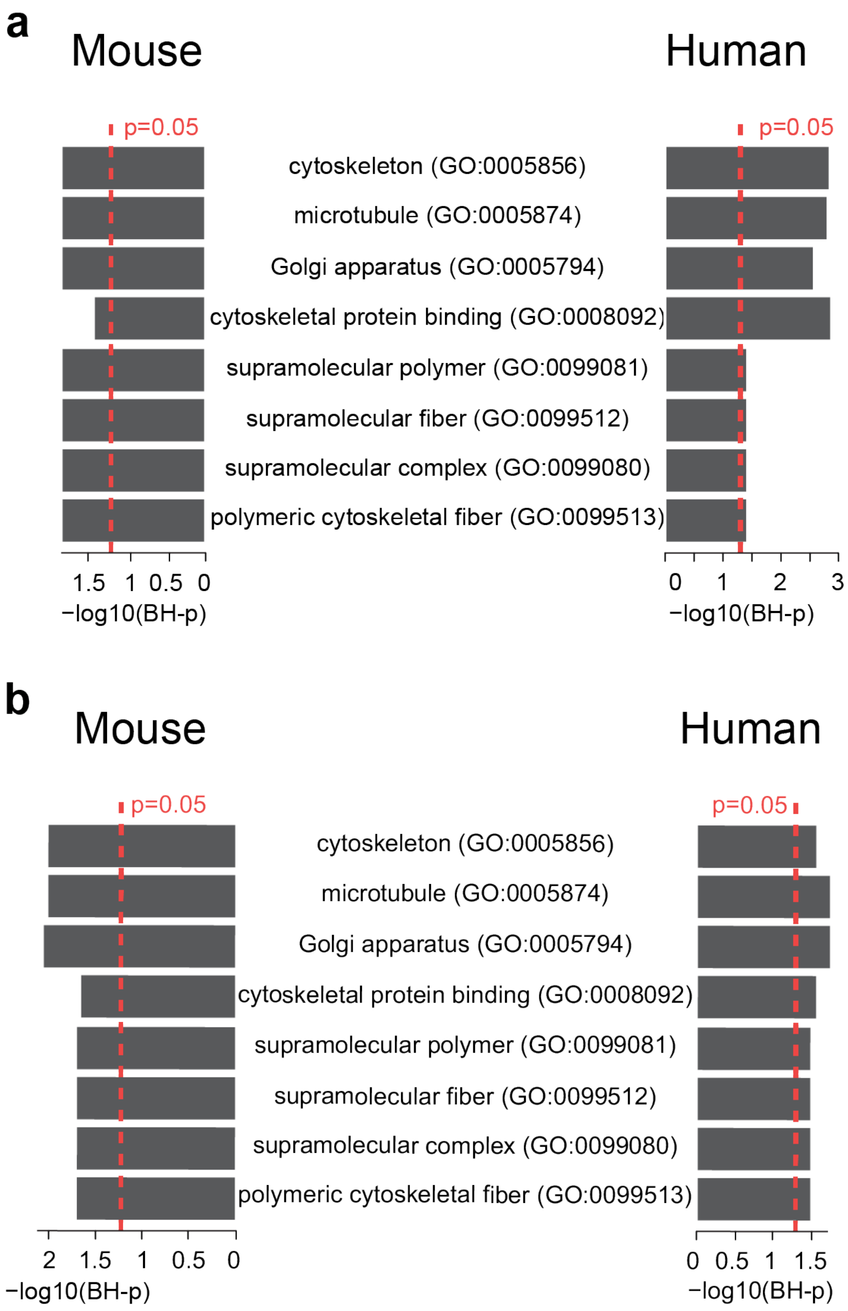

Supplementary Figure 7. GO enrichment analysis GO enrichment analyses for mouse NAP genes and their human 1:1 orthologues. Left: GO terms, relating to neuronal functions, over-represented among mouse NAP genes compared to all remaining genes annotated in the GO (**a**) and non-NAP genes (**b**). Right: Similar analysis using human 1:1 orthologues. Significance was derived using right-tailed hypergeometric tests. The x-axes depict the p-values adjusted for the number of GO categories tested using the Benjamini-Hochberg method.

Supplementary Figure 8

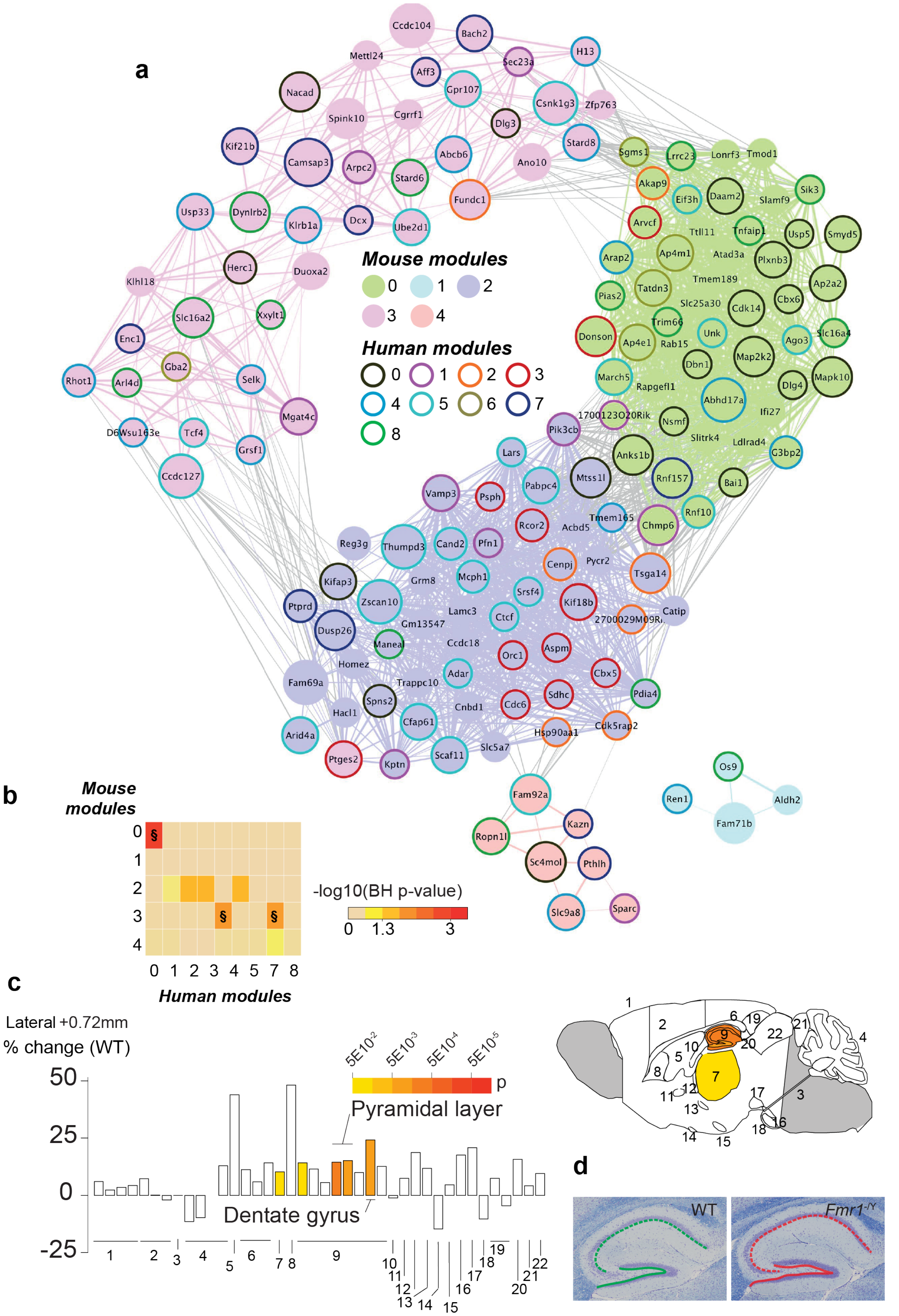

Supplementary Figure 8. Overlap between mouse and human gene modules (a) Overlap between the mouse gene modules (identified using a gene co-expression network, derived from mouse developing CNS and adult brain expression data) and human PLN modules. Shared node and border colours depict common memberships to mouse and human modules, respectively; the thickness of the edge is proportional to the co-expression coefficient of the respective genes. **(b)** Gene overlaps between mouse and human modules tested using right-tailed Fisher tests. Corrections for multiple testing performed using the BH method to control for the numbers of modules tested. **(c)** Neuroanatomical study of *Fmr1*$^{-/Y}$ in sagital plane (n=6 WT and n=6 *Fmr1*$^{-/Y}$, male, 8 week old). Top: Schematic representation of the affected brain structures at Lateral 0.72mm. Bottom: Histograms showing the percentage of increase or decrease of brain structures compared to matched controls. **(d)** A representative image is shown for *Fmr1*$^{-/Y}$ and matched wildtype, illustrating the enlarged length of the dentate gyrus and total pyramidal layer of the hippocampus. Unique sagittal regions and their associated parameters are listed accordingly to their reference number in **Supplementary Figure 2a**.

Supplementary Figure 9

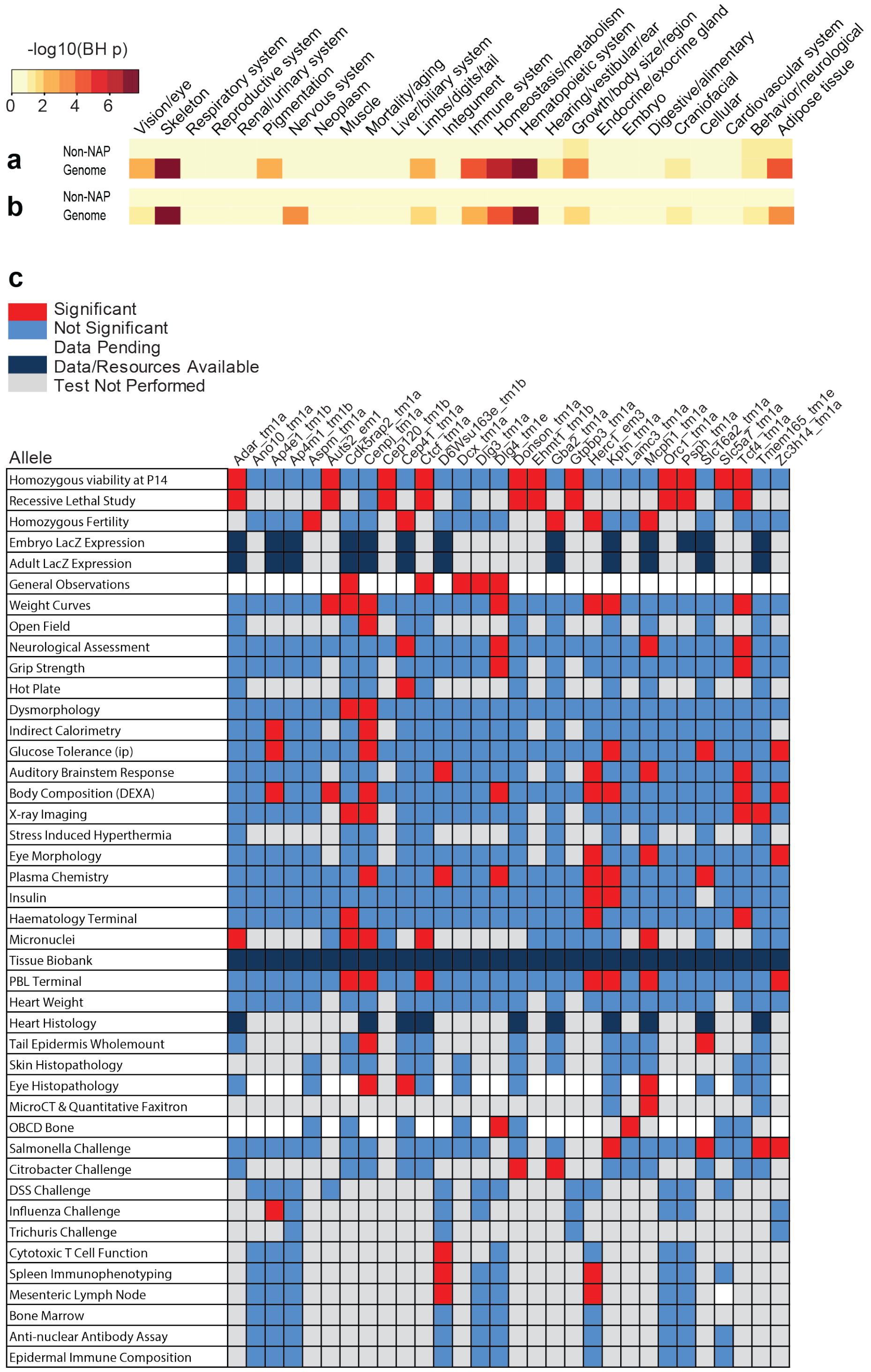

Supplementary Figure 9. Association of mouse neuroanatomical defects with whole body phenotypes (a) Association of NAP genes with 26 general categories of whole body traits versus 1) non-NAP genes and 2) all annotated genes in the database (data downloaded from MGI, July 2017). **(b)** Association of NAP genes after removal of the 30 known ID associated genes. **(c)** Whole body mouse phenotypes for 30 ID-associated genes among unique human NAP orthologues.

SUPPLEMENTARY NOTES

Supplementary notes are described in details below.

EXPERIMENTAL MODEL AND SUBJECT DETAILS

Animals, housing and husbandry

To ensure high comparability between mouse models, the mice used in this study came from a number of similar genetic background strains, a complete list of which can be found in **Supplementary Data 1**. We maintained most of the mice on a pure inbred C57BL/6N background (representing 80% of the lines tested in this study), or for early lines on mixed C57BL/6 backgrounds (18%; e.g., *C57BL/6N;C57BL/6Brd-Tyrc-Brd*), to minimize variation in screening results due to strain differences. Lines with other genetic backgrounds (2%; e.g., 129, CBA, C3Fe) were used at the start of the project to initiate the pipeline, and were always analyzed alongside strain-matched controls. For our main C57BL/6N background, a core colony was set up using mice from external providers (Taconic Biosciences), and the core colony nucleus was actively refreshed at set generational points (typically 10 generations) and cryopreserved to avoid genetic drift.

Most lines were initially phenotyped to produce data as part of the standardized pipelines from the Mouse Genetics Project (MGP) at the Wellcome Trust Sanger Institute (WTSI). Over the lifetime of the project, brains from 3 pipelines were collected, i.e. Brain (Necropsy dates May 2008 to October 2010), Mouse GP (April 2009 to February 2012) and MGP Select (February 2012-Current) pipelines. At the end of a pipeline, the mice were killed and brains collected for screening of brain histological

phenotypes. Brains from 3 male mice per mutant genotype were collected, which was estimated based on power calculation, limited throughput of both the necropsy collections and histological workflows and 3Rs in regards to animal use. In addition, up to 3 male control brains were collected weekly to form a WT baseline population for analysis. Formal randomization was not used to select mice for phenotyping; rather, each week mice were selected for phenotyping by identifying the age-correct mice from the breeding colonies supplying mutants. For WTs, usually 2 cages per sex were used for both the Mouse GP and MGP Select pipelines, normally from the largest breeding colony at that time point. For the Brain pipeline, 3 wild type brains were collected alongside mutant mice each week.

Mice phenotyped by the Mouse GP pipeline were given a HFD (high fat diet) from 4 weeks of age (Western RD, 829100, 21.4% crude fat content, 42% kcal as fat,~0.2% cholesterol Special Diet Services, Witham, UK)[4]. Mice phenotyped by the Brain and MGP Select pipelines were given a breeders chow (Mouse Breeder Diet 5021, 9% crude fat content, 21% kcal as fat, 0.276ppm cholesterol, Labdiet, London, UK) from weaning. All mice were given water and diet *ad libitum*, except during the fasting prior to either the intra-peritoneal glucose tolerance test (IPGTT) or, rarely, necropsy sessions, where access to diet was removed. Mice were maintained in a specific pathogen free unit with sentinel mouse monitoring on a 12hr light: 12hr dark cycle with lights off at 7:30pm and no twilight period. The ambient temperature was 21±2°C and the humidity was 55±10%. All animals were regularly monitored for health and welfare concerns and were additionally checked prior to and after procedures. Mice were typically housed for phenotyping using a stocking density of 3-5 mice per cage (overall dimensions of caging: (LxWxH) 365x207x140mm, floor area 530cm^2) in individually ventilated caging (Tecniplast Seal Safe1284L) receiving 60 air changes

per hour. In addition to Aspen bedding substrate, standard environmental enrichment of 1-2 nestlets, a cardboard tunnel and, with HFD, three wooden chew blocks were provided.

The care and use of mice in the study was carried out in accordance with UK Home Office regulations, UK Animals (Scientific Procedures) Act of 1986 under 3 UK Home Office licenses that approved this work, which were reviewed regularly by the WTSI Animal Welfare and Ethical Review Body.

Study samples

Most mouse mutants reported here were generated using the “Knockout-first allele” method producing four types of alleles (tm1a, tm1b, tm1c and tm1d)[5]. The strategy relies on the identification of an exon common to all transcript variants, upstream of which a LacZ cassette was inserted to make a constitutive knockout named targeting mutation 1a (tm1a). Unlike the tm1a allele, tm1b creates a frame-shift mutation upon deletion of the selected exon. Tm1c is a functional wild-type allele used to make conditional variants named tm1d. In this study, the vast majority of alleles were constructed based on the tm1a and tm1b strategies (see sections below), with three occurrences of a tm1c allele (for *Fundc1*, *Secisbp2* and *Slc25a21* genes) used in the control set (see main text; **Supplementary Data 2**), and only one occurrence of tm1d (for *Slc25a21* gene).

A subset of mutants were generated using CRISPR/Cas9 methodology, similar to previously reported[6]. Alleles generated by CRISPR/Cas9 are noted by the superscript “em” in **Supplementary Data 1**. Briefly, the critical exon (CE) was deleted using four gRNAs (two gRNAs 5’ and two gRNAs 3’ to the CE region). Cas9 mRNA (Trilink, San Diego, CA) together with the four gRNAs was injected into the cytoplasm

of 1-cell C57BL/6NTac zygotes. Injected embryos were briefly cultured and oviductal embryo transfer performed in 0.5 days post-coital pseudopregnant female recipients (CBA/C57BL/6J). F0 mice were screened for the exon deletion by a combination of end-point PCR and loss of WT allele qPCR. Positive F0 mice were further bred with C57BL/6NTac mice. F1 mice were re-screened by PCR and breakpoints confirmed by Sanger sequencing. A single genotype-confirmed F1 mouse was used to establish the colony used to generate mice for phenotyping. Of note, four mutants were generated using CRISPR/Cas9 to make point mutations, for *Pcdh15*, *Prss53*, *Tgm3*, *Herc1* and *Vrk1* genes (**Supplementary Data 1**).

A set of genes (validation set) was studied multiple times for an additional 120 allelic constructions (see **Supplementary Data 2** for details). In summary, 43 were studied using a different Tm1 allelic construction, for example two mouse models were generated for *Rnf10*, one using a Tm1a construction and the other was a Tm1b (both were associated with major impacts on brain morphology). 29 were studied both at heterozygous and homozygous state, for example the perturbation of *Aff3* at the heterozygous state was not associated with neuroanatomical defects whereas at the homozygous state it had severe defects (corpus callosum dysgenesis and enlarged ventricles). 19 were tested at various age points, for example the inactivation of *Sytl1* altered brain morphology at 16 weeks of age but not at 6 weeks. 7 genes were used as controls to assess drift that could occur over time, for example the knockout of *St18* was studied in 2009 and again in 2013, showing consistent results (no phenotype). 2 genes were studied to control for the experimental procedure (*Arpc1b* and *mir96*), and 1 gene was engineered on a different genetic background (*Cenpj*). The remaining 19 were a mixture of these.

Three datasets representing a total of 6,214 mice were analyzed with the main difference being the age of the mice or the histological orientation of the sectioning. The first consisted of 801 (13%) mice made of 579 mutants and 222-matched WTs at six weeks of age. The second was composed of 4,488 (72%) mice of 3,464 mutants and 1,024-matched WTs, and the third of 925 mice (15%) of 753 mutants and 172-matched WTs, both assessed at sixteen weeks of age. The first and the second datasets were analyzed using the coronal plane[7], while the third dataset the sagittal plane which offers certain benefits for large-scale neuroanatomical screens, as previously discussed[8]. Together, these samples corresponded to 1,566 allelic constructions for 1,446 unique genes. Information related to the construction of the mutant line such as allele, genotype, promoter, cassette, diet and background strain, are provided in **Supplementary Data 5** for each of the datasets.

Brain collections and quality control

The brains were taken from mice on a high-throughput phenotyping project, where a mouse is characterized by a series of standardized and validated set of tests underpinned by standard operating procedures (www.mousephenotype.org/impress), covering a variety of disease-related and biological systems. For mouse management purposes, the cages have both genotype and allele information and hence in-life testing are run unblinded. However, as a high-throughput screen where genes are selected for study without hypothesis, mice are typically studied in multiple batches with multiple genotypes being tested at each occasion, so there is limited room for personal bias to influence the results. The collection of brains was performed blind with prosectors not knowing the genotype of the mouse. Factors thought to affect the variables were standardized as far as possible. Where standardization was not

possible, steps were taken to reduce potential bias. For example, the MGP uses a "minimized operator" defined in the Mouse Experimental Design Ontology as "The process by which steps are taken to minimize the potential differences in the effector by training and monitoring of operator"[9]. The data captured with the MEDO ontology can be accessed at http://www.mousephenotype.org/about-impc/arrive-guidelines. For analysis purposes, the individual mouse was the experimental unit. Mice were housed where possible with typical caging density of 3-5 mice per cage.

The Mouse GP and MGP Select phenotyping pipelines were identical (dysmorphology, grip strength, ip-GTT, DEXA, x-rays, ABR, eye morphology & necropsy) with the exception that the Mouse GP pipeline had 4 additional screens (hair phenotyping, open field, hot plate & stress induced hypothermia tests). The Brain pipeline, on the other hand, had no testing. All collections were performed during the first part of the light cycle (08:00-11:00am) under random-fed conditions. Cages were randomly processed as part of a larger necropsy collection.

Mouse GP and MGP Select Pipelines: 16 week-old mice were anaesthetized using either Ketamine (100 mg/kg, intraperitoneally) and Xylazine (10 mg/kg, i.p.), or with Avertin (20ml/kg of a 1.25% solution, i.p.) and blood was collected using the retro-orbital route, followed by heart removal for confirmation of death. The brains were then dissected out and drop fixed in 10% neutral buffered formalin.

Brain Pipeline: Perfusions were performed under terminal anesthesia on 6 weeks old mice. Brains were perfused *in situ* using 4% paraformaldehyde (PFA, pH 8) before the brain was collected and further fixed by immersion in cold 4% PFA for either 4 hours or 48 hours. Finally, brains were transferred to a 30% sucrose-phosphate buffered saline (PBS) solution.

Coronal six-week old dataset

The tm1a allele was the most prevalent (78.9%). 36.4% of the six-week dataset were made of homozygous (HOM), 35.8% of heterozygous (HET) and the remaining were matched WT. The majority had promoter-less constructs (57.5%). The main background strain was B6JTyr;B6N (70%). The subproject name given to brain samples of the six-week old dataset was ("*Sanger_WTCHG_6w*") and processed from May 2008 to October 2010.

Coronal sixteen-week old dataset

Tm1a was also the most prevalent allele (62%) in the sixteen-week old dataset with 18% tm1b. 51% of the samples were HOM, 23% HET, 3.2% hemizygous (HEMI) and the remaining were matched WT. 48.8% had promoter containing cassette. The main background inbred strain was C57BL/6N (84%).

Brain samples belonging to this dataset was structured into four subprojects, reflecting geographical processing of the brain samples and experimental improvements to the protocol. The subproject "Sanger_WTCHG_14-16w" was processed from October 2009 to June 2011 and contained 610 samples (representing 14% of the sixteen-week old samples), "Sanger_WTCHG_ICS" from December 2009 to September 2011 composed of 424 samples (that is 9.5%), "Sanger_ICS" from September 2011 to June 2012 (689 samples that is 15%), and "Sanger_HistologiX" from June 2012 to August 2016 (freeze date of the study). The latter was the largest in size and was composed of 2,765 samples (61.5%). Acronyms such as Sanger, WTCHG, ICS and HistologiX refer to the geographical location (Sanger for Wellcome Trust Sanger Institute, Cambridge, UK; WTCHG for Wellcome Trust Centre for Human

Genetics, Oxford, UK; ICS for Institute Clinique de la Souris, Illkirch, France; and HistologiX for a service provider in Nottingham, UK).

Sagittal sixteen-week old dataset

In this most recent dataset, the em allele was the most common (51.8%), followed by tm1b (23.5%) and tm1a (11.2%). 60% were made of homozygous (HOM), 18% of heterozygous (HET), 4% of hemizygous (HEMI) and the remaining were matched WT. The background strain for all the lines belonging to this dataset was B6N (100%). The subproject names given to brain samples were ("*Sanger_CBI_106*") and ("*Sanger_CBI_107*") where CBI stands for the Centre for Integrative Biology, 106 refers to the adult mouse brain at Lateral +0.60mm and 107 at Lateral +0.72mm. These samples were processed from August 9th 2016 to January 31st 2018.

***Fmr1**$^{-/Y}$* **mouse model**

Young adult 8-week-old Fmr1$^{+/y}$ or Fmr1$^{-/y}$ (C57BL/6J x FVB/N) F1 male litter mice were bred at the Mouse Clinical Institute (Illkirch, France) and obtained from crossing female *Fmr1*$^{-/y}$C57BL/6J[10] with male FVB/N to constitute two experimental groups. After weaning, animals were housed with two to three littermates per cage, independently of their genotype, in specific-pathogen-free environment in individually ventilated cages under 12/12 light/dark cycle with temperature-controlled conditions and free access to food and water with hardwood bedding. Fmr1 animal procedures were conducted according to relevant national ethics committee (Comité National de Réflexion Ethique en Expérimentation Animale) and international guidelines (86/609/CEE).

Data collection

Supplementary Figure 1 shows the overall experimental workflow. All standard operating procedures are described in more details elsewhere[7,8]. In general, brain samples were immersion-fixed in 10% formalin for at least 48 hours, before paraffin embedding and sectioning at 5µm thickness. Three coronal and one sagittal sections were stereostatically defined and named as *critical* sections (**Supplementary Fig. 1**). These *critical* sections were double-stained (Luxol Fast Blue for myelin and Cresyl violet for neurons) and scanned at cell-level resolution using the Nanozoomer whole-slide scanner 2.0HT C9600 series (Hamamatsu Photonics, Shizuoka, Japan). A total of 85 co-variates, for example sample processing dates and usernames were collected at every step of the procedure (**Supplementary Data 3**), as well as 118 brain morphological parameters of 77 area and 39 length measurements, and the number of cerebellar folia (**Supplementary Data 4** and **Supplementary Fig. 2**). All samples were also systematically assessed for cellular ectopia (misplaced neurons). Of note, amongst all the mouse mutant lines assessed, we found three occurrences of ectopia specifically in the hippocampus (*Eml1*$^{-/-}$, **Fig. 2a**; Rnf10$^{-/-}$ and Dcx$^{-/Y}$) and one case of cortical convolutions (*Lamc3*$^{-/-}$).

Development of a relational database

We designed an in-house relational database using the FileMaker (FM) Pro database system for easy access from different geographical sites and better data management. Every aspect of the procedure is managed through this database, for example generating image scan names and directory architecture for 20TB of image data, image quality control, measurements and statistical pipelines using FM build in

scripting capabilities and an interface with R (version 3.4.0, https://www.r-project.org/) and ImageJ (Fiji, version 1.51e) scripts. Within this database, co-variate and measurement data for more than 10,000 brain images were collected and QCed entirely blind to the genotype. The general design of the database is shown in **Supplementary Figure 3** and consists of nine main tables each containing a unique primary key (explained below).

Database tables

1) The "*Mice*" table relates to a unique mouse sample identified using a barcode that starts with the letter "M" for "Mouse" followed by 8 digits, for example "M02386649". The information that is associated to each unique barcode includes sex, genotype, genetic background strain, allele and the subproject identification.

2) The "*Batch*" table incorporates batch information (defined as a group of brains received and processed together) with quality control emanating from both the "*Quality control*" and "*Measures*" tables in order to assess, on a per batch basis, the evolution of data acquisition and section quality. **Supplementary Figure 3** is a screenshot of an automatically generated graph allowing for quality control of the critical section. Batch-related information, such as the name of the experimenter or equipment, is then easily accessed if substandard sectioning quality is noticed and needs troubleshooting.

3) The "*Histology*" table incorporates general information about sectioning, staining and storage of individual paraffin blocks and slides. The original mouse barcode is preceded by a code corresponding to each critical section.

4) The "*Brain scan*" table lists all images scanned and slides pending scanning with relevant information such as date of scan, users and server location of the images.

5) The "*Users*" table lists all users and usernames, and lists access privileges.

6) The "*Quality control*" table contains quality control information for each analyzed image. When the user analyses an image, they call a FileMaker Pro script, which interacts with an ImageJ Macro and the table "*Measures*". The user is prompted to score the picture, assess if the section is critical and symmetrical, the results of which are stored in the "*Quality Control*" table**.** Analysis is performed through a series of ImageJ prompts for each parameter, asking whether the parameter is analyzable and if not, giving the user a list of reasons to choose from (NC, not critical; NS, not symmetrical; NM, not measurable; NV, not visible; NA, not applicable). The measure name, the numerical measure, or if not available, the reason for failing the measure, are then imported in three separate fields of the "*Measures*" table. Regions Of Interests (ROI) coordinates are also saved during the ImageJ Macro within the image repositories. This offers the possibility for FM to call images and associated ROIs at any moment using on screen buttons.

7) The "*Measures*" table is designed to hold the name of a measurement in a single field and a second field containing the value associated to it or, as stated above, a third field containing a reason for not taking the measurement instead. Hence, the primary key corresponds to a specific brain and a specific measure. This design allows flexibility and the implementation of new measurements without affecting the structure of the database itself. After analysis, the database checks the number of imported measurements and the original image is replaced by the rotated image on which the analysis has been performed.

8) The "*Outliers*" table stores data flagged as extreme measures. The data is mostly accessed via the "Measures" table using relationships, where data points are grouped in sub layouts and are analyzed for outliers or compared to corresponding WT datasets. FM has limited statistical capability (mean, standard deviation, min, max) but

scripting within FM was used to automatically identify outliers based on a 1.2 interquartile rule when measurements are grouped by a set of search criteria (**Supplementary Figure 3**). An interlinked series of FM and ImageJ scripts allows for these outliers to be either validated or changed in the database and the eventual changes made to the corresponding ROIs are updated accordingly. Student's t-tests were also implemented (**Supplementary Figure 3**) offering a quick check option but a FM script was used to run the statistical test in R and retrieve the output associated with the current record selection. T-tests were typically done for a given gene, genotype, sex and measure against matched WTs the results of which are listed in graphs showing individual data points.

9) The "*Genes*" table contains curated information about gene function and is accessed via a web portal on FileMaker Pro. It queries UniProt and OMIM databases to extract human orthologous gene, human diseases, and disease subcategories.

Integration with database environment

The database was designed to interact with its environment at multiple levels.

1) In order to speed up scanning and avoid human errors in data entry during brain section scanning, the creation of worklists, folder repositories and filenames is entirely scripted using the FM build in language and command line functions.

2) Once scanned, electronic images of brain sections are sent to the repository and an ImageJ Macro is used to automatically resize (if images are >1.5 Gb), rotate and crop the images. An unmodified duplicate of the picture is kept until quality control and analysis is completed.

3) Images are analyzed using a combination of FM and ImageJ scripts (see above "table 6"). As the image analysis is done blind of genotype information, a "deblind request" is subsequently send to the Wellcome Trust Sanger Institute (once

data collection and quality control is complete) and the genotypes updated in our database under the "*Mice*" table.

4) Student's t-tests are computed in R using FM scripting and output is retrieved by FM Pro database.

5) Data export is scripted to allow for the concatenation of data subsets from almost every table of the database and the creation of a formatted dataset directly usable for linear mixed model analysis using the R software through an in-house statistical pipeline.

Image quality control

High quality brain images were routinely assessed to keep track of potential drifts and recorded within the relational database as described above. Each image was independently assessed and based on four criteria: 1) suitability for analysis, 2) adequacy of the intensity and contrast of the staining to properly delineate brain structures, 3) sectioning precision and 4) symmetry.

Damaged sections

The quality of the brain images depends mostly on the quality of the dissection. Structures at the extremities of the brain such as the cerebellum are typically more prone to damage when compared to the rest of the brain. As a consequence, critical section 3 (cerebellum) was the most vulnerable to distortions and only 2,177 images (41%) of section 3 could be analyzed, by contrast to 5,086 images of section 2 (96%) and 3,869 of section 1 (73%). Critical section 4 gave maximum recovery with 925 images being analyzed (100%).

Staining quality

Because we used a standardized procedure, staining of only very few images (n=20) were too dark or too light preventing sufficient contrast to visually distinguish brain regions.

Sectioning precision

By contrast to more conventional histo-pathological screens that often rely on qualitative assessment, we used a quantitative approach where each section had to pass well defined stereotaxic coordinates defined accordingly to the Mouse Brain Atlas[11] before image analysis. To do this, we recorded how close the image to be analyzed was to the critical section (that is at the precise stereotaxic position) (**Supplementary Fig. 5**). On average, 69.4% of all images were exactly critical: 66% for section 1, 73.7% for section 2, 59.3% for section 3 and 78.7% for section 4. If the images were not critical, the position in µm relative to the Atlas[11] was recorded. 28.3% of section 1 images, 20.6% of section 2, 30.3% of section 3 and 16.2% of section 4 were within an interval of 120µm either posterior or anterior to the critical plane. We evaluated the impact of such interval on the variability of each of the 78 brain parameters, and determined that most parameters did not differ at the exception of the genu of the corpus callosum for section 1, the hippocampus for section 2, the fourth ventricle for section 3 and the caudate putamen for section 4. To minimize unwanted variation, the measurements related to the corpus callosum, the hippocampus, the fourth ventricle and the caudate putamen were thus systematically failed when the section was not at the critical plane.

Symmetry

For coronal sectioning, the symmetry of each image was easily assessed in an effort to control for perfect symmetry between right and left hemispheres as well as between rostral and caudal parts for both coronal and sagittal sectioning (**Supplementary Fig.**

5). 66.3% of section 1 images, 54.2% of section 2, 62% of section 3 and 39.1% of section 4 were perfectly symmetrical. If the images were not perfectly symmetrical, the distance in μm relative to the other hemisphere was recorded. 28% of section 1 images, 37.1% of critical section 2, 26.7% of critical section 3 and 29.2% of critical section 4 images were asymmetric within an interval of 120μm. Sections not symmetrical were failed to limit any experimental variation. 164 samples (3.1%) across the three coronal sections and 5 samples across the sagittal section (0.5%) were failed, either of bad quality, severe asymmetry or away from the critical section.

Data quality control

Data quality being crucial for the interpretation of large-scale projects, a thorough quality control process was designed and implemented at multiple steps of the experimental procedure. A lot of care was given to control human errors and false-positive findings using an in-house quality control pipeline within the FileMaker Pro framework (**Supplementary Fig. 5**). A semi-manual stepwise approach was implemented to standardize and facilitate the cleaning process. All the steps listed below were performed within each subproject.

1) Data was divided into two groups: WTs and mutants. Outliers, typos and erroneous measures were identified using boxplots for each brain parameter measured within the database. Once the data were considered as final, the minimum and the maximum values for each of the 118 brain parameters were recorded for future reference (**Supplementary Data 4**).

2) For each gene and parameter, data distribution versus matched WTs was checked for any errors missed in the previous step using boxplots. For example A, B and C are samples from a mutant line of interest, and sample A has a value of 0.3cm^2

for the total brain area. Sample A will not be flagged in the first step because it will segregate in the normal range when compared to all other mutant samples. However, when compared to samples corresponding to the same gene, for example if sample B has a value of $0.62cm^2$ and C has $0.59cm^2$, A will be flagged.

3) Focusing on mouse NAP genes and their associated WTs, the same experimenter systematically re-measured each parameter. Ambiguities between associated parameters were also systematically checked for any mistakes. For example, when both height and width were taken for a given region but only one gave significant differences, both parameters were re-measured and checked. Once all of these steps were completed, we considered the data as final, and performed a final run of statistical analysis (**Supplementary Fig. 5**), explained in the next section.

QUANTIFICATION AND STATISTICAL METHODS

Statistical framework for gene identification

A total of 6,011 samples were analyzed, corresponding to 1,566 alleles from 1,446 unique genes (164 samples were failed due to poor quality) using scripts in R implemented with the Phenstat package[1].

Assessment of co-variates

A full description of the 85 project's co-variates is provided in **Supplementary Data 3**, composed of:

1) 8 variables providing information about the mouse itself and its husbandry including mouse core strain, promoter status, type of the targeting cassette, diet and phenotyping pipeline underwent by the living animal used by the Wellcome Trust Sanger Institute Mouse Genetics Project (MGP).

2) 55 categorical variables about the histo-morphological procedure, for example the name of the experimenter, equipment, fixative or stain used, and the qualitative assessment of each image such as the symmetry, the quality of the sectioning. One of these variables included the subproject that reflects the geographical localization of the samples.

3) 22 temporal variables, for example necropsy date, the date of the histology or the date of image analysis.

To assess the impact of each co-variate (independent variable) on phenotypic variation (dependent variable), a linear model was fitted and the adjusted multiple-R^2 was calculated. The subproject (reflecting experimental improvements and location of the study) explained the highest phenotypic variance (averaged adjusted multiple-R^2=0.125). Therefore, data were analyzed within each of the subprojects and results merged subsequently. By contrast, the co-variates corresponding to the stereotactic

position of the analyzed brain image and its symmetry score, explained the least variation (average R^2 of 0.0006 and 0.0002, respectively), demonstrating the accuracy of the study. In addition, to account for temporal variation, especially true in large-scale datasets collected over a long period of time[12], the necropsy date of the animal was used as a random variable (batch effect) in the statistical algorithm as it explained most of the temporal variation (averaged adjusted multiple-R^2=0.21).

It is worth mentioning that many technicians have been involved in the study since 2009 and the prerequisite was always good training before starting. To verify this, the same experimenter reanalyzed a set of 26 genes spread throughout the study and their corresponding controls (162 samples), showing highly consistent results[13].

Combining left and right hemispheres

Out of the 78 coronal brain parameters analyzed, 30 were measured on both the left and the right hemispheres including 8 in the striatum, 16 in the hippocampus and 6 in the cerebellum sections. Since our histo-morphological procedure does not allow interhemispheric comparisons for certain (sometimes sections can turn upside down during the histological procedure)[7], we combined measurements of left and right hemispheres, reducing the number of coronal parameters to 48. Of note, the parameter corresponding to the number of folia (3_Folia) was filtered out due to high variability.

Normality tests

To assess whether assumptions for statistical tests were met, the normality of the residuals was calculated for each of the brain parameters in WT (column X "*Gp1 residuals normality test*" **Supplementary Data 7**) and KO (column Z "*Gp2 residuals normality test*" **Supplementary Data 7**). The residuals of the majority of the brain parameters were normally distributed (33 out of 48 coronal parameters and 36 out of 40 sagittal parameters, p-value>0.01). However, when the normality test failed, Q-Q

plots for each non-normally distributed parameter were built instead. This was notably true for parameters pertaining to the ventricles. The comparison of Q-Q plots of normally versus non-normally distributed parameters showed that non-normally distributed parameters did not significantly deviate from the normal distribution. Moreover, the results of non-transformed versus transformed data (the Box-Cox transformation) were compared and no significant difference was found. Hence, we proceeded in the statistical tests without data transformation for all coronal and sagittal parameters.

Linear mixed model and data processing

To account for temporal variation that may have occurred since the start of the study in 2009, we used a linear mixed model framework computing the necropsy date of the animal as a random variable. The model was fitted in R using a parallelized version of PhenStat (version 2.2.4), a package developed for statistical analysis of large-scale phenotypic data from the International Mouse Phenotyping Consortium[1], available at https://www.bioconductor.org/packages/devel/bioc/html/PhenStat.html.

The parallelization was essential to improve processing speed.

Phenstat is described as a top-down methodology involving 6 steps:

- Fitting a loaded model
- Test for batch effect
- Test of covariance structure
- Reduction of model by removing non-significant effects
- Testing the explanatory variable (genotype effect)
- Model diagnosis

Several models incorporating co-variates (Equation 2 to 4 below) were tested against batch effect alone (Equation 1) using the Maximum Likelihood Estimate (MLE) method.

The dependent variables were the brain parameters (either the 48 left and right combined for coronal or the 40 sagittal parameters), the explanatory variable was the gene name (fixed variable), the random variable accounting for Batch effect was the necropsy date and co-variates tested were body weight, total brain area and brain weight:

Equation 1: depVariable~Genotype | Batch effect

Equation 2: depVariable~Genotype | Batch effect + TBA

Equation 3: depVariable~Genotype | Batch effect + Body Weight

Equation 4: depVariable~Genotype | Batch effect + Brain weight

Phenstat excludes factors when the effect of the covariance structure is not significant. A significant number of parameters and genes were thus analyzed using Equation 1 regardless of the model fed into Phenstat. When covariance structure has an impact on the dependent variableequations 2 to 4 were used instead and the maximum likelihood was plotted against Equation 1, pairwise for each gene and phenotype combination (**Supplementary Fig. 5**). In all cases, were covariance structure had an effect, the fitness of Equation 1 yielded higher MLE than Equation 2 to Equation 4, justifying the use of a model with only the random variable.

The variances were computed as equal. When the model failed to fit due to a lack of variation between samples for any of the brain parameters, jitter noise was added randomly at 1000th of the signal difference for the parameter of interest, as recommended by PhenStat[1]. To enable comparisons within equivalent groups, only samples with C57BL/6 background and similar age (12-17 weeks old) were used in the linear mixed model within each of the subprojects (**Supplementary Fig. 5**). In addition, 39 samples defined as singletons, when only one sample was available for any given mutant gene, were failed and not processed any further.

Gene association was considered as significant when the adjusted p-value was below 0.1 (see section below). The quantile-quantile plots (Q-Q plots) of observed p-values for each of the subprojects versus the expected null distribution provided further support for the adequacy of this threshold, because the most significant deviation of adjusted p-values from the expected distribution was at the threshold below 0.1. The percentage change of affected structures was also calculated as following:

Genotype % change = ((average mutant – average WT)/average WT)*100.

Supplementary Data 10 provides association and percentage change data for 1,566 assessed alleles across 48 left and right combined for coronal and 40 for sagittal parameters. Relevant Mammalian Phenotype (MP) terms were used to describe neuroanatomical defects and when needed, new MP terms were created specifically for this project through collaboration with the Jackson Laboratory (http://www.informatics.jax.org/) (**Supplementary Data 8**).

Student's t-test

For a small subset of data (about 2%) representing 34 genes (**Supplementary Data 6**), that did not pass the filters due to different age and/or background, a two-tailed distribution Student's t-test with equal variances was fitted instead of the linear mixed model. The mutant samples were analyzed using their colony controls (the same mouse line).

Multiple testing corrections and permutations

Multiple testing corrections were performed using the Benjamini-Hochberg (BH) method. From this, five gene lists were generated based on 1%, 5%, 10%, 15% and 20% false discovery rate (FDR) (see column C in **Supplementary Data 10**). To control for the false discovery rate, a set of 100 permutations was run within each subproject.

The average number of significant genes in a set of 100 permutations was compared to the results of the true data.

Data imputation and imputed datasets

A newly developed highly performing imputation tool (PHENIX version 1.0[14]), which is based on a Bayesian multiple-phenotype mixed model method, was used to overcome the problem of missing data in downstream analyses that relied on full datasets. Because imputation best boosts signals when the missing data rate is less than 10%[14], imputation was computed on section 1 (Bregma +0.98mm), section 2 (Bregma -1.34mm), and section 4 (Lateral 0.60mm) parameters of the sixteen-week old dataset (39 brain parameters for coronal and 40 for sagittal) but not section 3 that had a higher rate of missing data (**Supplementary Data 11**). More specifically, the imputation was performed within each subproject using samples with C57BL/6 background and at similar age (12-17 weeks old). Two matrices were used: one constituting the phenotypic data and a positive kinship matrix describing the genetic covariance. The kinship matrix was created assuming 1-to-1 relatedness between the mice within each subproject. This resulted in a new imputed dataset made of 1,380 alleles from 1,306 unique genes, totaling 5,281 mouse samples (**Supplementary Data 12**). The same statistical pipeline as the one applied to non-imputed data was used (**Supplementary Data 13**), producing a new gene-association list (**Supplementary Data 14**).

To assess the quality of the imputation, we compared the overlap between NAP genes identified through imputed versus non-imputed datasets (**Supplementary Fig. 6**). The overlap between imputed versus non-imputed data for gene identification was 84% with the imputed data resulting in 30 additional genes, whereas the non-imputed data had 32 genes that did not overlap with the imputed results. We manually verified

each of the additional 30 genes potentiated by imputation by checking the raw data for any associated brain parameter. 27 genes were literally borderline significant in the non-imputed dataset (just below the threshold of BH-p<0.1) suggesting that these genes are likely to be true positives. Two genes (*Kifap3* and *Ren1*) had missing data for their corresponding affected parameter in the non-imputed data, and one gene (*Tsks*) was not significant in the non-imputed data. Amongst the 32 genes that did not overlap with the imputed results, 16 did not undergo imputation for various reasons such as younger age or different genetic background (see **Supplementary Fig. 6**), 13 were borderline significant in the imputed dataset and 3 genes (*Anp32e*, *Raph1* and *Tmem127*) not significant in the imputed data.

We complemented this analysis by comparing the overlap between affected parameters in imputed versus non-imputed data, and found an overlap of 91% (**Supplementary Fig. 6**). In a similar way to NAP genes, the majority of the newly identified brain parameters through imputation (from a total of 51) were borderline significant in the non-imputed data. In addition, the distribution of the brain parameters within the WT group in imputed versus non-imputed data was checked. The imputation did not affect the distribution of the brain parameters and occurred within the expected range (an example is provided in **Supplementary Fig. 6**).

Both the number of genes detected and the hit rate were consistent between imputed versus non-imputed gene lists, acting as a proof that imputation is not distorting the genetic data, but unanimously increasing detection power. All downstream analyses were thus performed on the imputed dataset unless otherwise stated.

Inter-regional brain relations

Robust Pearson correlations

Relations between the 39 imputed coronal brain parameters were calculated within each subproject for WT samples (n=991), mutant samples associated to neuroanatomical defects (n=158 genes that corresponds to 487 samples; BH-p<0.1) and mutant samples not associated to neuroanatomical defects (n=411 genes that corresponds to 1,247 samples, BH-p>0.5). The minimum number of data points per pairwise correlation was set to 25. For normalization purposes, the square root of area measurements was calculated to be comparable with length measurements. To reduce the impact of noise, the Stahel-Donoho estimator was calculated as a robust correlation estimator (an example of robust estimator would be the median, which is the robust equivalent of the mean) using "*robust*" (R package version 3.2.3). Pairwise correlations were then performed using the Pearson method within each subproject. The Olkin-Pratt estimator was applied to combine all correlations between the subprojects[15]. The top one hundred correlations with r^2 coefficient higher than 0.3 were selected for further analysis. A chord diagram was used for the visualization of correlation analyses (**Supplementary Fig. 4**). We found brain morphological parameters either pertaining to the same region (for example, the height and area of the retrosplenial cortex), or at close proximity or directly adjacent in the topographic map (for example, the primary motor and cingulate cortices) to be positively correlated in WTs.

t-Distributed Stochastic Neighbor Embedding

t-Distributed Stochastic Neighbor Embedding (https://github.com/jkrijthe/Rtsne) or t-SNE, a dimensionality reduction technique, was used as an alternative way to study

the relations between brain parameters. This approach allowed us to build a 2D-map of the 39 imputed coronal brain parameters based on their similarities followed by a clustering analysis. Brain parameters were first normalized in both WT samples and samples corresponding to the NAP genes, and a matrix built and center scaled within each subproject. Distances between brain parameters were computed using the Euclidean method. The weighted mean was calculated to combine distances between brain parameters. Using Rtsne (R package version 3.2.3), t-SNE was applied, projecting our dataset into 2 dimensions while preserving short distances and accepting a distortion of long distances. The clusters were found using single-linkage clustering (**Supplementary Fig. 4**).

Gene lists enrichment

Gene lists (and associated references) used in this study are summarized in **Supplementary Data 19**.

Mouse to human orthology

Homology information regarding 18,926 human and 20,015 mouse genes was downloaded from Ensembl Biomart[16]. Considering 16,377 human-mouse gene pairs orthologous at a 1:1 ratio, we identified unique human orthologues for 960 mouse mutant genes.

Functional gene lists

We downloaded a set of 410 genes associated with mouse embryonic lethality (Dickinson et al., 2016). By mining the literature, we downloaded 152 and 1,080 mouse synaptosome and postsynaptic density genes, respectively (*Genes2Cognition* database)[17]. We also retrieved a list of 842 mouse FMRP target genes and their human orthologues (n=842) from [18]. Moreover, we downloaded a set of 1,223 human brain-

specific genes from the Human Protein Atlas, with at least five-fold higher mRNA levels in the cerebral cortex compared to the average expression across all 32 tissues analyzed [19].

Gene haploinsufficiency (HIS) scores

We downloaded probabilities of HIS for 11,448 protein-coding genes with Ensembl Ids, from [20]. Probabilities were estimated using various gene features including genomic (e.g. gene length), functional (expression patterns) and evolutionary properties[20].

Gene intolerance scores

Mutational constraint scores were downloaded for 17,047 and 17,816 protein-coding genes from [21] and [22], respectively. These scores are entitled residual variation intolerance score (RVIS) and probability of being loss of function intolerant (pLI), respectively. RVIS evaluates the rate of common functional gene variation using data from the National Heart, Lung and Blood Institute (NHLBI) GO Exome Sequencing Project, such that genes with lower RVIS are more likely to be under purifying selection and thus intolerant to mutations[21]. Conversely, the pLI score assesses the depletion of penetrant loss of function rare gene variants, defined as nonsense, splice-site and frameshift mutations, based on data from the Exome Aggregation Consortium (ExAC; [22]).

Moreover, selection coefficients (s_{het}) (measure of the reduction in fitness associated with the loss of heterozygosity across gene alleles) for 15,999 human genes were downloaded from [23]. These scores have been estimated based on the expected gene mutation rates, number of observed heterozygous protein-truncating variants in individuals from ExAC and the number of chromosomes sampled[23]. Finally, we downloaded the rate of synonymous (dS) and non-synonymous (dN) gene

substitutions between mouse and human for 23,147 mouse genes from Ensembl Biomart[16] and quantified selection pressures as the dN/dS ratio.

High confidence disease gene lists

We downloaded a list of 6,116 disease genes annotated in the OMIM database from Ensembl Biomart (MIM Morbid list)[16]. Additionally, we extracted three non-exclusive lists of ID genes, consisting of 292, 429, 603 and 959 genes from [24], [25], [26] and [2], respectively.

Genes and phenotypes from patients with neurodevelopmental disorders

We downloaded a list of 421 genes disrupted by *de novo* nonsense mutations in 2,508 ASD patients from the Simons Simplex Collection (SSC), for who extensive clinical data – including the presence of physical and morphological abnormalities – is available[27].

We retrieved CNV data for patients presenting with developmental delay, ID and/or congenital abnormalities. We only considered the 260 patients *de novo* CNVs of sizes <5mb[28,29]. Patients were systematically phenotyped using terms from the Human Phenotype Ontology[30,31].

As whole-exome sequencing studies of ID involve relatively small cohorts (< 200 patients), we considered *de novo* missense, nonsense, splice-site and frameshift variants. We retrieved 58 and 76 genes affected by such mutations in patients with severe and moderate/severe ID ([32] and [33], respectively).

Gene ontology (GO) annotations

We downloaded GO biological process (GO-BP), molecular function (GO-MF) and cellular compartment (GO-CC) annotations for 18,469 mouse and 20,165 human genes from the GO database[34]. To reduce uninformative results, we considered GO

terms annotated with >100 and <4,000 genes, resulting in 1,659 and 1,761 GO pathways annotated with 17,968 mouse and 19,435 human genes, respectively.

Statistical tests

The excess of genes from particular lists was tested using one-sided right-tailed Fisher's tests. HIS, gene intolerance scores and phenotypic similarities were compared between two sets using one-sided right-tailed Mann-Whitney U tests. Comparisons were made between values for the top 10% NAP *versus* bottom 10% NAP; lethal *versus* bottom 10% NAP and permutations *versus* bottom 10% NAP (see set descriptions above). While differences were observed between the features of the top 10% and bottom 10% NAP genes, nothing was observed when compared to the permuted gene sets (**Figure 3**). The correlation between gene deleteriousness measures and strength of neuroanatomical abnormalities was assessed using linear regression. The strength of neuroanatomical abnormalities was defined as the maximum absolute z-score deviation from wildtype mice across all features examined. The over-representation of GO terms was assessed using one-sided right-tailed hypergeometric tests, and a BH correction for multiple testing was applied to control for the number of terms tested in each category (i.e. 1,188 mouse GO-BP, 265 mouse GO-MF and 206 mouse GO-CC terms; and 1,288, 265 and 208 human terms, respectively). The over-representation of mouse whole body traits downloaded from MGI was assessed using one-side right-tailed Fisher's test. BH correction for multiple testing was applied to control for the number of categories tested (i.e. 26).

Gene expression datasets

Gene expression datasets (and associated references) used in this study are summarized in **Supplementary Data 20**. We downloaded mouse RNA sequencing

data, generated by the Encode project[35,36]. Focusing on 21,680 protein-coding genes, we formed a mouse gene expression datasets by calculating the mean FPKM value for each gene and tissue or developmental point, across the relevant replicates. Specifically, we constructed a brain developmental dataset by considering gene expression levels in the CNS at embryonic stages E11.5, E14 and E18 and in the adult brain. As transcriptomic data were not available for the adult CNS, we considered the average expression level across cerebellum, cortical plate and frontal cortex tissues collected from eight-week-old mice. Given the discrepancy in temporal brain regions considered, observations made using this dataset require further validation.

We downloaded RNA sequencing gene expression data for 16,611 protein-coding human genes across 51 bodily tissues from the GTEx project[37]. For each gene, tissue expression was calculated as the mean FPKM value across all samples mapping to a given tissue (average of 83.70 samples per gene). The 51 tissues were assembled into 30 structures, by considering the average gene expression levels across relevant tissues (**Supplementary Data 20**).

We downloaded normalized RNA sequencing expression data for 19,672 protein-coding human genes across 27 brain tissues spanning twelve developmental stages from Brainspan[38]. To evaluate temporal brain expression patterns, we defined six developmental stages, as suggested by the experimental protocols of Brainspan (**Supplementary Data 20-21**). For each gene, we calculated the overall brain expression level at each developmental point as the median RPKM value across all tissues and samples mapping to the given stage. To evaluate spatial brain expression patterns, we assembled the 27 brain tissues into six brain regions (**Supplementary Data 20**). For each gene, the brain region expression level was defined as the median RPKM value across all samples mapping to the given region, independently of the

sample's time point during development. By calculating the median RPKM value across gene samples mapping to all combinations of brain regions and developmental points, we identified 13,306 brain-expressed genes, with RPKM values >1 in at least one brain region during one developmental stage (**Supplementary Data 19**). Expression datasets were normalized, such that expression levels were scaled between 0 and 1 for each body structure, brain region or developmental stage. The expression of each gene was then normalized across tissues, regions or stages, using Euclidean distances, to provide specificity.

Gene expression specificity was examined using a permutation-based approach, wherein the cumulative expression level of genes from a set of interest was compared to that of 1,000 permuted gene sets, formed by randomly sampling the same number of genes from the genome, matched for CDS. Empirical p-values were derived, reflecting the fraction of permutations, during which randomized sets have a higher cumulative expression level than the set of interest. BH corrections for multiple testing were applied to control for the number of tissues, regions or stages tested (n=4 for developmental CNS expression dataset, n=30 for GTEx and n=6 for Brainspan datasets).

Gene expression levels in the neocortex, amygdala, hippocampus and striatum, as given by the Brainspan regional dataset, were normalized between 0 and 1 against the mean RPKM value of all genome genes in the corresponding region. We derived each gene's expression level in critical section 1 (Bregma +0.98mm) and section 2 (Bregma -1.34mm) as the maximum expression value across the striatum and neocortex or across the amygdala, hippocampus and neocortex, respectively. For each module, we calculated the mean of the expression ratio between Bregma

sections 1 and 2 across the corresponding genes. Notably, the mean expression level across Bregma section-associated tissues generates comparable results (not shown).

Gene networks

The mouse and human gene networks downloaded or constructed (and associated references) are summarized in **Supplementary Data 21**. We downloaded MouseNet v2, a functional mouse gene network incorporating various mouse -omics resources (i.e PPI, gene expression data, functional annotations and homology data)[39].

We constructed human body-wide and brain spatiotemporal gene co-expression networks, using GTEx[37] and Brainspan[38] RNA sequencing data across 51 bodily tissues and across 27 brain tissues spanning twelve developmental, respectively. Genes with FPKM or RPKM values <1 in >95% of samples were excluded and gene co-expression networks were derived by estimating the correlation of expression patterns of pairs of protein-coding gene across all associated samples, using Pearson's coefficients[40]. Human PPI networks were downloaded from String[41]. Lastly, by combining various human genomic datasets (i.e. PPIs, GO, MGI, KEGG, Reactome, and gene expression data), we constructed an integrated gene network, termed phenotypic linkage network (PLN), wherein pairs of gene are assigned scores, reflecting their likelihood of functional interaction, based on multiple lines of evidence[42].

Network clustering

To examine the network interconnectedness of a set of genes, we compared the sum of their network links to that of permuted gene sets, constructed by randomly sampling the same number of human genes, matched for CDS and network connectivity[42]. By running 1,000 permutations, we derived an empirical p-value, reflecting the fraction of

randomized gene sets, which are more interconnected in the evaluated network, than the set of interest.

Module identification

We identified modules of strongly interconnected genes by partitioning gene networks using the Louvain algorithm[43]. This greedy optimization method attempts to maximize the modularity of a network division (strength of connections inside modules as compared to between modules). The algorithm was implemented in Gephi using a resolution parameter of 1.

SUMMARY OF KEY RESOURCES TABLE

REAGENT or RESOURCE	SOURCE	IDENTIFIER
Biological Samples		
Brain samples of mutant mouse lines derived from Sanger Mouse Genetics Project (Hinxton, UK)	IMPC, https://www.mousephenotype.org/	**Supplementary Data 1**
Chemicals, Peptides, and Recombinant Proteins		
Cresyl violet acetate	Sigma-Aldrich	Cat#C5042-106
Solvent Blue 38	Sigma-Aldrich	Cat#S3382-25G
Lithium carbonate	Sigma-Aldrich	Cat#13010-100G-R
Oxalic acid	Sigma-Aldrich	Cat#241172-50G
Deposited Data		
Assessed brain parameters and co-variates	This paper	**Supplementary Data 3,4**
Non-imputed data and their analysis	This paper	**Supplementary Data 5,7,10**
Imputed data and their analysis	This paper	**Supplementary Data 12-14**
Comprehensive atlas of neuroanatomical defects	This paper	**Supplementary Data 9**
Annotated brain images	This paper	https://www.mousephenotype.org/
Experimental Models: Organisms/Strains		
Mouse: *Fmr1*$^{+/y}$ and *Fmr1*$^{-/y}$ (C57BL/6J x FVB/N)	Mientjes et al., 2006	N/A
Software and Algorithms		
FileMakerPro version 14.0.6	FileMaker Inc.	https://www.filemaker.com/
ImageJ/Fiji version 1.51e	*Schneider* et al., 2012	http://imagej.nih.gov/ij/
R version 3.4.0	R Core Team	https://www.r-project.org/
PhenStat version 2.2.4	Kurbatova et al., 2016	https://www.bioconductor.org/packages/devel/bioc/html/PhenStat.html
Phenix version 1.0	Dahl et al., 2016	https://mathgen.stats.ox.ac.uk/genetics_software/phenix/phenix.html
Robust: Port of the S+ "Robust Library"	Maintainer: K. Konis	https://cran.r-project.org/web/packages/robust/index.html
Rtsne: T-Distributed Stochastic Neighbor Embedding	Maintainer: J. Krijthe	https://cran.r-project.org/web/packages/Rtsne/index.html
Integrative R statistical pipeline for the identification of genes	This paper	available upon request
R Script for production of a color-coded heat map of neuroanatomical defects	This paper	available upon request
R script for inter-regional brain relations	This paper	available upon request

R script to build a 2D-map of brain parameters	This paper	available upon request
Other		
Public gene lists		**Supplementary Data 19**
Expression datasets		**Supplementary Data 20**
Gene networks		**Supplementary Data 21**
Mouse whole body phenotypes	The Jackson Laboratory	http://www.informatics.jax.org/downloads/reports/index.html#pheno

SUPPLEMENTARY REFERENCES

1 Kurbatova, N., Mason, J. C., Morgan, H., Meehan, T. F. & Karp, N. A. PhenStat: A Tool Kit for Standardized Analysis of High Throughput Phenotypic Data. *PLoS One* **10**, e0131274, doi:10.1371/journal.pone.0131274 (2015).

2 Kochinke, K. *et al.* Systematic Phenomics Analysis Deconvolutes Genes Mutated in Intellectual Disability into Biologically Coherent Modules. *Am J Hum Genet* **98**, 149-164, doi:10.1016/j.ajhg.2015.11.024 (2016).

3 Lalani, S. R. & Belmont, J. W. Genetic basis of congenital cardiovascular malformations. *Eur J Med Genet* **57**, 402-413, doi:10.1016/j.ejmg.2014.04.010 (2014).

4 White, J. K. *et al.* Genome-wide generation and systematic phenotyping of knockout mice reveals new roles for many genes. *Cell* **154**, 452-464, doi:10.1016/j.cell.2013.06.022 (2013).

5 Skarnes, W. C. *et al.* A conditional knockout resource for the genome-wide study of mouse gene function. *Nature* **474**, 337-342, doi:10.1038/nature10163 (2011).

6 Boroviak, K., Doe, B., Banerjee, R., Yang, F. & Bradley, A. Chromosome engineering in zygotes with CRISPR/Cas9. *Genesis* **54**, 78-85, doi:10.1002/dvg.22915 (2016).

7 Mikhaleva, A., Kannan, M., Wagner, C. & Yalcin, B. Histomorphological Phenotyping of the Adult Mouse Brain. *Curr Protoc Mouse Biol* **6**, 307-332, doi:10.1002/cpmo.12 (2016).

8 Collins, S. C. *et al.* A Method for Parasagittal Sectioning for Neuroanatomical Quantification of Brain Structures in the Adult Mouse. *Curr Protoc Mouse Biol* **8**, e48, doi:10.1002/cpmo.48 (2018).

9 Karp, N. A. *et al.* Applying the ARRIVE Guidelines to an In Vivo Database. *PLoS Biol* **13**, e1002151, doi:10.1371/journal.pbio.1002151 (2015).

10 Mientjes, E. J. *et al.* The generation of a conditional Fmr1 knock out mouse model to study Fmrp function in vivo. *Neurobiol Dis* **21**, 549-555, doi:10.1016/j.nbd.2005.08.019 (2006).

11 Paxinos, G. a. F., K.B.J. *The Mouse Brain in Stereotaxic Coordinates*. 3rd ed. edn, (Academic Press, San Diego, 2007).

12 Karp, N. A., Melvin, D., Sanger Mouse Genetics, P. & Mott, R. F. Robust and sensitive analysis of mouse knockout phenotypes. *PLoS One* **7**, e52410, doi:10.1371/journal.pone.0052410 (2012).

13 Kannan, M. *et al.* WD40-repeat 47, a microtubule-associated protein, is essential for brain development and autophagy. *Proc Natl Acad Sci U S A* **114**, E9308-E9317, doi:10.1073/pnas.1713625114 (2017).

14 Dahl, A. *et al.* A multiple-phenotype imputation method for genetic studies. *Nat Genet* **48**, 466-472, doi:10.1038/ng.3513 (2016).

15 Alexander, R. A. A note on averaging correlations. *Bulletin of the Psychonomic Society* **28**, 335-336 (1990).

16 Cunningham, F. *et al.* Ensembl 2015. *Nucleic Acids Res* **43**, D662-669, doi:10.1093/nar/gku1010 (2015).

17 Croning, M. D., Marshall, M. C., McLaren, P., Armstrong, J. D. & Grant, S. G. G2Cdb: the Genes to Cognition database. *Nucleic Acids Res* **37**, D846-851, doi:10.1093/nar/gkn700 (2009).

18 Darnell, J. C. *et al.* FMRP stalls ribosomal translocation on mRNAs linked to synaptic function and autism. *Cell* **146**, 247-261, doi:10.1016/j.cell.2011.06.013 (2011).
19 Uhlen, M. *et al.* Proteomics. Tissue-based map of the human proteome. *Science* **347**, 1260419, doi:10.1126/science.1260419 (2015).
20 Huang, N., Lee, I., Marcotte, E. M. & Hurles, M. E. Characterising and predicting haploinsufficiency in the human genome. *PLoS Genet* **6**, e1001154, doi:10.1371/journal.pgen.1001154 (2010).
21 Petrovski, S., Wang, Q., Heinzen, E. L., Allen, A. S. & Goldstein, D. B. Genic intolerance to functional variation and the interpretation of personal genomes. *PLoS Genet* **9**, e1003709, doi:10.1371/journal.pgen.1003709 (2013).
22 Lek, M. *et al.* Analysis of protein-coding genetic variation in 60,706 humans. *Nature* **536**, 285-291, doi:10.1038/nature19057 (2016).
23 Cassa, C. A. *et al.* Estimating the selective effects of heterozygous protein-truncating variants from human exome data. *Nat Genet* **49**, 806-810, doi:10.1038/ng.3831 (2017).
24 Pinto, D. *et al.* Convergence of genes and cellular pathways dysregulated in autism spectrum disorders. *Am J Hum Genet* **94**, 677-694, doi:10.1016/j.ajhg.2014.03.018 (2014).
25 Casanova, E. L., Sharp, J. L., Chakraborty, H., Sumi, N. S. & Casanova, M. F. Genes with high penetrance for syndromic and non-syndromic autism typically function within the nucleus and regulate gene expression. *Mol Autism* **7**, 18, doi:10.1186/s13229-016-0082-z (2016).
26 Mirzaa, G. M., Millen, K. J., Barkovich, A. J., Dobyns, W. B. & Paciorkowski, A. R. The Developmental Brain Disorders Database (DBDB): a curated neurogenetics knowledge base with clinical and research applications. *Am J Med Genet A* **164A**, 1503-1511, doi:10.1002/ajmg.a.36517 (2014).
27 Iossifov, I. *et al.* The contribution of de novo coding mutations to autism spectrum disorder. *Nature* **515**, 216-221, doi:10.1038/nature13908 (2014).
28 Andrews, T. *et al.* Gene networks underlying convergent and pleiotropic phenotypes in a large and systematically-phenotyped cohort with heterogeneous developmental disorders. *PLoS Genet* **11**, e1005012, doi:10.1371/journal.pgen.1005012 (2015).
29 Vulto-van Silfhout, A. T. *et al.* Clinical significance of de novo and inherited copy-number variation. *Hum Mutat* **34**, 1679-1687, doi:10.1002/humu.22442 (2013).
30 Kohler, S. *et al.* The Human Phenotype Ontology in 2017. *Nucleic Acids Res* **45**, D865-D876, doi:10.1093/nar/gkw1039 (2017).
31 Robinson, P. N. & Webber, C. Phenotype ontologies and cross-species analysis for translational research. *PLoS Genet* **10**, e1004268, doi:10.1371/journal.pgen.1004268 (2014).
32 de Ligt, J. *et al.* Diagnostic exome sequencing in persons with severe intellectual disability. *N Engl J Med* **367**, 1921-1929, doi:10.1056/NEJMoa1206524 (2012).
33 Hamdan, F. F. *et al.* De novo mutations in moderate or severe intellectual disability. *PLoS Genet* **10**, e1004772, doi:10.1371/journal.pgen.1004772 (2014).
34 Ashburner, M. *et al.* Gene ontology: tool for the unification of biology. The Gene Ontology Consortium. *Nat Genet* **25**, 25-29, doi:10.1038/75556 (2000).

35 Consortium, E. P. An integrated encyclopedia of DNA elements in the human genome. *Nature* **489**, 57-74, doi:10.1038/nature11247 (2012).

36 Pervouchine, D. D. *et al.* Enhanced transcriptome maps from multiple mouse tissues reveal evolutionary constraint in gene expression. *Nat Commun* **6**, 5903, doi:10.1038/ncomms6903 (2015).

37 Consortium, G. T. The Genotype-Tissue Expression (GTEx) project. *Nat Genet* **45**, 580-585, doi:10.1038/ng.2653 (2013).

38 Sunkin, S. M. *et al.* Allen Brain Atlas: an integrated spatio-temporal portal for exploring the central nervous system. *Nucleic Acids Res* **41**, D996-D1008, doi:10.1093/nar/gks1042 (2013).

39 Kim, E. *et al.* MouseNet v2: a database of gene networks for studying the laboratory mouse and eight other model vertebrates. *Nucleic Acids Res* **44**, D848-854, doi:10.1093/nar/gkv1155 (2016).

40 Steinberg, J. & Webber, C. The roles of FMRP-regulated genes in autism spectrum disorder: single- and multiple-hit genetic etiologies. *Am J Hum Genet* **93**, 825-839, doi:10.1016/j.ajhg.2013.09.013 (2013).

41 Szklarczyk, D. *et al.* STRING v10: protein-protein interaction networks, integrated over the tree of life. *Nucleic Acids Res* **43**, D447-452, doi:10.1093/nar/gku1003 (2015).

42 Honti, F., Meader, S. & Webber, C. Unbiased functional clustering of gene variants with a phenotypic-linkage network. *PLoS Comput Biol* **10**, e1003815, doi:10.1371/journal.pcbi.1003815 (2014).

43 Blondel, V. G., JL.; Lambiotte R. and Lefebvre E. Fast unfolding of communities in large networks. *Journal of Statistical Mechanics: Theory and Experiment* **10**, 10008 (2008).
