# Supplementary material for: Large-scale neuroanatomical study uncovers 198 gene associations in mouse brain morphogenesis
Source: Nat Commun. 2019 Aug 1;10:3465. doi: 10.1038/s41467-019-11431-2 (PMC6671969; doi:10.1038/s41467-019-11431-2)
Supplement: Supplementary file 3 — Reporting Summary [file 41467_2019_11431_MOESM3_ESM.pdf]

nature research

Corresponding author(s): Yalcin, Binnaz

Last updated by author(s): Jul 12, 2019

Reporting Summary

Nature Research wishes to improve the reproducibility of the work that we publish. This form provides structure for consistency and transparency in reporting. For further information on Nature Research policies, see Authors & Referees and the Editorial Policy Checklist.

Statistics

For all statistical analyses, confirm that the following items are present in the figure legend, table legend, main text, or Methods section.

n/a	Confirmed	
☐	☒	The exact sample size (*n*) for each experimental group/condition, given as a discrete number and unit of measurement
☐	☒	A statement on whether measurements were taken from distinct samples or whether the same sample was measured repeatedly
☐	☒	The statistical test(s) used AND whether they are one- or two-sided<br>*Only common tests should be described solely by name; describe more complex techniques in the Methods section.*
☐	☒	A description of all covariates tested
☐	☒	A description of any assumptions or corrections, such as tests of normality and adjustment for multiple comparisons
☐	☒	A full description of the statistical parameters including central tendency (e.g. means) or other basic estimates (e.g. regression coefficient) AND variation (e.g. standard deviation) or associated estimates of uncertainty (e.g. confidence intervals)
☐	☒	For null hypothesis testing, the test statistic (e.g. *F*, *t*, *r*) with confidence intervals, effect sizes, degrees of freedom and *P* value noted<br>*Give P values as exact values whenever suitable.*
☐	☒	For Bayesian analysis, information on the choice of priors and Markov chain Monte Carlo settings
☐	☒	For hierarchical and complex designs, identification of the appropriate level for tests and full reporting of outcomes
☐	☒	Estimates of effect sizes (e.g. Cohen's *d*, Pearson's *r*), indicating how they were calculated

Our web collection on statistics for biologists contains articles on many of the points above.

Software and code

Policy information about availability of computer code

Data collection	Data collection is described in details in the Supplementary Information document. We use FileMakerPro version 14.0.6 (https://www.filemaker.com/) and ImageJ/Fiji version 1.51e (http://imagej.nih.gov/ij/).
Data analysis	Data analysis is described in details in the Supplementary Information document. We use R version 3.4.0 (https://www.r-project.org/), PhenStat version 2.2.4 (https://www.bioconductor.org/packages/devel/bioc/html/PhenStat.html) and Phenix version 1.0 (https://mathgen.stats.ox.ac.uk/genetics_software/phenix/phenix.html).

For manuscripts utilizing custom algorithms or software that are central to the research but not yet described in published literature, software must be made available to editors/reviewers. We strongly encourage code deposition in a community repository (e.g. GitHub). See the Nature Research guidelines for submitting code & software for further information.

Data

Policy information about availability of data

All manuscripts must include a data availability statement. This statement should provide the following information, where applicable:

- Accession codes, unique identifiers, or web links for publicly available datasets
- A list of figures that have associated raw data
- A description of any restrictions on data availability

For data availability: We provide all the data produced in this study within the article and its associated supplementary information and data files. For code availability: All bioinformatics analyses for this study were performed using R. PhenStat and PHENIX packages are available for free download from https://www.bioconductor.org/packages/devel/bioc/html/PhenStat.html and https://mathgen.stats.ox.ac.uk/genetics_software/phenix/phenix.html. In-house R scripts used for the identification of genes are available upon request.

Field-specific reporting

Please select the one below that is the best fit for your research. If you are not sure, read the appropriate sections before making your selection.

☒ Life sciences ☐ Behavioural & social sciences ☐ Ecological, evolutionary & environmental sciences

For a reference copy of the document with all sections, see nature.com/documents/nr-reporting-summary-flat.pdf

Life sciences study design

All studies must disclose on these points even when the disclosure is negative.

Sample size	In inbred mutant mice, we are able to detect neuroanatomical defects with an effect size of 10% or more with 80% detection power using 3 mice per assessed groups (calculated using GPower 3.1). We expect that our stringent criteria for evaluating brain symmetry and positioning will increase sensitivity to detect phenotypes, especially for the hippocampus or the corpus callosum whose size depends largely on these two criteria (see Supplementary Notes for more details).
Data exclusions	We failed 164 samples due to poor image quality (see Supplementary Notes for more details).
Replication	We used two types of replication datasets. First, we engineered 43 mutant lines twice using distinct allelic constructions, showing consistent results. Second, a second experimenter reanalyzed a set of 26 genes and their corresponding controls, showing highly consistent results. More details can be found in the Supplementary Notes.
Randomization	Genes were selected for entry into the pipeline from requests by WTSI faculty and collaborators working on a wide variety of fields, including cancer, metabolism, infection, immunology, behavior and human evolution, but without a priori interest in the data generated from the brain histopathology screen. In addition, genes without prior characterization were assigned by the IMPC to WTSI for phenotyping. Assessed genes were distributed on all chromosomes except Y with no bias on their genomic distribution (see Supplementary Notes for more details).
Blinding	The investigators were all completely blinded to group allocation during data collection, image analysis and first steps of data quality control (details in Supplementary Notes).

Reporting for specific materials, systems and methods

We require information from authors about some types of materials, experimental systems and methods used in many studies. Here, indicate whether each material, system or method listed is relevant to your study. If you are not sure if a list item applies to your research, read the appropriate section before selecting a response.

Materials & experimental systems

n/a	Involved in the study
☒	☐ Antibodies
☒	☐ Eukaryotic cell lines
☒	☐ Palaeontology
☐	☐ Animals and other organisms
☒	☐ Human research participants
☒	☐ Clinical data

Methods

n/a	Involved in the study
☒	☐ ChIP-seq
☒	☐ Flow cytometry
☒	☐ MRI-based neuroimaging

Animals and other organisms

Policy information about studies involving animals; ARRIVE guidelines recommended for reporting animal research

Laboratory animals	16-weeks old male mouse samples were used in this study. Detailed information about genetic background strain, precise age and production is described in the Supplementary Notes.
Wild animals	The study did not involve wild animals.
Field-collected samples	The study did not involve samples collected from the field.
Ethics oversight	The care and use of mice in the Wellcome Sanger Institute study was carried out in accordance with UK Home Office regulations, UK Animals (Scientific Procedures) Act of 1986 under two UK Home Office licences (80/2485 and P77453634) that approved this work, which were reviewed regularly by the Wellcome Sanger Institute Animal Welfare and Ethical Review Body.

Note that full information on the approval of the study protocol must also be provided in the manuscript.
