# Supplementary material for: Large-scale neuroanatomical study uncovers 198 gene associations in mouse brain morphogenesis
Source: Nat Commun. 2019 Aug 1;10:3465. doi: 10.1038/s41467-019-11431-2 (PMC6671969; doi:10.1038/s41467-019-11431-2)
Supplement: Supplementary file 4 — Description of Additional Supplementary Files [file 41467_2019_11431_MOESM4_ESM.docx]

**DESCRIPTION OF ADDITIONAL SUPPLEMENTARY FILES**

File Name: Supplementary Data 1. Description of allelic constructions and their associated unique genes.

Description: Column A gives the most up-to-date gene name (July 2018), column B is the previous gene name, if relevant (November 2017), column C is the description of the gene, column D is the subproject to which the gene belongs to, column E shows the genotype of the mice, followed by allele type (col F), number of mutant mice analyzed (col G), background strain (col H) and age of the mice (col I). Columns J to R provide information on the gene including the Mouse Genome Informatics (MGI) gene identification, genomic features (such as protein-coding, miRNA, lncRNA and chromosomal deletion), chromosome, strand, start and end coordinates (GRCm38.p6), mouse Ensembl gene identifications, human orthologue gene name and human Ensembl gene identification for 1-to-1 human-mouse orthologues (GRCh38.p12, BioMart). Column S indicates the histological procedure (coronal or sagittal). Finally, column T provides references to previous published work documenting similar neuroanatomical defects for NAP genes (BH-p<0.1). NA is not applicable. PM (col F) is point mutation.

File Name: Supplementary Data 2. Replication dataset.

Description: Column A gives the type of the replication study, followed by the full gene name composed of gene_genotype_allele information (col B), the subproject identification (col C), the number of mice per group (col D), the genetic background strain (col E), the age of the mice at necropsy (col F), the adjusted p-value significance threshold (BH-p) based on non-imputed data using a linear mixed model for gene identification (col G), the consistency score with 1=consistent and 0=not consistent (col H), the affected parameters (col I), and a series of notes (col J).

File Name: Supplementary Data 3. Description of the 85 co-variates.

Description: Column A gives the name of the co-variate, column B indicates the category the co-variate (either mouse information, temporal or categorical), and column C gives the full description of the co-variate.

File Name: Supplementary Data 4. Description and statistics of the 118 brain parameters.

Description: Column A gives the genotype, column B the biological class, column C the subproject name, column D is the acronym given to the brain parameter, column E is the name of the section analyzed, column F gives a description of the brain parameter, and columns G to M provide distribution metrics including variable category, unit, number, minimum, mean, maximum and standard deviation. NA is not applicable.

File Name: Supplementary Data 5. Full raw experimental data.

Description: A total of 6,233 samples were received from the Wellcome Trust Sanger Institute (UK) amongst which 5,086 got successfully analyzed across three coronal sections and 925 across one sagittal section. A unique barcode (col A) identifies the sample without prior knowledge of the genotype. Columns B to DO are the non-imputed data for the 78 brain parameters in coronal and 40 sagittal parameters, columns DP and DQ give body and brain weight, and columns DR to GW provide raw data for the co-variates for each sample. Of note, 19 samples were assigned to a failed genotype (see column DU) bringing the number of samples down to 6214.

File Name: Supplementary Data 6. Genes analyzed by Student’s t-test.

Description: Mutant genes (col A) engineered using a different mouse strain (not C57BL/6) analyzed using a Student’s t-test with their matched colony controls. Column B gives the name of the subproject the samples belong to, column C - age of the mice and column D - the mouse strain used. * denotes the four HOM genes that were exceptionally analyzed versus HET samples (due to missing colony controls).

File Name: Supplementary Data 7. PhenStat vector output for non-imputed data.

Description: Column A gives the full gene name (gene_genotype_allele), followed by the name of the subproject to which the gene belongs to (col B), the number of mutant mice analyzed (col C), the background strain (col D), the age of the mice (col E) and the brain parameter (col F). Columns G to AQ provide the output from a standardized PhenStat R package (Karp N. et al., 2015). It includes whether the jitter was used (col G), the statistical model (col H), the dependent variable (col I), whether the batch effect was significant or not (col J), whether the residual variances were equal or not (col K), the genotype contribution is the p-value obtained from a likelihood ratio test (col L), the effect size estimate (col M), the standard error (col N), the genotype p-value as the result of F ratio test (col O), the percentage change relative to the wild-types (col P), the name of the reference genotype (col W), the normality test for the reference genotype (col X), the name of the test genotype (col Y), the normality test for the test genotype (col Z), the results of the blups test (col AA), the results of the normality test of the rotated residuals (col AB), the estimated coefficient of intercept value (col AC) and its standard error (col AD), whether sex interaction was tested (col AE), a classification tag (col AM), any other additional information (col AO), the unadjusted p-value (p) which is equal to the genotype contribution (col AP) and the adjusted Benjamini-Hochberg (BH-p) p-value (col AQ). NA - not applicable for columns T-V (weight was not used in the model), columns Q-S and AF-AL (only one sex tested) and column AN (no transformation).

File Name: Supplementary Data 8. Mammalian Phenotype terms used to annotate brain abnormalities.

Description: Column A gives the acronym given to the brain parameter, followed by a description of the brain parameter (col B), the parental Mammalian Phenotype (MP) term (col C) and the directional MP term (col D) with their unique identifiers in brackets.

File Name: Supplementary Data 9. Heat map of the association between genes and neuroanatomical phenotypes

Description: Brain parameters are shown along the horizontal axis and studied alleles along the vertical axis. Color intensity indicates the strength of the association based on the Benjamini Hochberg adjusted p-value significance threshold (linear mixed model). Blue color refers to a brain region that is increased is size while red shows a region decreased in size.

File Name: Supplementary Data 10. Statistics for each of the studied alleles in non-imputed data.

Description: Column A gives the full gene name, column B is the category (p, BH-p, morphological percentage change relative to WT or z-score), column C provides the p-value threshold of significance if applicable, followed by the 48 coronal and 40 sagittal brain parameters (columns D to CM) and body and brain weights (columns CO and CP). NA is not applicable.

File Name: Supplementary Data 11. Missing data.

Description: The percentage of missing data is shown for each brain parameter across the four critical sections.

File Name: Supplementary Data 12. Data imputation.

Description: A total of 5,281 samples underwent imputation using a multiple phenotype mixed model imputation method named PHENIX (Dahl A et al., Nature Genetics, 2016). Column A indicates a unique barcode of the sample, columns B to CB provide imputed data for the 39 averaged right and left hemispheres (of section 1 and section 2) as well as for the 40 sagittal parameters.

File Name: Supplementary Data 13. PhenStat vector output for imputed data.

Description: Column A gives the full gene name, followed by the name of subproject to which the gene belongs to (col B), number of mutant mice analyzed (col C), background strain (col D), age of the mice (col E) and the brain parameter (col F). Columns G to AQ provide a standardized PhenStat R package output (Karp N. et al., 2015). It includes whether the jitter was used (col G), statistical model (col H), dependent variable (col I), whether the batch effect was significant (col J), whether residual variances were equal (col K), genotype contribution is the p-value obtained from a likelihood ratio test (col L), effect size estimate (col M), standard error (col N), genotype p-value as the result of F ratio test (col O) and percentage change relative to the wild-types (col P), name of reference genotype (col W), normality test for reference genotype (col X), name of test genotype (col Y), normality test for test genotype (col Z), results of blups test (col AA), results of normality test of rotated residuals (col AB), estimated coefficient of intercept value (col AC) and its standard error (col AD), whether sex interaction was tested (col AE), classification tag (col AM), additional information (col AO), the unadjusted p-value which is equal to the genotype contribution (col AP) and the adjusted p-value (col AQ). NA - not applicable for columns T-V (weight was not used in the model), columns Q-S and AF-AL (only one sex tested) and column AN (no transformation was done).

File Name: Supplementary Data 14. Statistics for each of the studied alleles in imputed data.

Description: Column A gives the full gene name, column B is the category (p-value, BH-p, morphological change relative to WT x 100 for percentage or z-score), column C provides the p-value threshold of significance if applicable, followed by the 39 coronal and 40 sagittal brain parameters (columns D to CD). NA - not applicable.

File Name: Supplementary Data 15. List of 30 ID-associated genes among unique human NAP orthologues.

Description: This list represents the overlap between mouse NAP genes and ID gene lists retrieved from Kochinke et al., 2016 (downloaded in June, 2018). For each overlapping gene (col A), we provide 1) associated human phenotypes as reported in the SysID database (columns B-C), 2) Online Mendelian Inheritance in Man (OMIM) records (columns D-G), 3) literature search for the number of patients and families, ethnicity, and penetrance of the neuroanatomical phenotypes (columns H-N), and 4) a series of information about the mouse genotypes and phenotypes (columns O to V). Of note, columns I and K report first the total number of patients and families, followed by a break down for each Pubmed study (col M) ordered in chronological order within parentheses. Asterisk symbols refer to comments described in column N. AD, autosomal dominant; AR, autosomal recessive; XL, X-linked; NA, not applicable.

File Name: Supplementary Data 16. List of NAP genes mapping to dnCNVs in ID patients with cranial abnormalities.

Description: While patients from both datasets were comprehensively phenotyped, we only report the presence of ID, ASD, psychiatric comorbidities and morphological brain abnormalities. N.B. Except for the individual carrying a mutation in the ARVCF genes, ASD patients from the ASD Iossifov dataset were not assessed for morphological brain abnormalities.

File Name: Supplementary Data 17. Results from clustering NAP genes in various gene networks.

Description: The interconnectedness of NAP genes was compared to that of a 1,000 randomized-genes sets drawn for mutant genes and matched for gene number, coding sequence length and network connectivity. P-values were derived by estimating the proportion of simulations, during which randomized sets were more interconnected than NAP genes. Clustering was assessed using PPI networks, co-expression networks constructed using GTEx and Brainspan data and the PLN. Different correlation coefficient cut-offs were tested for co-expression networks as well as a subset of strongest gene couplings for the PLN.

File Name: Supplementary Data 18. GO enrichment analysis of modules.

Description: Considering each module (col A), we evaluated the over-representations of GO terms using the whole genome as a background. Enrichments were performed using one-sided right-tailed hypergeometric tests and a Benjamini-Hochberg (BH) correction for multiple testing was applied. The table shows GO terms (col B) enriched at BH-p<0.1 or the top 10 terms with smallest p-values (cols C and D) for each module.

File Name: Supplementary Data 19

Description: Genes lists formed.

File Name: Supplementary Data 20. Gene expression datasets.

Description: We examined mouse and human gene expression levels across various body and brain regions and developmental stages. Column D displays the regions or stages considered. When several tissues were used to estimate the average gene expression levels for a given structure, these are displayed in the column E. E.g. while GTEx data provides gene expression levels for both skeletal and tibial muscle tissues, we estimated the average gene expression levels, thereby defining a global muscle expression level.

File Name: Supplementary Data 21. Description of gene networks.

Description: The number of edges and nodes for various gene networks and using many cut-offs (strongest gene coupling for the PLN and correlation coefficients for co-expression networks) are shown here.
