# Supplementary material for: Large-scale neuroanatomical study uncovers 198 gene associations in mouse brain morphogenesis
Source: Nat Commun. 2019 Aug 1;10:3465. doi: 10.1038/s41467-019-11431-2 (PMC6671969; doi:10.1038/s41467-019-11431-2)
Supplement: Supplementary file 13 — Supplementary Data 9 [file 41467_2019_11431_MOESM13_ESM.pdf]

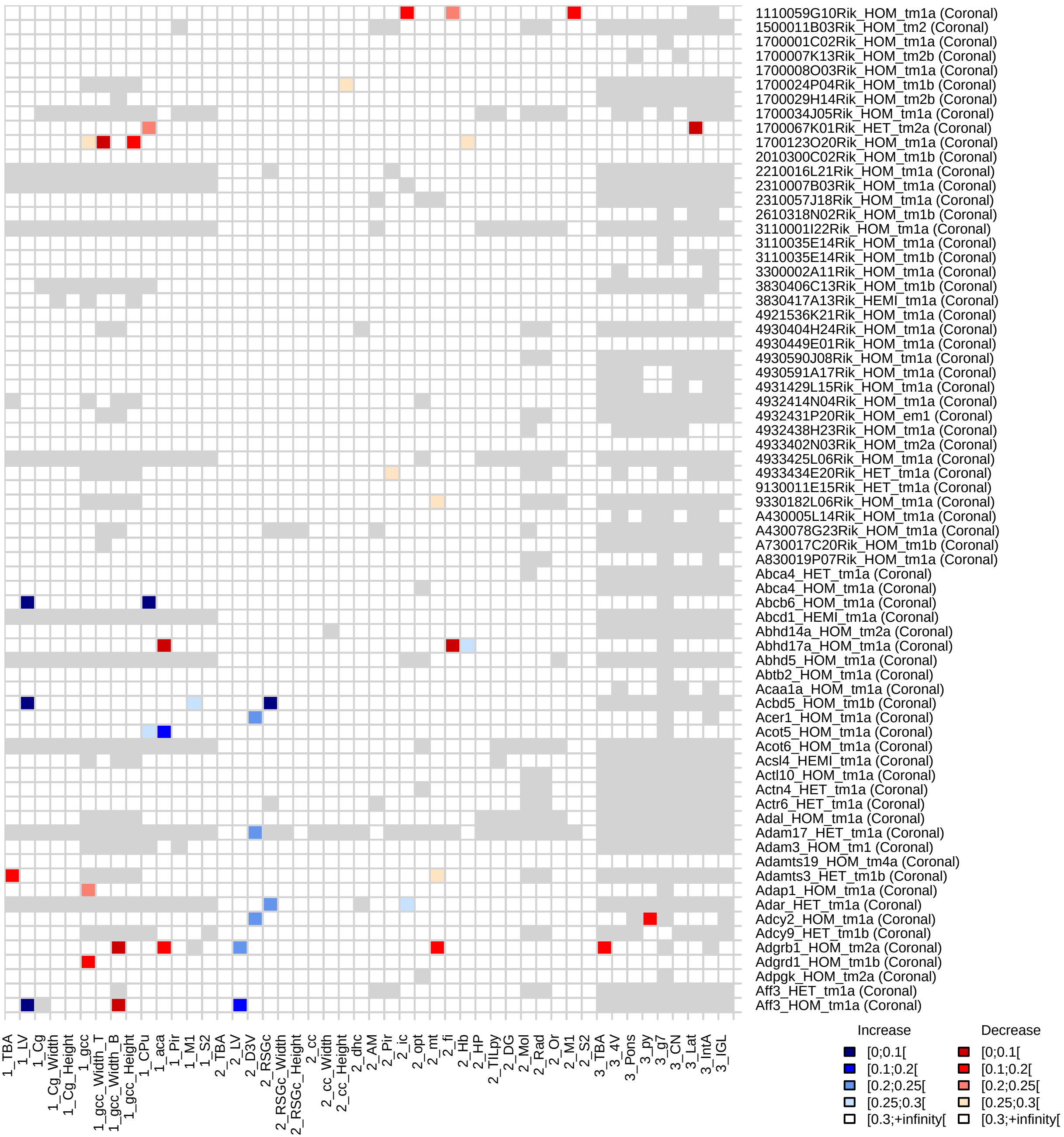
1110059G10Rik_HOM_tm1a (Coronal)
1500011B03Rik_HOM_tm2 (Coronal)
1700001C02Rik_HOM_tm1a (Coronal)
1700007K13Rik_HOM_tm2b (Coronal)
1700008O03Rik_HOM_tm1a (Coronal)
1700024P04Rik_HOM_tm1b (Coronal)
1700029H14Rik_HOM_tm2b (Coronal)
1700034J05Rik_HOM_tm1a (Coronal)
1700067K01Rik_HET_tm2a (Coronal)
1700123O20Rik_HOM_tm1a (Coronal)
2010300C02Rik_HOM_tm1b (Coronal)
2210016L21Rik_HOM_tm1a (Coronal)
2310007B03Rik_HOM_tm1a (Coronal)
2310057J18Rik_HOM_tm1a (Coronal)
2610318N02Rik_HOM_tm1b (Coronal)
3110001I22Rik_HOM_tm1a (Coronal)
3110035E14Rik_HOM_tm1a (Coronal)
3110035E14Rik_HOM_tm1b (Coronal)
3300002A11Rik_HOM_tm1a (Coronal)
3830406C13Rik_HOM_tm1b (Coronal)
3830417A13Rik_HEMI_tm1a (Coronal)
4921536K21Rik_HOM_tm1a (Coronal)
4930404H24Rik_HOM_tm1a (Coronal)
4930449E01Rik_HOM_tm1a (Coronal)
4930590J08Rik_HOM_tm1a (Coronal)
4930591A17Rik_HOM_tm1a (Coronal)
4931429L15Rik_HOM_tm1a (Coronal)
4932414N04Rik_HOM_tm1a (Coronal)
4932431P20Rik_HOM_em1 (Coronal)
4932438H23Rik_HOM_tm1a (Coronal)
4933402N03Rik_HOM_tm2a (Coronal)
4933425L06Rik_HOM_tm1a (Coronal)
4933434E20Rik_HET_tm1a (Coronal)
9130011E15Rik_HET_tm1a (Coronal)
9330182L06Rik_HOM_tm1a (Coronal)
A430005L14Rik_HOM_tm1a (Coronal)
A430078G23Rik_HOM_tm1a (Coronal)
A730017C20Rik_HOM_tm1b (Coronal)
A830019P07Rik_HOM_tm1a (Coronal)
Abca4_HET_tm1a (Coronal)
Abca4_HOM_tm1a (Coronal)
Abcb6_HOM_tm1a (Coronal)
Abcd1_HEMI_tm1a (Coronal)
Abhd14a_HOM_tm2a (Coronal)
Abhd17a_HOM_tm1a (Coronal)
Abhd5_HOM_tm1a (Coronal)
Abtb2_HOM_tm1a (Coronal)
Acaa1a_HOM_tm1a (Coronal)
Acbd5_HOM_tm1b (Coronal)
Acer1_HOM_tm1a (Coronal)
Acot5_HOM_tm1a (Coronal)
Acot6_HOM_tm1a (Coronal)
Acsl4_HEMI_tm1a (Coronal)
Actl10_HOM_tm1a (Coronal)
Actn4_HET_tm1a (Coronal)
Actr6_HET_tm1a (Coronal)
Adal_HOM_tm1a (Coronal)
Adam17_HET_tm1a (Coronal)
Adam3_HOM_tm1 (Coronal)
Adamts19_HOM_tm4a (Coronal)
Adamts3_HET_tm1b (Coronal)
Adap1_HOM_tm1a (Coronal)
Adar_HET_tm1a (Coronal)
Adcy2_HOM_tm1a (Coronal)
Adcy9_HET_tm1b (Coronal)
Adgrb1_HOM_tm2a (Coronal)
Adgrd1_HOM_tm1b (Coronal)
Adpgk_HOM_tm2a (Coronal)
Aff3_HET_tm1a (Coronal)
Aff3_HOM_tm1a (Coronal)
1_TBA
1_LV
1_Cg
1_Cg_Width
1_Cg_Height
1_gcc
1_gcc_Width_T
1_gcc_Width_B
1_gcc_Height
1_CPu
1_aca
1_Pir
1_M1
1_S2
2_TBA
2_LV
2_D3V
2_RSGc
2_RSGc_Width
2_RSGc_Height
2_cc
2_cc_Width
2_cc_Height
2_dhc
2_AM
2_Pir
2_ic
2_opt
2_mt
2_fi
2_Hb
2_HP
2_TILpy
2_DG
2_Mol
2_Rad
2_Or
2_M1
2_S2
3_TBA
3_4V
3_Pons
3_py
3_g7
3_CN
3_Lat
3_IntA
3_IGL
Increase
[0;0.1[
[0.1;0.2[
[0.2;0.25[
[0.25;0.3[
[0.3;+infinity[
Decrease
[0;0.1[
[0.1;0.2[
[0.2;0.25[
[0.25;0.3[
[0.3;+infinity[

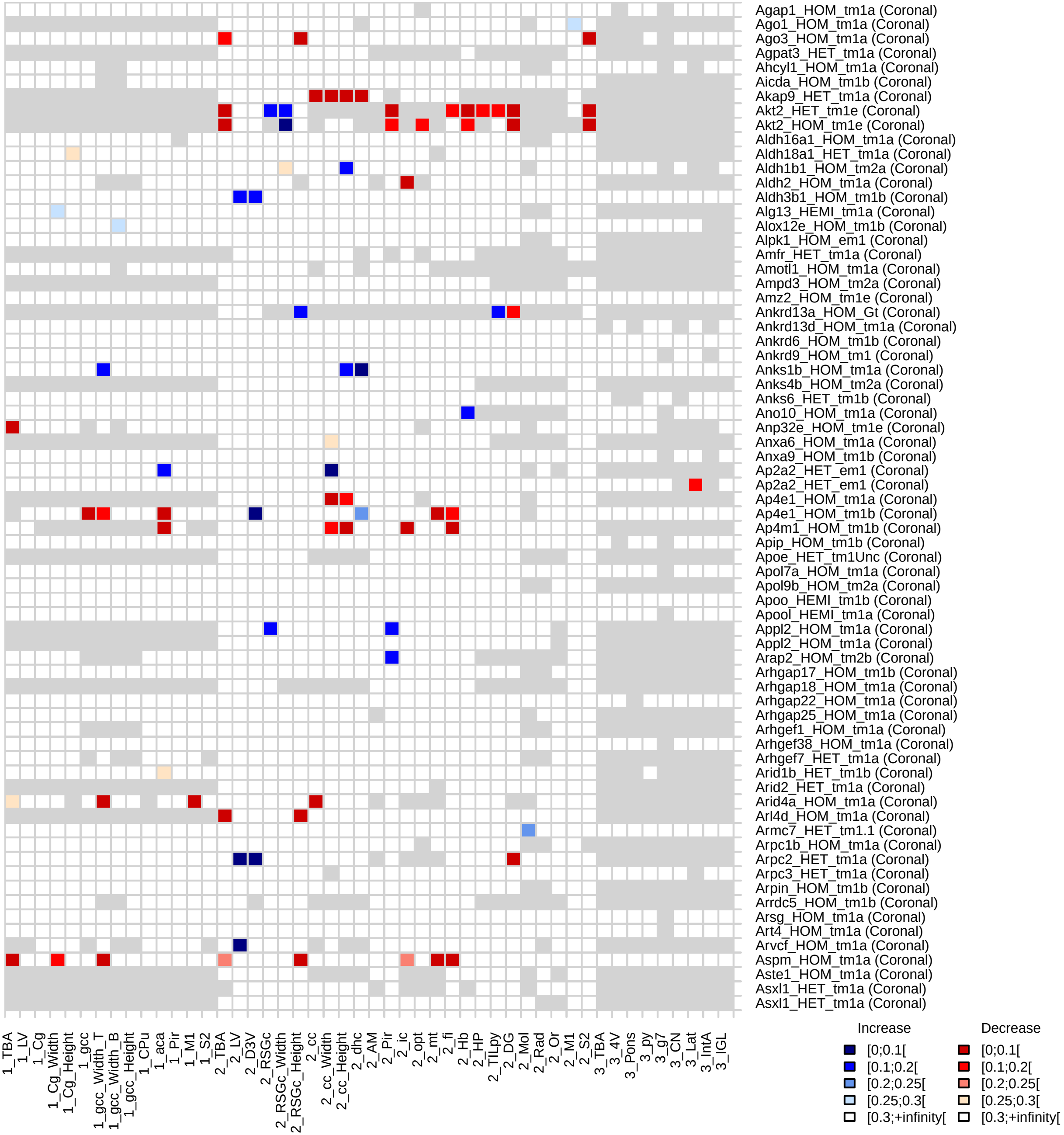
Agap1_HOM_tm1a (Coronal)
Ago1_HOM_tm1a (Coronal)
Ago3_HOM_tm1a (Coronal)
Agpat3_HET_tm1a (Coronal)
Ahcyl1_HOM_tm1a (Coronal)
Aicda_HOM_tm1b (Coronal)
Akap9_HET_tm1a (Coronal)
Akt2_HET_tm1e (Coronal)
Akt2_HOM_tm1e (Coronal)
Aldh16a1_HOM_tm1a (Coronal)
Aldh18a1_HET_tm1a (Coronal)
Aldh1b1_HOM_tm2a (Coronal)
Aldh2_HOM_tm1a (Coronal)
Aldh3b1_HOM_tm1b (Coronal)
Alg13_HEMI_tm1a (Coronal)
Alox12e_HOM_tm1b (Coronal)
Alpk1_HOM_em1 (Coronal)
Amfr_HET_tm1a (Coronal)
Amotl1_HOM_tm1a (Coronal)
Ampd3_HOM_tm2a (Coronal)
Amz2_HOM_tm1e (Coronal)
Ankrd13a_HOM_Gt (Coronal)
Ankrd13d_HOM_tm1a (Coronal)
Ankrd6_HOM_tm1b (Coronal)
Ankrd9_HOM_tm1 (Coronal)
Anks1b_HOM_tm1a (Coronal)
Anks4b_HOM_tm2a (Coronal)
Anks6_HET_tm1b (Coronal)
Ano10_HOM_tm1a (Coronal)
Anp32e_HOM_tm1e (Coronal)
Anxa6_HOM_tm1a (Coronal)
Anxa9_HOM_tm1b (Coronal)
Ap2a2_HET_em1 (Coronal)
Ap2a2_HET_em1 (Coronal)
Ap4e1_HOM_tm1a (Coronal)
Ap4e1_HOM_tm1b (Coronal)
Ap4m1_HOM_tm1b (Coronal)
Apip_HOM_tm1b (Coronal)
Apoe_HET_tm1Unc (Coronal)
Apol7a_HOM_tm1a (Coronal)
Apol9b_HOM_tm2a (Coronal)
Apoo_HEMI_tm1b (Coronal)
Apool_HEMI_tm1a (Coronal)
Appl2_HOM_tm1a (Coronal)
Appl2_HOM_tm1a (Coronal)
Arap2_HOM_tm2b (Coronal)
Arhgap17_HOM_tm1b (Coronal)
Arhgap18_HOM_tm1a (Coronal)
Arhgap22_HOM_tm1a (Coronal)
Arhgap25_HOM_tm1a (Coronal)
Arhgef1_HOM_tm1a (Coronal)
Arhgef38_HOM_tm1a (Coronal)
Arhgef7_HET_tm1a (Coronal)
Arid1b_HET_tm1b (Coronal)
Arid2_HET_tm1a (Coronal)
Arid4a_HOM_tm1a (Coronal)
Arl4d_HOM_tm1a (Coronal)
Armc7_HET_tm1.1 (Coronal)
Arpc1b_HOM_tm1a (Coronal)
Arpc2_HET_tm1a (Coronal)
Arpc3_HET_tm1a (Coronal)
Arpin_HOM_tm1b (Coronal)
Arrdc5_HOM_tm1b (Coronal)
Arsg_HOM_tm1a (Coronal)
Art4_HOM_tm1a (Coronal)
Arvcf_HOM_tm1a (Coronal)
Aspm_HOM_tm1a (Coronal)
Aste1_HOM_tm1a (Coronal)
Asxl1_HET_tm1a (Coronal)
Asxl1_HET_tm1a (Coronal)
1_TBA
1_LV
1_Cg
1_Cg_Width
1_Cg_Height
1_gcc
1_gcc_Width_T
1_gcc_Width_B
1_gcc_Height
1_CPu
1_aca
1_Pir
1_M1
1_S2
2_TBA
2_LV
2_D3V
2_RSGc
2_RSGc_Width
2_RSGc_Height
2_cc
2_cc_Width
2_cc_Height
2_dhc
2_AM
2_Pir
2_ic
2_opt
2_mt
2_fi
2_Hb
2_HP
2_TILpy
2_DG
2_Mol
2_Rad
2_Or
2_M1
2_S2
3_TBA
3_4V
3_Pons
3_py
3_g7
3_CN
3_Lat
3_IntA
3_IGL
Increase
[0;0.1[
[0.1;0.2[
[0.2;0.25[
[0.25;0.3[
[0.3;+infinity[
Decrease
[0;0.1[
[0.1;0.2[
[0.2;0.25[
[0.25;0.3[
[0.3;+infinity[

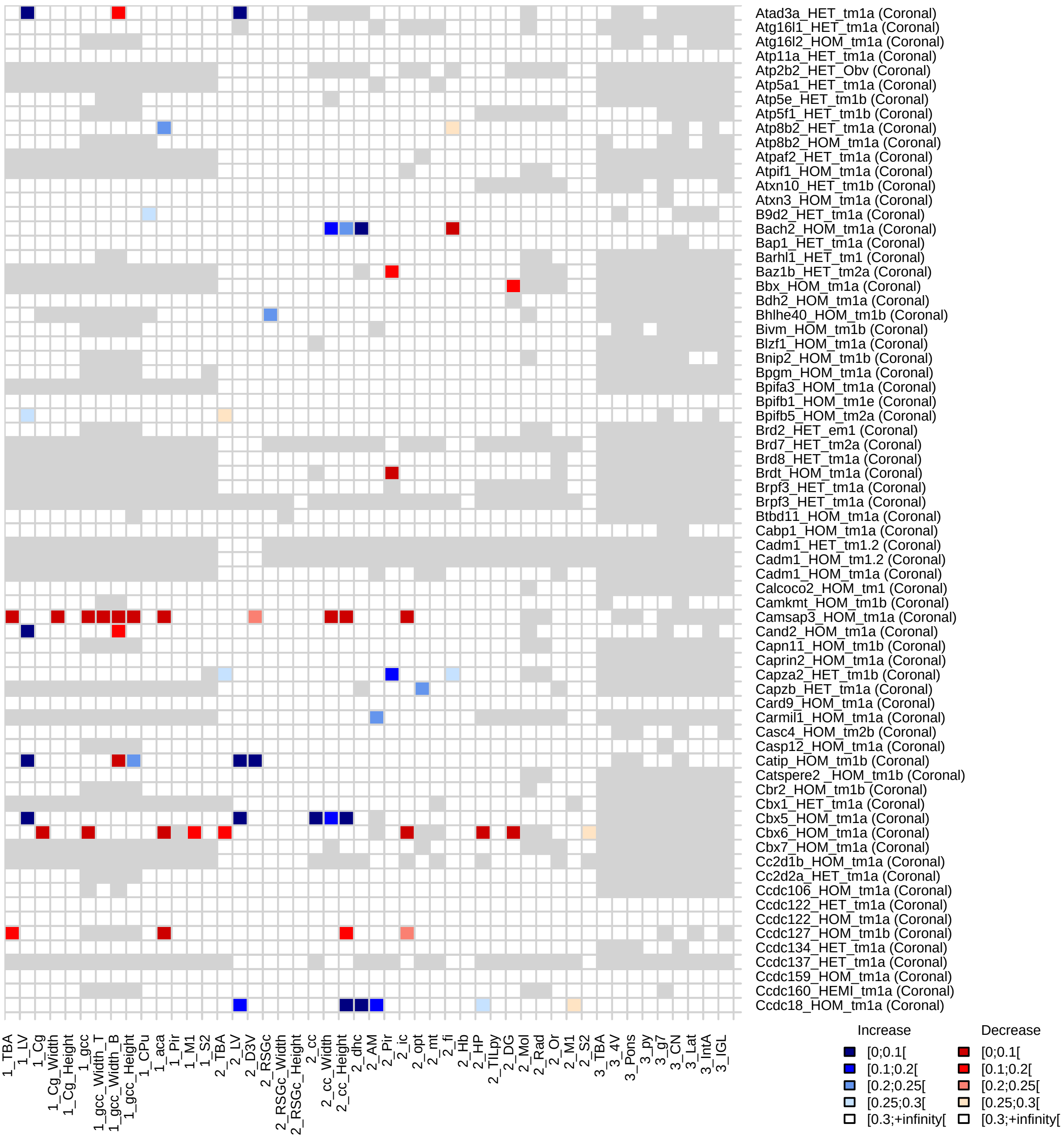
Atad3a_HET_tm1a (Coronal)
Atg16l1_HET_tm1a (Coronal)
Atg16l2_HOM_tm1a (Coronal)
Atp11a_HET_tm1a (Coronal)
Atp2b2_HET_Obv (Coronal)
Atp5a1_HET_tm1a (Coronal)
Atp5e_HET_tm1b (Coronal)
Atp5f1_HET_tm1b (Coronal)
Atp8b2_HET_tm1a (Coronal)
Atp8b2_HOM_tm1a (Coronal)
Atpaf2_HET_tm1a (Coronal)
Atpif1_HOM_tm1a (Coronal)
Atxn10_HET_tm1b (Coronal)
Atxn3_HOM_tm1a (Coronal)
B9d2_HET_tm1a (Coronal)
Bach2_HOM_tm1a (Coronal)
Bap1_HET_tm1a (Coronal)
Barhl1_HET_tm1 (Coronal)
Baz1b_HET_tm2a (Coronal)
Bbx_HOM_tm1a (Coronal)
Bdh2_HOM_tm1a (Coronal)
Bhlhe40_HOM_tm1b (Coronal)
Bivm_HOM_tm1b (Coronal)
Blzf1_HOM_tm1a (Coronal)
Bnip2_HOM_tm1b (Coronal)
Bpgm_HOM_tm1a (Coronal)
Bpifa3_HOM_tm1a (Coronal)
Bpifb1_HOM_tm1e (Coronal)
Bpifb5_HOM_tm2a (Coronal)
Brd2_HET_em1 (Coronal)
Brd7_HET_tm2a (Coronal)
Brd8_HET_tm1a (Coronal)
Brdt_HOM_tm1a (Coronal)
Brpf3_HET_tm1a (Coronal)
Brpf3_HET_tm1a (Coronal)
Btbd11_HOM_tm1a (Coronal)
Cabp1_HOM_tm1a (Coronal)
Cadm1_HET_tm1.2 (Coronal)
Cadm1_HOM_tm1.2 (Coronal)
Cadm1_HOM_tm1a (Coronal)
Calcoco2_HOM_tm1 (Coronal)
Camkmt_HOM_tm1b (Coronal)
Camsap3_HOM_tm1a (Coronal)
Cand2_HOM_tm1a (Coronal)
Capn11_HOM_tm1b (Coronal)
Caprin2_HOM_tm1a (Coronal)
Capza2_HET_tm1b (Coronal)
Capzb_HET_tm1a (Coronal)
Card9_HOM_tm1a (Coronal)
Carmil1_HOM_tm1a (Coronal)
Casc4_HOM_tm2b (Coronal)
Casp12_HOM_tm1a (Coronal)
Catip_HOM_tm1b (Coronal)
Catspere2 _HOM_tm1b (Coronal)
Cbr2_HOM_tm1b (Coronal)
Cbx1_HET_tm1a (Coronal)
Cbx5_HOM_tm1a (Coronal)
Cbx6_HOM_tm1a (Coronal)
Cbx7_HOM_tm1a (Coronal)
Cc2d1b_HOM_tm1a (Coronal)
Cc2d2a_HET_tm1a (Coronal)
Ccdc106_HOM_tm1a (Coronal)
Ccdc122_HET_tm1a (Coronal)
Ccdc122_HOM_tm1a (Coronal)
Ccdc127_HOM_tm1b (Coronal)
Ccdc134_HET_tm1a (Coronal)
Ccdc137_HET_tm1a (Coronal)
Ccdc159_HOM_tm1a (Coronal)
Ccdc160_HEMI_tm1a (Coronal)
Ccdc18_HOM_tm1a (Coronal)
1_TBA
1_LV
1_Cg
1_Cg_Width
1_Cg_Height
1_gcc
1_gcc_Width_T
1_gcc_Width_B
1_gcc_Height
1_CPu
1_aca
1_Pir
1_M1
1_S2
2_TBA
2_LV
2_D3V
2_RSGc
2_RSGc_Width
2_RSGc_Height
2_cc
2_cc_Width
2_cc_Height
2_dhc
2_AM
2_Pir
2_ic
2_opt
2_mt
2_fi
2_Hb
2_HP
2_TILpy
2_DG
2_Mol
2_Rad
2_Or
2_M1
2_S2
3_TBA
3_4V
3_Pons
3_py
3_g7
3_CN
3_Lat
3_IntA
3_IGL
Increase
[0;0.1[
[0.1;0.2[
[0.2;0.25[
[0.25;0.3[
[0.3;+infinity[
Decrease
[0;0.1[
[0.1;0.2[
[0.2;0.25[
[0.25;0.3[
[0.3;+infinity[

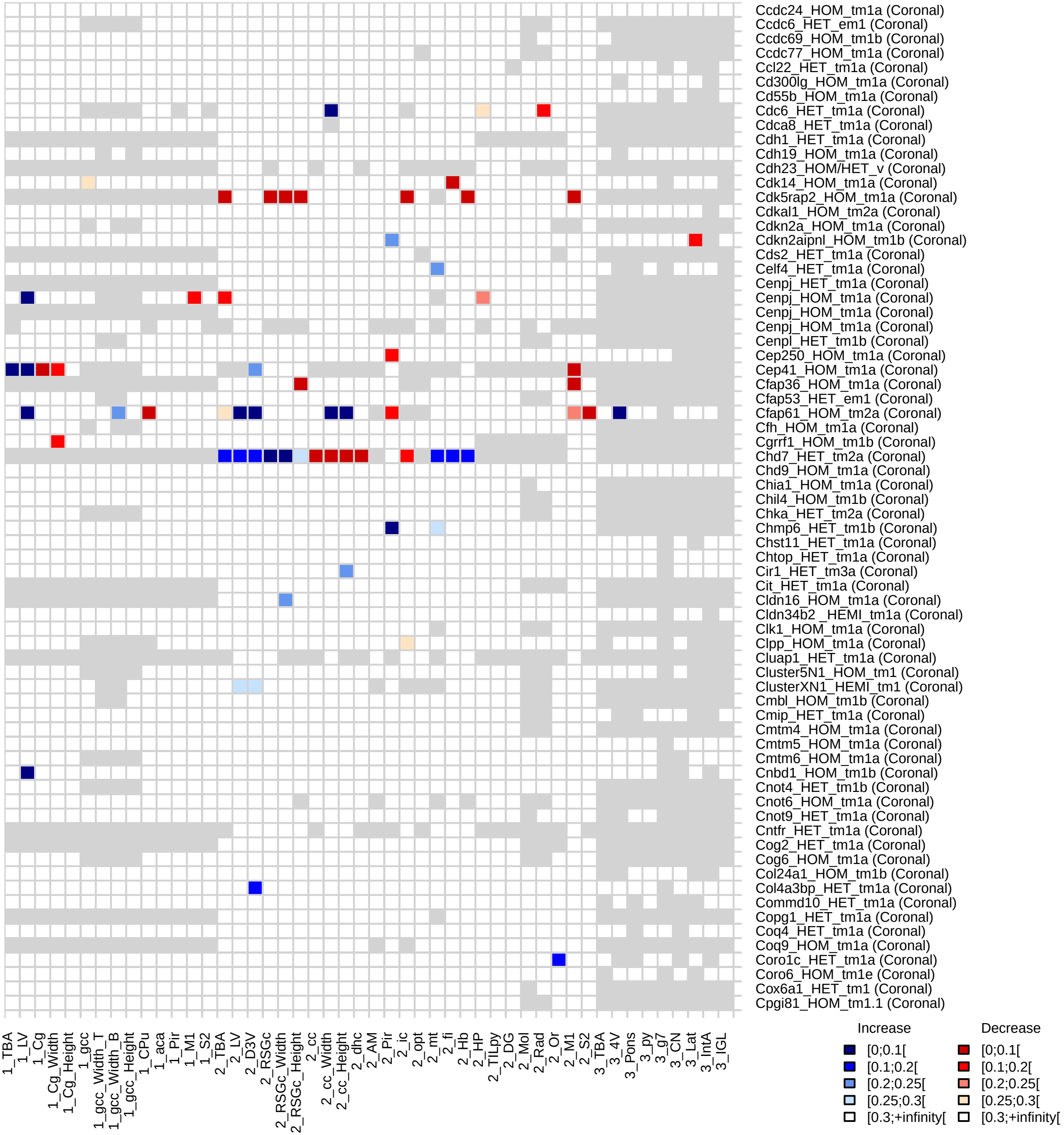
Ccdc24_HOM_tm1a (Coronal)
Ccdc6_HET_em1 (Coronal)
Ccdc69_HOM_tm1b (Coronal)
Ccdc77_HOM_tm1a (Coronal)
Ccl22_HET_tm1a (Coronal)
Cd300lg_HOM_tm1a (Coronal)
Cd55b_HOM_tm1a (Coronal)
Cdc6_HET_tm1a (Coronal)
Cdca8_HET_tm1a (Coronal)
Cdh1_HET_tm1a (Coronal)
Cdh19_HOM_tm1a (Coronal)
Cdh23_HOM/HET_v (Coronal)
Cdk14_HOM_tm1a (Coronal)
Cdk5rap2_HOM_tm1a (Coronal)
Cdkal1_HOM_tm2a (Coronal)
Cdkn2a_HOM_tm1a (Coronal)
Cdkn2aipnl_HOM_tm1b (Coronal)
Cds2_HET_tm1a (Coronal)
Celf4_HET_tm1a (Coronal)
Cenpj_HET_tm1a (Coronal)
Cenpj_HOM_tm1a (Coronal)
Cenpj_HOM_tm1a (Coronal)
Cenpj_HOM_tm1a (Coronal)
Cenpl_HET_tm1b (Coronal)
Cep250_HOM_tm1a (Coronal)
Cep41_HOM_tm1a (Coronal)
Cfap36_HOM_tm1a (Coronal)
Cfap53_HET_em1 (Coronal)
Cfap61_HOM_tm2a (Coronal)
Cfh_HOM_tm1a (Coronal)
Cgrrf1_HOM_tm1b (Coronal)
Chd7_HET_tm2a (Coronal)
Chd9_HOM_tm1a (Coronal)
Chia1_HOM_tm1a (Coronal)
Chil4_HOM_tm1b (Coronal)
Chka_HET_tm2a (Coronal)
Chmp6_HET_tm1b (Coronal)
Chst11_HET_tm1a (Coronal)
Chtop_HET_tm1a (Coronal)
Cir1_HET_tm3a (Coronal)
Cit_HET_tm1a (Coronal)
Cldn16_HOM_tm1a (Coronal)
Cldn34b2 _HEMI_tm1a (Coronal)
Clk1_HOM_tm1a (Coronal)
Clpp_HOM_tm1a (Coronal)
Cluap1_HET_tm1a (Coronal)
Cluster5N1_HOM_tm1 (Coronal)
ClusterXN1_HEMI_tm1 (Coronal)
Cmbl_HOM_tm1b (Coronal)
Cmip_HET_tm1a (Coronal)
Cmtm4_HOM_tm1a (Coronal)
Cmtm5_HOM_tm1a (Coronal)
Cmtm6_HOM_tm1a (Coronal)
Cnbd1_HOM_tm1b (Coronal)
Cnot4_HET_tm1b (Coronal)
Cnot6_HOM_tm1a (Coronal)
Cnot9_HET_tm1a (Coronal)
Cntfr_HET_tm1a (Coronal)
Cog2_HET_tm1a (Coronal)
Cog6_HOM_tm1a (Coronal)
Col24a1_HOM_tm1b (Coronal)
Col4a3bp_HET_tm1a (Coronal)
Commd10_HET_tm1a (Coronal)
Copg1_HET_tm1a (Coronal)
Coq4_HET_tm1a (Coronal)
Coq9_HOM_tm1a (Coronal)
Coro1c_HET_tm1a (Coronal)
Coro6_HOM_tm1e (Coronal)
Cox6a1_HET_tm1 (Coronal)
Cpgi81_HOM_tm1.1 (Coronal)
1_TBA
1_LV
1_Cg
1_Cg_Width
1_Cg_Height
1_gcc
1_gcc_Width_T
1_gcc_Width_B
1_gcc_Height
1_CPu
1_aca
1_Pir
1_M1
1_S2
2_TBA
2_LV
2_D3V
2_RSGc
2_RSGc_Width
2_RSGc_Height
2_cc
2_cc_Width
2_cc_Height
2_dhc
2_AM
2_Pir
2_ic
2_opt
2_mt
2_fi
2_Hb
2_HP
2_TILpy
2_DG
2_Mol
2_Rad
2_Or
2_M1
2_S2
3_TBA
3_4V
3_Pons
3_py
3_g7
3_CN
3_Lat
3_IntA
3_IGL
Increase
[0;0.1[
[0.1;0.2[
[0.2;0.25[
[0.25;0.3[
[0.3;+infinity[
Decrease
[0;0.1[
[0.1;0.2[
[0.2;0.25[
[0.25;0.3[
[0.3;+infinity[

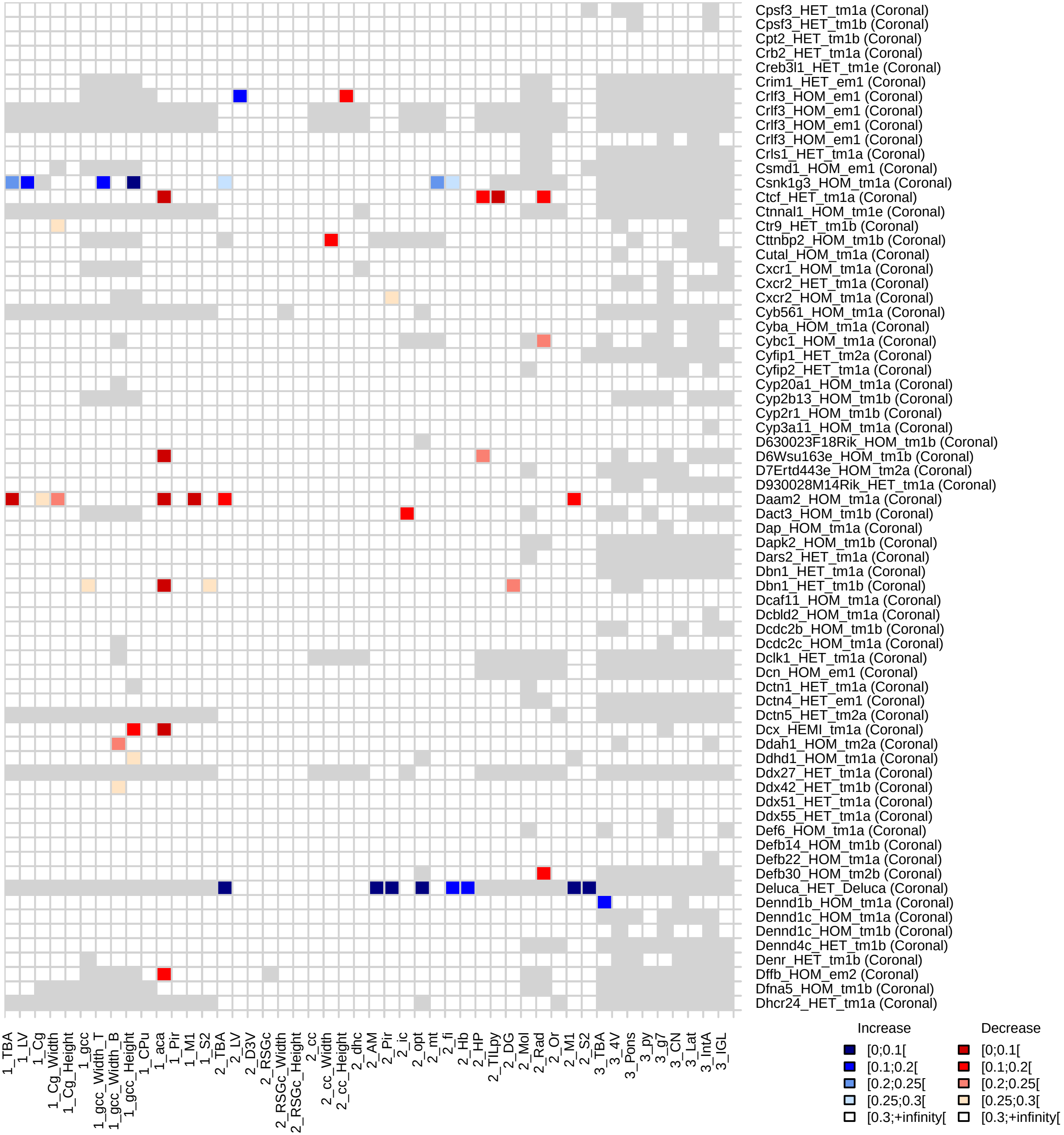
Cpsf3_HET_tm1a (Coronal)
Cpsf3_HET_tm1b (Coronal)
Cpt2_HET_tm1b (Coronal)
Crb2_HET_tm1a (Coronal)
Creb3l1_HET_tm1e (Coronal)
Crim1_HET_em1 (Coronal)
Crlf3_HOM_em1 (Coronal)
Crlf3_HOM_em1 (Coronal)
Crlf3_HOM_em1 (Coronal)
Crlf3_HOM_em1 (Coronal)
Crls1_HET_tm1a (Coronal)
Csmd1_HOM_em1 (Coronal)
Csnk1g3_HOM_tm1a (Coronal)
Ctcf_HET_tm1a (Coronal)
Ctnnal1_HOM_tm1e (Coronal)
Ctr9_HET_tm1b (Coronal)
Cttnbp2_HOM_tm1b (Coronal)
Cutal_HOM_tm1a (Coronal)
Cxcr1_HOM_tm1a (Coronal)
Cxcr2_HET_tm1a (Coronal)
Cxcr2_HOM_tm1a (Coronal)
Cyb561_HOM_tm1a (Coronal)
Cyba_HOM_tm1a (Coronal)
Cybc1_HOM_tm1a (Coronal)
Cyfip1_HET_tm2a (Coronal)
Cyfip2_HET_tm1a (Coronal)
Cyp20a1_HOM_tm1a (Coronal)
Cyp2b13_HOM_tm1b (Coronal)
Cyp2r1_HOM_tm1b (Coronal)
Cyp3a11_HOM_tm1a (Coronal)
D630023F18Rik_HOM_tm1b (Coronal)
D6Wsu163e_HOM_tm1b (Coronal)
D7Ertd443e_HOM_tm2a (Coronal)
D930028M14Rik_HET_tm1a (Coronal)
Daam2_HOM_tm1a (Coronal)
Dact3_HOM_tm1b (Coronal)
Dap_HOM_tm1a (Coronal)
Dapk2_HOM_tm1b (Coronal)
Dars2_HET_tm1a (Coronal)
Dbn1_HET_tm1a (Coronal)
Dbn1_HET_tm1b (Coronal)
Dcaf11_HOM_tm1a (Coronal)
Dcbld2_HOM_tm1a (Coronal)
Dcdc2b_HOM_tm1b (Coronal)
Dcdc2c_HOM_tm1a (Coronal)
Dclk1_HET_tm1a (Coronal)
Dcn_HOM_em1 (Coronal)
Dctn1_HET_tm1a (Coronal)
Dctn4_HET_em1 (Coronal)
Dctn5_HET_tm2a (Coronal)
Dcx_HEMI_tm1a (Coronal)
Ddah1_HOM_tm2a (Coronal)
Ddhd1_HOM_tm1a (Coronal)
Ddx27_HET_tm1a (Coronal)
Ddx42_HET_tm1b (Coronal)
Ddx51_HET_tm1a (Coronal)
Ddx55_HET_tm1a (Coronal)
Def6_HOM_tm1a (Coronal)
Defb14_HOM_tm1b (Coronal)
Defb22_HOM_tm1a (Coronal)
Defb30_HOM_tm2b (Coronal)
Deluca_HET_Deluca (Coronal)
Dennd1b_HOM_tm1a (Coronal)
Dennd1c_HOM_tm1a (Coronal)
Dennd1c_HOM_tm1b (Coronal)
Dennd4c_HET_tm1b (Coronal)
Denr_HET_tm1b (Coronal)
Dffb_HOM_em2 (Coronal)
Dfna5_HOM_tm1b (Coronal)
Dhcr24_HET_tm1a (Coronal)
1_TBA
1_LV
1_Cg
1_Cg_Width
1_Cg_Height
1_gcc
1_gcc_Width_T
1_gcc_Width_B
1_gcc_Height
1_CPu
1_aca
1_Pir
1_M1
1_S2
2_TBA
2_LV
2_D3V
2_RSGc
2_RSGc_Width
2_RSGc_Height
2_cc
2_cc_Width
2_cc_Height
2_dhc
2_AM
2_Pir
2_ic
2_opt
2_mt
2_fi
2_Hb
2_HP
2_TILpy
2_DG
2_Mol
2_Rad
2_Or
2_M1
2_S2
3_TBA
3_4V
3_Pons
3_py
3_g7
3_CN
3_Lat
3_IntA
3_IGL
Increase
[0;0.1[
[0.1;0.2[
[0.2;0.25[
[0.25;0.3[
[0.3;+infinity[
Decrease
[0;0.1[
[0.1;0.2[
[0.2;0.25[
[0.25;0.3[
[0.3;+infinity[

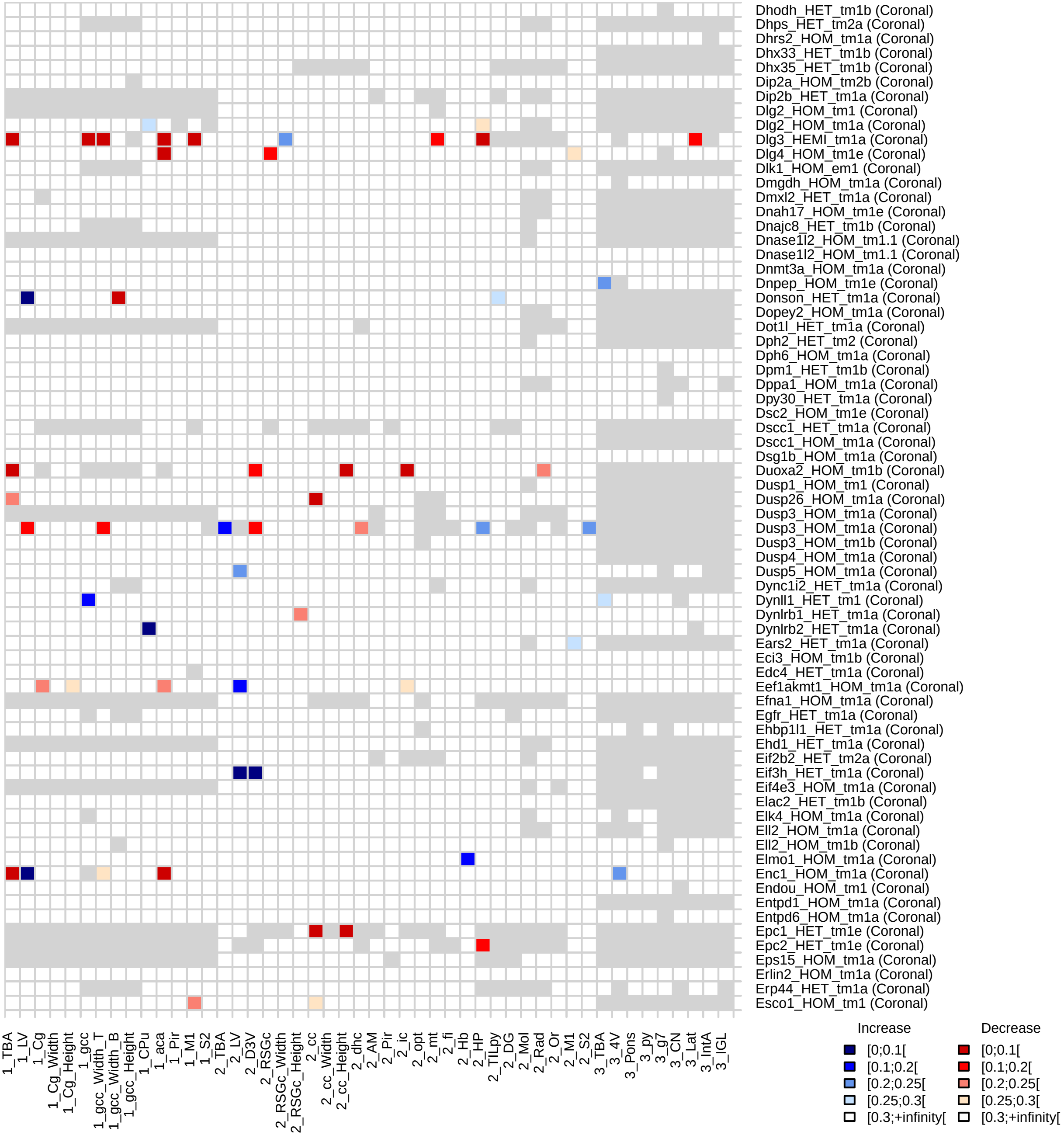
Dhodh_HET_tm1b (Coronal)
Dhps_HET_tm2a (Coronal)
Dhrs2_HOM_tm1a (Coronal)
Dhx33_HET_tm1b (Coronal)
Dhx35_HET_tm1b (Coronal)
Dip2a_HOM_tm2b (Coronal)
Dip2b_HET_tm1a (Coronal)
Dlg2_HOM_tm1 (Coronal)
Dlg2_HOM_tm1a (Coronal)
Dlg3_HEMI_tm1a (Coronal)
Dlg4_HOM_tm1e (Coronal)
Dlk1_HOM_em1 (Coronal)
Dmgdh_HOM_tm1a (Coronal)
Dmxl2_HET_tm1a (Coronal)
Dnah17_HOM_tm1e (Coronal)
Dnajc8_HET_tm1b (Coronal)
Dnase1l2_HOM_tm1.1 (Coronal)
Dnase1l2_HOM_tm1.1 (Coronal)
Dnmt3a_HOM_tm1a (Coronal)
Dnpep_HOM_tm1e (Coronal)
Donson_HET_tm1a (Coronal)
Dopey2_HOM_tm1a (Coronal)
Dot1l_HET_tm1a (Coronal)
Dph2_HET_tm2 (Coronal)
Dph6_HOM_tm1a (Coronal)
Dpm1_HET_tm1b (Coronal)
Dppa1_HOM_tm1a (Coronal)
Dpy30_HET_tm1a (Coronal)
Dsc2_HOM_tm1e (Coronal)
Dscc1_HET_tm1a (Coronal)
Dscc1_HOM_tm1a (Coronal)
Dsg1b_HOM_tm1a (Coronal)
Duoxa2_HOM_tm1b (Coronal)
Dusp1_HOM_tm1 (Coronal)
Dusp26_HOM_tm1a (Coronal)
Dusp3_HOM_tm1a (Coronal)
Dusp3_HOM_tm1a (Coronal)
Dusp3_HOM_tm1b (Coronal)
Dusp4_HOM_tm1a (Coronal)
Dusp5_HOM_tm1a (Coronal)
Dync1i2_HET_tm1a (Coronal)
Dynll1_HET_tm1 (Coronal)
Dynlrb1_HET_tm1a (Coronal)
Dynlrb2_HET_tm1a (Coronal)
Ears2_HET_tm1a (Coronal)
Eci3_HOM_tm1b (Coronal)
Edc4_HET_tm1a (Coronal)
Eef1akmt1_HOM_tm1a (Coronal)
Efna1_HOM_tm1a (Coronal)
Egfr_HET_tm1a (Coronal)
Ehbp1l1_HET_tm1a (Coronal)
Ehd1_HET_tm1a (Coronal)
Eif2b2_HET_tm2a (Coronal)
Eif3h_HET_tm1a (Coronal)
Eif4e3_HOM_tm1a (Coronal)
Elac2_HET_tm1b (Coronal)
Elk4_HOM_tm1a (Coronal)
Ell2_HOM_tm1a (Coronal)
Ell2_HOM_tm1b (Coronal)
Elmo1_HOM_tm1a (Coronal)
Enc1_HOM_tm1a (Coronal)
Endou_HOM_tm1 (Coronal)
Entpd1_HOM_tm1a (Coronal)
Entpd6_HOM_tm1a (Coronal)
Epc1_HET_tm1e (Coronal)
Epc2_HET_tm1e (Coronal)
Eps15_HOM_tm1a (Coronal)
Erlin2_HOM_tm1a (Coronal)
Erp44_HET_tm1a (Coronal)
Esco1_HOM_tm1 (Coronal)
1_TBA
1_LV
1_Cg
1_Cg_Width
1_Cg_Height
1_gcc
1_gcc_Width_T
1_gcc_Width_B
1_gcc_Height
1_CPu
1_aca
1_Pir
1_M1
1_S2
2_TBA
2_LV
2_D3V
2_RSGc
2_RSGc_Width
2_RSGc_Height
2_cc
2_cc_Width
2_cc_Height
2_dhc
2_AM
2_Pir
2_ic
2_opt
2_mt
2_fi
2_Hb
2_HP
2_TILpy
2_DG
2_Mol
2_Rad
2_Or
2_M1
2_S2
3_TBA
3_4V
3_Pons
3_py
3_g7
3_CN
3_Lat
3_IntA
3_IGL
Increase
[0;0.1[
[0.1;0.2[
[0.2;0.25[
[0.25;0.3[
[0.3;+infinity[
Decrease
[0;0.1[
[0.1;0.2[
[0.2;0.25[
[0.25;0.3[
[0.3;+infinity[

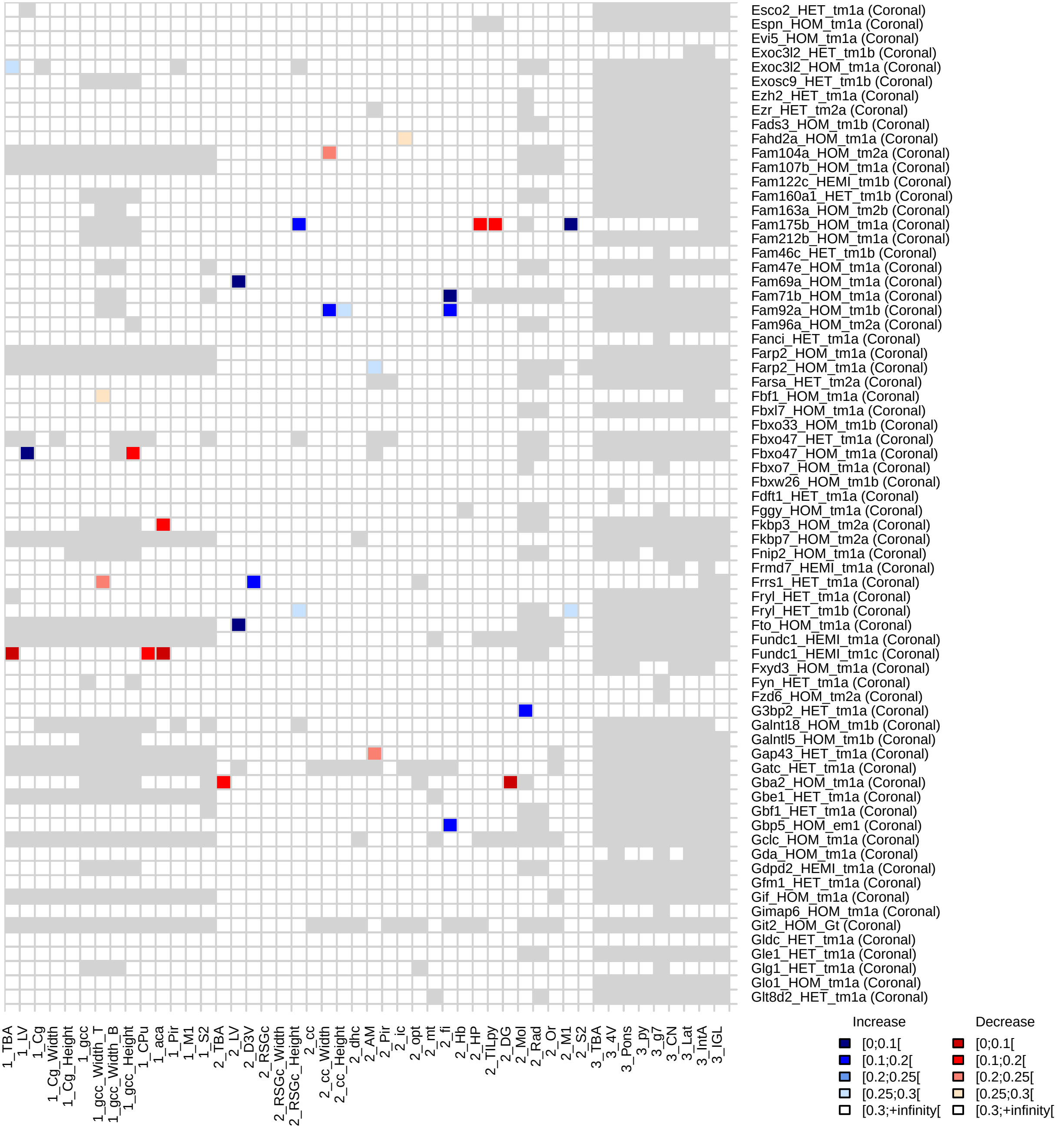

Esco2_HET_tm1a (Coronal)
Espn_HOM_tm1a (Coronal)
Evi5_HOM_tm1a (Coronal)
Exoc3l2_HET_tm1b (Coronal)
Exoc3l2_HOM_tm1a (Coronal)
Exosc9_HET_tm1b (Coronal)
Ezh2_HET_tm1a (Coronal)
Ezr_HET_tm2a (Coronal)
Fads3_HOM_tm1b (Coronal)
Fahd2a_HOM_tm1a (Coronal)
Fam104a_HOM_tm2a (Coronal)
Fam107b_HOM_tm1a (Coronal)
Fam122c_HEMI_tm1b (Coronal)
Fam160a1_HET_tm1b (Coronal)
Fam163a_HOM_tm2b (Coronal)
Fam175b_HOM_tm1a (Coronal)
Fam212b_HOM_tm1a (Coronal)
Fam46c_HET_tm1b (Coronal)
Fam47e_HOM_tm1a (Coronal)
Fam69a_HOM_tm1a (Coronal)
Fam71b_HOM_tm1a (Coronal)
Fam92a_HOM_tm1b (Coronal)
Fam96a_HOM_tm2a (Coronal)
Fanci_HET_tm1a (Coronal)
Farp2_HOM_tm1a (Coronal)
Farp2_HOM_tm1a (Coronal)
Farsa_HET_tm2a (Coronal)
Fbf1_HOM_tm1a (Coronal)
Fbxl7_HOM_tm1a (Coronal)
Fbxo33_HOM_tm1b (Coronal)
Fbxo47_HET_tm1a (Coronal)
Fbxo47_HOM_tm1a (Coronal)
Fbxo7_HOM_tm1a (Coronal)
Fbxw26_HOM_tm1b (Coronal)
Fdft1_HET_tm1a (Coronal)
Fggy_HOM_tm1a (Coronal)
Fkbp3_HOM_tm2a (Coronal)
Fkbp7_HOM_tm2a (Coronal)
Fnip2_HOM_tm1a (Coronal)
Frmd7_HEMI_tm1a (Coronal)
Frrs1_HET_tm1a (Coronal)
Fryl_HET_tm1a (Coronal)
Fryl_HET_tm1b (Coronal)
Fto_HOM_tm1a (Coronal)
Fundc1_HEMI_tm1a (Coronal)
Fundc1_HEMI_tm1c (Coronal)
Fxyd3_HOM_tm1a (Coronal)
Fyn_HET_tm1a (Coronal)
Fzd6_HOM_tm2a (Coronal)
G3bp2_HET_tm1a (Coronal)
Galnt18_HOM_tm1b (Coronal)
Galntl5_HOM_tm1b (Coronal)
Gap43_HET_tm1a (Coronal)
Gatc_HET_tm1a (Coronal)
Gba2_HOM_tm1a (Coronal)
Gbe1_HET_tm1a (Coronal)
Gbf1_HET_tm1a (Coronal)
Gbp5_HOM_em1 (Coronal)
Gclc_HOM_tm1a (Coronal)
Gda_HOM_tm1a (Coronal)
Gdpd2_HEMI_tm1a (Coronal)
Gfm1_HET_tm1a (Coronal)
Gif_HOM_tm1a (Coronal)
Gimap6_HOM_tm1a (Coronal)
Git2_HOM_Gt (Coronal)
Gldc_HET_tm1a (Coronal)
Gle1_HET_tm1a (Coronal)
Glg1_HET_tm1a (Coronal)
Glo1_HOM_tm1a (Coronal)
Glt8d2_HET_tm1a (Coronal)
1_TBA
1_LV
1_Cg
1_Cg_Width
1_Cg_Height
1_gcc
1_gcc_Width_T
1_gcc_Width_B
1_gcc_Height
1_CPu
1_aca
1_Pir
1_M1
1_S2
2_TBA
2_LV
2_D3V
2_RSGc
2_RSGc_Width
2_RSGc_Height
2_cc
2_cc_Width
2_cc_Height
2_dhc
2_AM
2_Pir
2_ic
2_opt
2_mt
2_fi
2_Hb
2_HP
2_TILpy
2_DG
2_Mol
2_Rad
2_Or
2_M1
2_S2
3_TBA
3_4V
3_Pons
3_py
3_g7
3_CN
3_Lat
3_IntA
3_IGL
Increase
[0;0.1[
[0.1;0.2[
[0.2;0.25[
[0.25;0.3[
[0.3;+infinity[
Decrease
[0;0.1[
[0.1;0.2[
[0.2;0.25[
[0.25;0.3[
[0.3;+infinity[

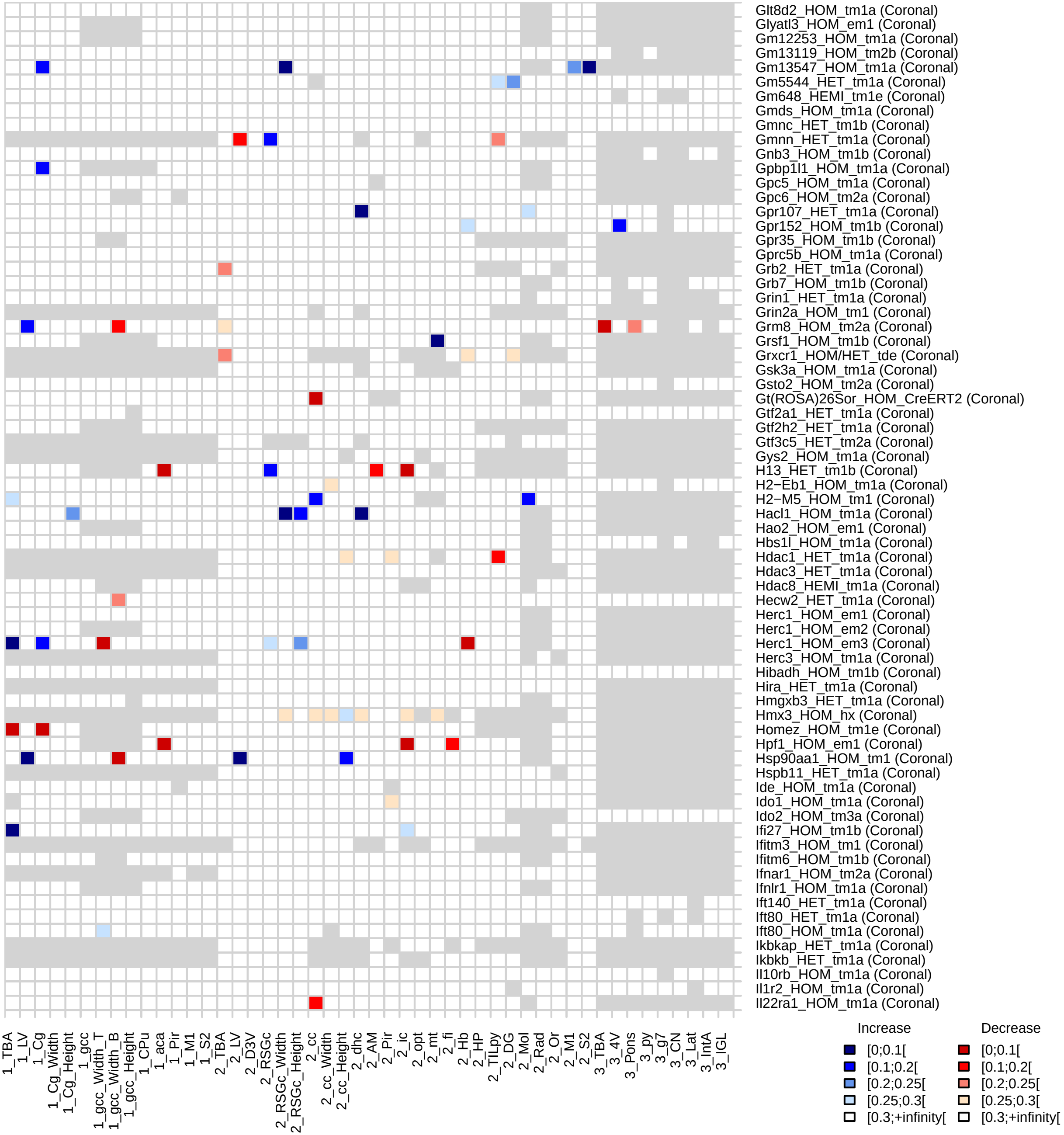
Glt8d2_HOM_tm1a (Coronal)
Glyatl3_HOM_em1 (Coronal)
Gm12253_HOM_tm1a (Coronal)
Gm13119_HOM_tm2b (Coronal)
Gm13547_HOM_tm1a (Coronal)
Gm5544_HET_tm1a (Coronal)
Gm648_HEMI_tm1e (Coronal)
Gmds_HOM_tm1a (Coronal)
Gmnc_HET_tm1b (Coronal)
Gmnn_HET_tm1a (Coronal)
Gnb3_HOM_tm1b (Coronal)
Gpbp1l1_HOM_tm1a (Coronal)
Gpc5_HOM_tm1a (Coronal)
Gpc6_HOM_tm2a (Coronal)
Gpr107_HET_tm1a (Coronal)
Gpr152_HOM_tm1b (Coronal)
Gpr35_HOM_tm1b (Coronal)
Gprc5b_HOM_tm1a (Coronal)
Grb2_HET_tm1a (Coronal)
Grb7_HOM_tm1b (Coronal)
Grin1_HET_tm1a (Coronal)
Grin2a_HOM_tm1 (Coronal)
Grm8_HOM_tm2a (Coronal)
Grsf1_HOM_tm1b (Coronal)
Grxcr1_HOM/HET_tde (Coronal)
Gsk3a_HOM_tm1a (Coronal)
Gsto2_HOM_tm2a (Coronal)
Gt(ROSA)26Sor_HOM_CreERT2 (Coronal)
Gtf2a1_HET_tm1a (Coronal)
Gtf2h2_HET_tm1a (Coronal)
Gtf3c5_HET_tm2a (Coronal)
Gys2_HOM_tm1a (Coronal)
H13_HET_tm1b (Coronal)
H2−Eb1_HOM_tm1a (Coronal)
H2−M5_HOM_tm1 (Coronal)
Hacl1_HOM_tm1a (Coronal)
Hao2_HOM_em1 (Coronal)
Hbs1l_HOM_tm1a (Coronal)
Hdac1_HET_tm1a (Coronal)
Hdac3_HET_tm1a (Coronal)
Hdac8_HEMI_tm1a (Coronal)
Hecw2_HET_tm1a (Coronal)
Herc1_HOM_em1 (Coronal)
Herc1_HOM_em2 (Coronal)
Herc1_HOM_em3 (Coronal)
Herc3_HOM_tm1a (Coronal)
Hibadh_HOM_tm1b (Coronal)
Hira_HET_tm1a (Coronal)
Hmgxb3_HET_tm1a (Coronal)
Hmx3_HOM_hx (Coronal)
Homez_HOM_tm1e (Coronal)
Hpf1_HOM_em1 (Coronal)
Hsp90aa1_HOM_tm1 (Coronal)
Hspb11_HET_tm1a (Coronal)
Ide_HOM_tm1a (Coronal)
Ido1_HOM_tm1a (Coronal)
Ido2_HOM_tm3a (Coronal)
Ifi27_HOM_tm1b (Coronal)
Ifitm3_HOM_tm1 (Coronal)
Ifitm6_HOM_tm1b (Coronal)
Ifnar1_HOM_tm2a (Coronal)
Ifnlr1_HOM_tm1a (Coronal)
Ift140_HET_tm1a (Coronal)
Ift80_HET_tm1a (Coronal)
Ift80_HOM_tm1a (Coronal)
Ikbkap_HET_tm1a (Coronal)
Ikbkb_HET_tm1a (Coronal)
Il10rb_HOM_tm1a (Coronal)
Il1r2_HOM_tm1a (Coronal)
Il22ra1_HOM_tm1a (Coronal)
1_TBA
1_LV
1_Cg
1_Cg_Width
1_Cg_Height
1_gcc
1_gcc_Width_T
1_gcc_Width_B
1_gcc_Height
1_CPu
1_aca
1_Pir
1_M1
1_S2
2_TBA
2_LV
2_D3V
2_RSGc
2_RSGc_Width
2_RSGc_Height
2_cc
2_cc_Width
2_cc_Height
2_dhc
2_AM
2_Pir
2_ic
2_opt
2_mt
2_fi
2_Hb
2_HP
2_TILpy
2_DG
2_Mol
2_Rad
2_Or
2_M1
2_S2
3_TBA
3_4V
3_Pons
3_py
3_g7
3_CN
3_Lat
3_IntA
3_IGL
Increase
[0;0.1[
[0.1;0.2[
[0.2;0.25[
[0.25;0.3[
[0.3;+infinity[
Decrease
[0;0.1[
[0.1;0.2[
[0.2;0.25[
[0.25;0.3[
[0.3;+infinity[

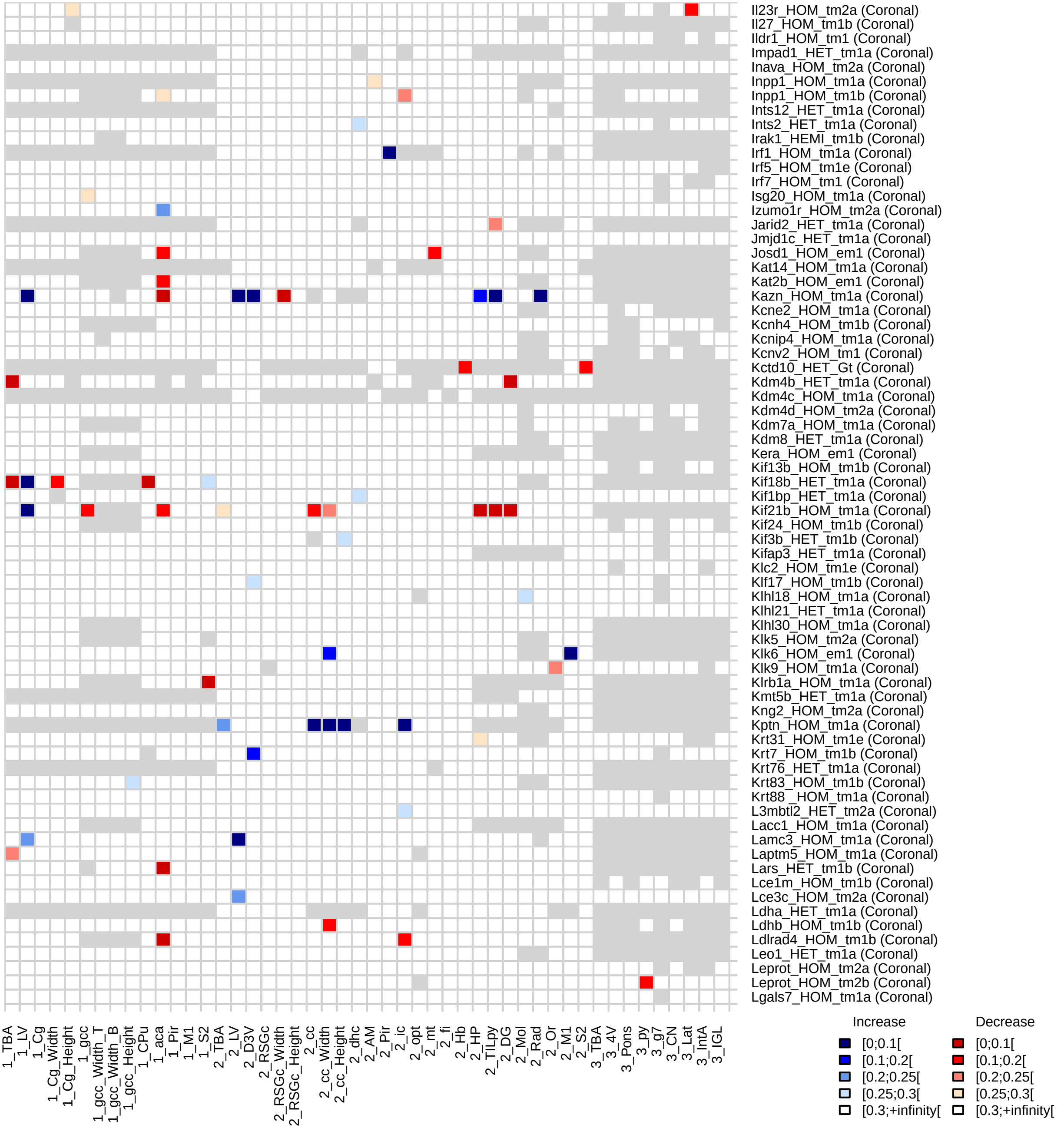
Il23r_HOM_tm2a (Coronal)
Il27_HOM_tm1b (Coronal)
Ildr1_HOM_tm1 (Coronal)
Impad1_HET_tm1a (Coronal)
Inava_HOM_tm2a (Coronal)
Inpp1_HOM_tm1a (Coronal)
Inpp1_HOM_tm1b (Coronal)
Ints12_HET_tm1a (Coronal)
Ints2_HET_tm1a (Coronal)
Irak1_HEMI_tm1b (Coronal)
Irf1_HOM_tm1a (Coronal)
Irf5_HOM_tm1e (Coronal)
Irf7_HOM_tm1 (Coronal)
Isg20_HOM_tm1a (Coronal)
Izumo1r_HOM_tm2a (Coronal)
Jarid2_HET_tm1a (Coronal)
Jmjd1c_HET_tm1a (Coronal)
Josd1_HOM_em1 (Coronal)
Kat14_HOM_tm1a (Coronal)
Kat2b_HOM_em1 (Coronal)
Kazn_HOM_tm1a (Coronal)
Kcne2_HOM_tm1a (Coronal)
Kcnh4_HOM_tm1b (Coronal)
Kcnip4_HOM_tm1a (Coronal)
Kcnv2_HOM_tm1 (Coronal)
Kctd10_HET_Gt (Coronal)
Kdm4b_HET_tm1a (Coronal)
Kdm4c_HOM_tm1a (Coronal)
Kdm4d_HOM_tm2a (Coronal)
Kdm7a_HOM_tm1a (Coronal)
Kdm8_HET_tm1a (Coronal)
Kera_HOM_em1 (Coronal)
Kif13b_HOM_tm1b (Coronal)
Kif18b_HET_tm1a (Coronal)
Kif1bp_HET_tm1a (Coronal)
Kif21b_HOM_tm1a (Coronal)
Kif24_HOM_tm1b (Coronal)
Kif3b_HET_tm1b (Coronal)
Kifap3_HET_tm1a (Coronal)
Klc2_HOM_tm1e (Coronal)
Klf17_HOM_tm1b (Coronal)
Klhl18_HOM_tm1a (Coronal)
Klhl21_HET_tm1a (Coronal)
Klhl30_HOM_tm1a (Coronal)
Klk5_HOM_tm2a (Coronal)
Klk6_HOM_em1 (Coronal)
Klk9_HOM_tm1a (Coronal)
Klrb1a_HOM_tm1a (Coronal)
Kmt5b_HET_tm1a (Coronal)
Kng2_HOM_tm2a (Coronal)
Kptn_HOM_tm1a (Coronal)
Krt31_HOM_tm1e (Coronal)
Krt7_HOM_tm1b (Coronal)
Krt76_HET_tm1a (Coronal)
Krt83_HOM_tm1b (Coronal)
Krt88_HOM_tm1a (Coronal)
L3mbtl2_HET_tm2a (Coronal)
Lacc1_HOM_tm1a (Coronal)
Lamc3_HOM_tm1a (Coronal)
Laptm5_HOM_tm1a (Coronal)
Lars_HET_tm1b (Coronal)
Lce1m_HOM_tm1b (Coronal)
Lce3c_HOM_tm2a (Coronal)
Ldha_HET_tm1a (Coronal)
Ldhb_HOM_tm1b (Coronal)
Ldlrad4_HOM_tm1b (Coronal)
Leo1_HET_tm1a (Coronal)
Leprot_HOM_tm2a (Coronal)
Leprot_HOM_tm2b (Coronal)
Lgals7_HOM_tm1a (Coronal)
1_TBA
1_LV
1_Cg
1_Cg_Width
1_Cg_Height
1_gcc
1_gcc_Width_T
1_gcc_Width_B
1_gcc_Height
1_CPu
1_aca
1_Pir
1_M1
1_S2
2_TBA
2_LV
2_D3V
2_RSGc
2_RSGc_Width
2_RSGc_Height
2_cc
2_cc_Width
2_cc_Height
2_dhc
2_AM
2_Pir
2_ic
2_opt
2_mt
2_fi
2_Hb
2_HP
2_TILpy
2_DG
2_Mol
2_Rad
2_Or
2_M1
2_S2
3_TBA
3_4V
3_Pons
3_py
3_g7
3_CN
3_Lat
3_IntA
3_IGL
Increase
[0;0.1[
[0.1;0.2[
[0.2;0.25[
[0.25;0.3[
[0.3;+infinity[
Decrease
[0;0.1[
[0.1;0.2[
[0.2;0.25[
[0.25;0.3[
[0.3;+infinity[

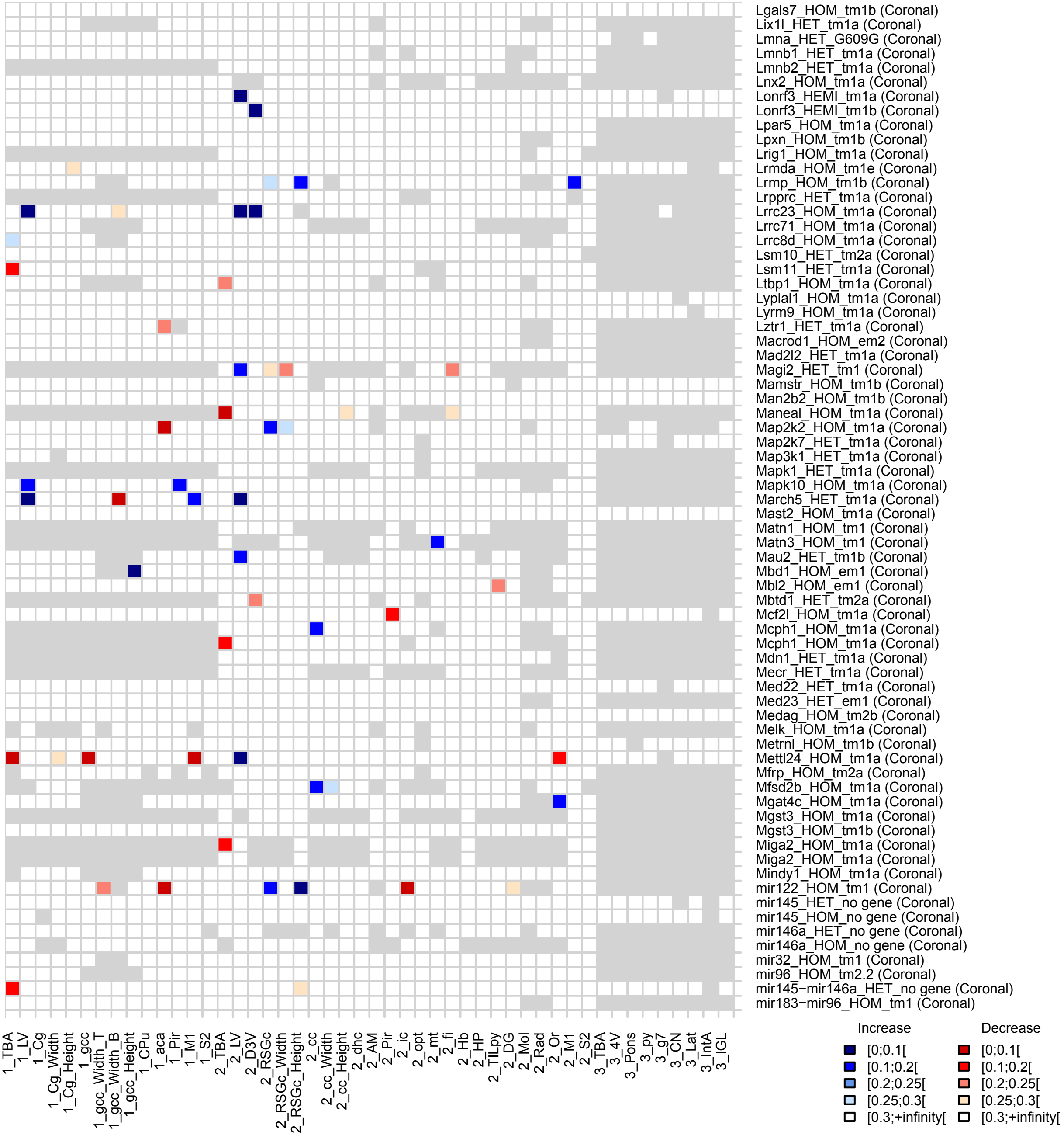

Lgals7_HOM_tm1b (Coronal)
Lix1l_HET_tm1a (Coronal)
Lmna_HET_G609G (Coronal)
Lmnb1_HET_tm1a (Coronal)
Lmnb2_HET_tm1a (Coronal)
Lnx2_HOM_tm1a (Coronal)
Lonrf3_HEMI_tm1a (Coronal)
Lonrf3_HEMI_tm1b (Coronal)
Lpar5_HOM_tm1a (Coronal)
Lpxn_HOM_tm1b (Coronal)
Lrig1_HOM_tm1a (Coronal)
Lrmda_HOM_tm1e (Coronal)
Lrmp_HOM_tm1b (Coronal)
Lrpprc_HET_tm1a (Coronal)
Lrrc23_HOM_tm1a (Coronal)
Lrrc71_HOM_tm1a (Coronal)
Lrrc8d_HOM_tm1a (Coronal)
Lsm10_HET_tm2a (Coronal)
Lsm11_HET_tm1a (Coronal)
Ltbp1_HOM_tm1a (Coronal)
Lyplal1_HOM_tm1a (Coronal)
Lyrm9_HOM_tm1a (Coronal)
Lztr1_HET_tm1a (Coronal)
Macrod1_HOM_em2 (Coronal)
Mad2l2_HET_tm1a (Coronal)
Magi2_HET_tm1 (Coronal)
Mamstr_HOM_tm1b (Coronal)
Man2b2_HOM_tm1b (Coronal)
Maneal_HOM_tm1a (Coronal)
Map2k2_HOM_tm1a (Coronal)
Map2k7_HET_tm1a (Coronal)
Map3k1_HET_tm1a (Coronal)
Mapk1_HET_tm1a (Coronal)
Mapk10_HOM_tm1a (Coronal)
March5_HET_tm1a (Coronal)
Mast2_HOM_tm1a (Coronal)
Matn1_HOM_tm1 (Coronal)
Matn3_HOM_tm1 (Coronal)
Mau2_HET_tm1b (Coronal)
Mbd1_HOM_em1 (Coronal)
Mbl2_HOM_em1 (Coronal)
Mbtd1_HET_tm2a (Coronal)
Mcf2l_HOM_tm1a (Coronal)
Mcph1_HOM_tm1a (Coronal)
Mcph1_HOM_tm1a (Coronal)
Mdn1_HET_tm1a (Coronal)
Mecr_HET_tm1a (Coronal)
Med22_HET_tm1a (Coronal)
Med23_HET_em1 (Coronal)
Medag_HOM_tm2b (Coronal)
Melk_HOM_tm1a (Coronal)
Metrnl_HOM_tm1b (Coronal)
Mettl24_HOM_tm1a (Coronal)
Mfrp_HOM_tm2a (Coronal)
Mfsd2b_HOM_tm1a (Coronal)
Mgat4c_HOM_tm1a (Coronal)
Mgst3_HOM_tm1a (Coronal)
Mgst3_HOM_tm1b (Coronal)
Miga2_HOM_tm1a (Coronal)
Miga2_HOM_tm1a (Coronal)
Mindy1_HOM_tm1a (Coronal)
mir122_HOM_tm1 (Coronal)
mir145_HET_no gene (Coronal)
mir145_HOM_no gene (Coronal)
mir146a_HET_no gene (Coronal)
mir146a_HOM_no gene (Coronal)
mir32_HOM_tm1 (Coronal)
mir96_HOM_tm2.2 (Coronal)
mir145−mir146a_HET_no gene (Coronal)
mir183−mir96_HOM_tm1 (Coronal)
1_TBA
1_LV
1_Cg
1_Cg_Width
1_Cg_Height
1_gcc
1_gcc_Width_T
1_gcc_Width_B
1_gcc_Height
1_CPu
1_aca
1_Pir
1_M1
1_S2
2_TBA
2_LV
2_D3V
2_RSGc
2_RSGc_Width
2_RSGc_Height
2_cc
2_cc_Width
2_cc_Height
2_dhc
2_AM
2_Pir
2_ic
2_opt
2_mt
2_fi
2_Hb
2_HP
2_TILpy
2_DG
2_Mol
2_Rad
2_Or
2_M1
2_S2
3_TBA
3_4V
3_Pons
3_py
3_g7
3_CN
3_Lat
3_IntA
3_IGL
Increase
[0;0.1[
[0.1;0.2[
[0.2;0.25[
[0.25;0.3[
[0.3;+infinity[
Decrease
[0;0.1[
[0.1;0.2[
[0.2;0.25[
[0.25;0.3[
[0.3;+infinity[

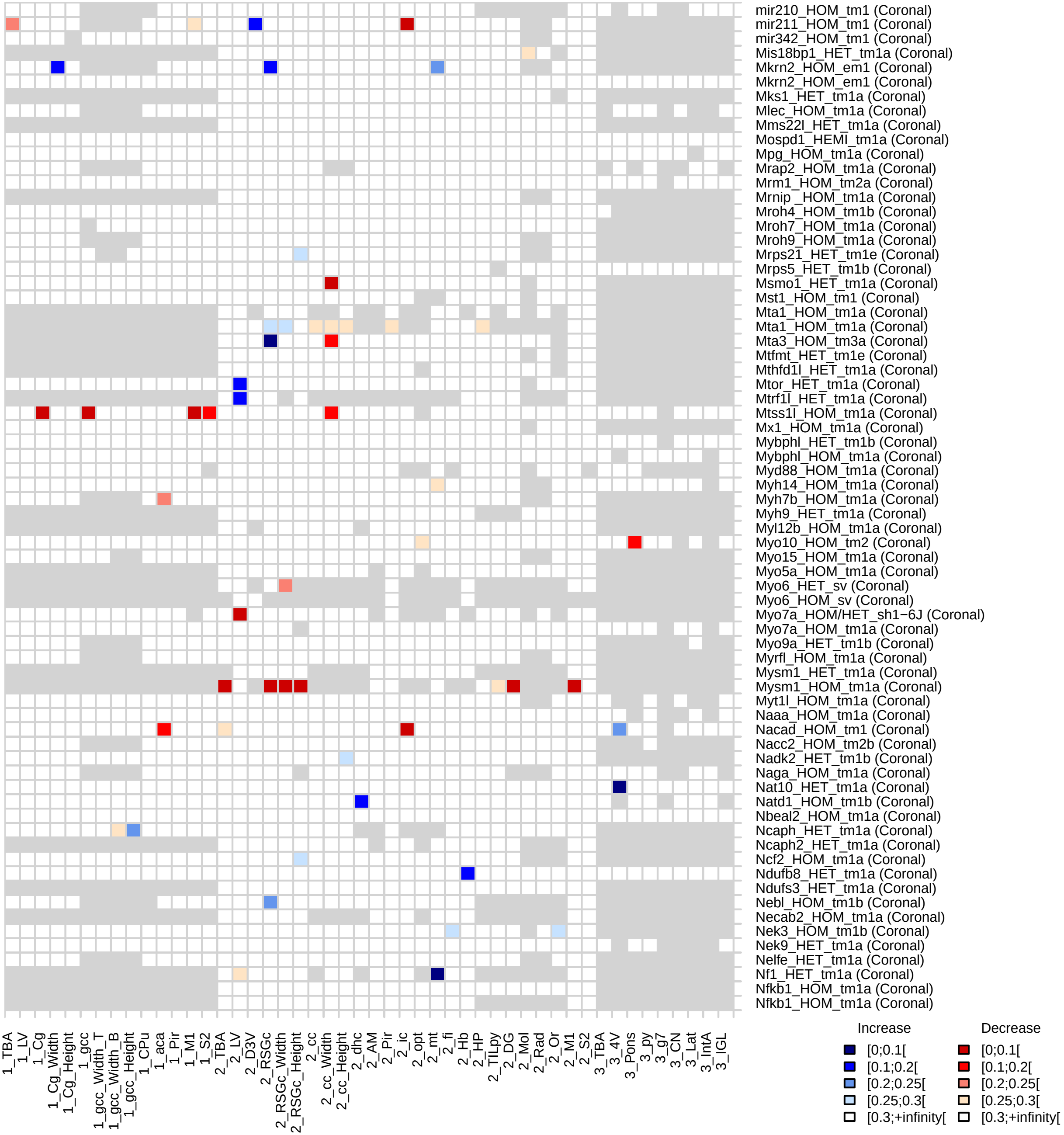

mir210_HOM_tm1 (Coronal)
mir211_HOM_tm1 (Coronal)
mir342_HOM_tm1 (Coronal)
Mis18bp1_HET_tm1a (Coronal)
Mkrn2_HOM_em1 (Coronal)
Mkrn2_HOM_em1 (Coronal)
Mks1_HET_tm1a (Coronal)
Mlec_HOM_tm1a (Coronal)
Mms22l_HET_tm1a (Coronal)
Mospd1_HEMI_tm1a (Coronal)
Mpg_HOM_tm1a (Coronal)
Mrap2_HOM_tm1a (Coronal)
Mrm1_HOM_tm2a (Coronal)
Mrnip_HOM_tm1a (Coronal)
Mroh4_HOM_tm1b (Coronal)
Mroh7_HOM_tm1a (Coronal)
Mroh9_HOM_tm1a (Coronal)
Mrps21_HET_tm1e (Coronal)
Mrps5_HET_tm1b (Coronal)
Msmo1_HET_tm1a (Coronal)
Mst1_HOM_tm1 (Coronal)
Mta1_HOM_tm1a (Coronal)
Mta1_HOM_tm1a (Coronal)
Mta3_HOM_tm3a (Coronal)
Mtfmt_HET_tm1e (Coronal)
Mthfd1l_HET_tm1a (Coronal)
Mtor_HET_tm1a (Coronal)
Mtrf1l_HET_tm1a (Coronal)
Mtss1l_HOM_tm1a (Coronal)
Mx1_HOM_tm1a (Coronal)
Mybphl_HET_tm1b (Coronal)
Mybphl_HOM_tm1a (Coronal)
Myd88_HOM_tm1a (Coronal)
Myh14_HOM_tm1a (Coronal)
Myh7b_HOM_tm1a (Coronal)
Myh9_HET_tm1a (Coronal)
Myl12b_HOM_tm1a (Coronal)
Myo10_HOM_tm2 (Coronal)
Myo15_HOM_tm1a (Coronal)
Myo5a_HOM_tm1a (Coronal)
Myo6_HET_sv (Coronal)
Myo6_HOM_sv (Coronal)
Myo7a_HOM/HET_sh1−6J (Coronal)
Myo7a_HOM_tm1a (Coronal)
Myo9a_HET_tm1b (Coronal)
Myrfl_HOM_tm1a (Coronal)
Mysm1_HET_tm1a (Coronal)
Mysm1_HOM_tm1a (Coronal)
Myt1l_HOM_tm1a (Coronal)
Naaa_HOM_tm1a (Coronal)
Nacad_HOM_tm1 (Coronal)
Nacc2_HOM_tm2b (Coronal)
Nadk2_HET_tm1b (Coronal)
Naga_HOM_tm1a (Coronal)
Nat10_HET_tm1a (Coronal)
Natd1_HOM_tm1b (Coronal)
Nbeal2_HOM_tm1a (Coronal)
Ncaph_HET_tm1a (Coronal)
Ncaph2_HET_tm1a (Coronal)
Ncf2_HOM_tm1a (Coronal)
Ndufb8_HET_tm1a (Coronal)
Ndufs3_HET_tm1a (Coronal)
Nebl_HOM_tm1b (Coronal)
Necab2_HOM_tm1a (Coronal)
Nek3_HOM_tm1b (Coronal)
Nek9_HET_tm1a (Coronal)
Nelfe_HET_tm1a (Coronal)
Nf1_HET_tm1a (Coronal)
Nfkb1_HOM_tm1a (Coronal)
Nfkb1_HOM_tm1a (Coronal)
1_TBA
1_LV
1_Cg
1_Cg_Width
1_Cg_Height
1_gcc
1_gcc_Width_T
1_gcc_Width_B
1_gcc_Height
1_CPu
1_aca
1_Pir
1_M1
1_S2
2_TBA
2_LV
2_D3V
2_RSGc
2_RSGc_Width
2_RSGc_Height
2_cc
2_cc_Width
2_cc_Height
2_dhc
2_AM
2_Pir
2_ic
2_opt
2_mt
2_fi
2_Hb
2_HP
2_TILpy
2_DG
2_Mol
2_Rad
2_Or
2_M1
2_S2
3_TBA
3_4V
3_Pons
3_py
3_g7
3_CN
3_Lat
3_IntA
3_IGL
Increase
[0;0.1[
[0.1;0.2[
[0.2;0.25[
[0.25;0.3[
[0.3;+infinity[
Decrease
[0;0.1[
[0.1;0.2[
[0.2;0.25[
[0.25;0.3[
[0.3;+infinity[

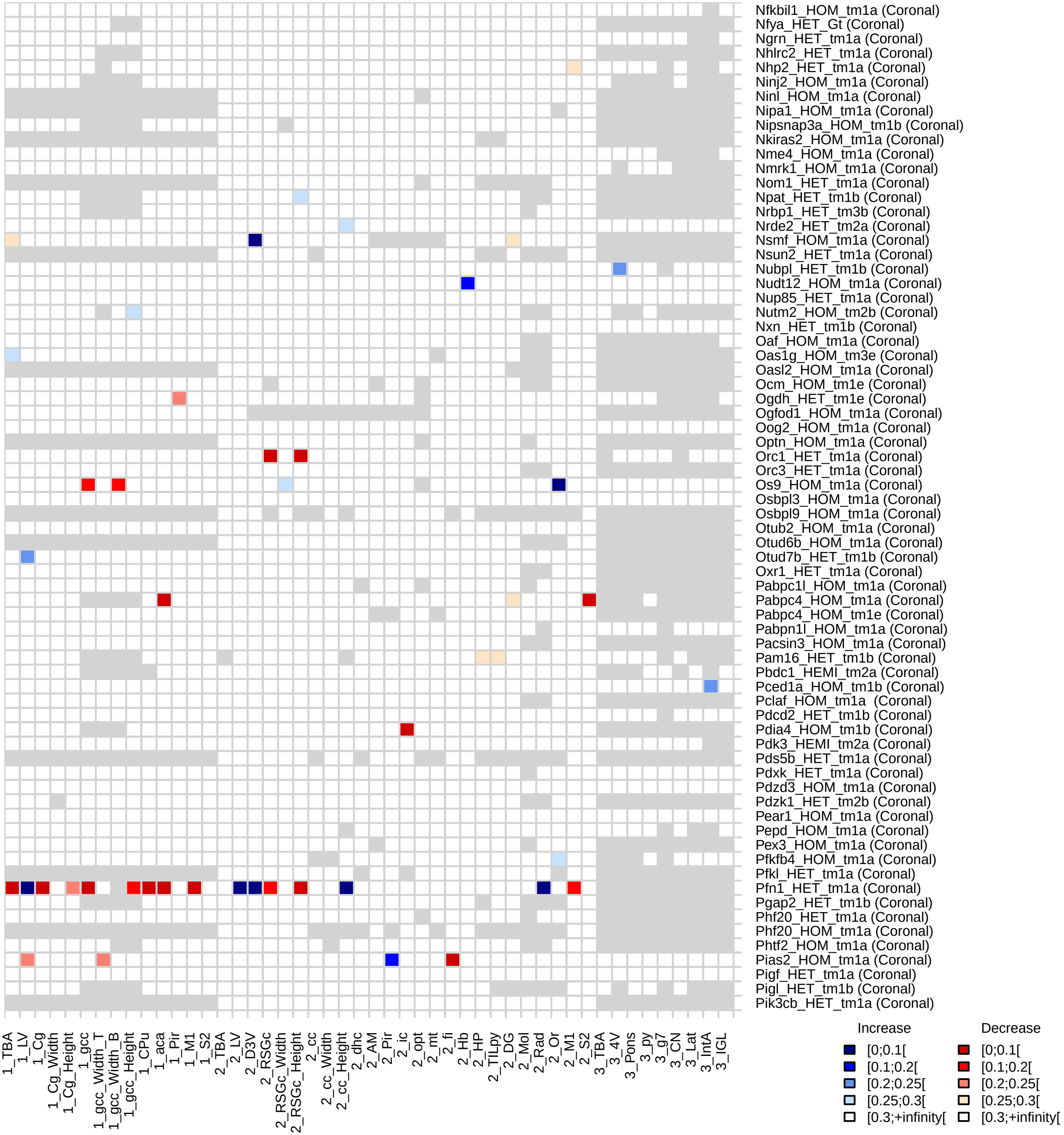
Nfkbil1_HOM_tm1a (Coronal)
Nfya_HET_Gt (Coronal)
Ngrn_HET_tm1a (Coronal)
Nhlrc2_HET_tm1a (Coronal)
Nhp2_HET_tm1a (Coronal)
Ninj2_HOM_tm1a (Coronal)
Ninl_HOM_tm1a (Coronal)
Nipa1_HOM_tm1a (Coronal)
Nipsnap3a_HOM_tm1b (Coronal)
Nkiras2_HOM_tm1a (Coronal)
Nme4_HOM_tm1a (Coronal)
Nmrk1_HOM_tm1a (Coronal)
Nom1_HET_tm1a (Coronal)
Npat_HET_tm1b (Coronal)
Nrbp1_HET_tm3b (Coronal)
Nrde2_HET_tm2a (Coronal)
Nsmf_HOM_tm1a (Coronal)
Nsun2_HET_tm1a (Coronal)
Nubpl_HET_tm1b (Coronal)
Nudt12_HOM_tm1a (Coronal)
Nup85_HET_tm1a (Coronal)
Nutm2_HOM_tm2b (Coronal)
Nxn_HET_tm1b (Coronal)
Oaf_HOM_tm1a (Coronal)
Oas1g_HOM_tm3e (Coronal)
Oasl2_HOM_tm1a (Coronal)
Ocm_HOM_tm1e (Coronal)
Ogdh_HET_tm1e (Coronal)
Ogfod1_HOM_tm1a (Coronal)
Oog2_HOM_tm1a (Coronal)
Optn_HOM_tm1a (Coronal)
Orc1_HET_tm1a (Coronal)
Orc3_HET_tm1a (Coronal)
Os9_HOM_tm1a (Coronal)
Osbpl3_HOM_tm1a (Coronal)
Osbpl9_HOM_tm1a (Coronal)
Otub2_HOM_tm1a (Coronal)
Otud6b_HOM_tm1a (Coronal)
Otud7b_HET_tm1b (Coronal)
Oxr1_HET_tm1a (Coronal)
Pabpc1l_HOM_tm1a (Coronal)
Pabpc4_HOM_tm1a (Coronal)
Pabpc4_HOM_tm1e (Coronal)
Pabpn1l_HOM_tm1a (Coronal)
Pacsin3_HOM_tm1a (Coronal)
Pam16_HET_tm1b (Coronal)
Pbdc1_HEMI_tm2a (Coronal)
Pced1a_HOM_tm1b (Coronal)
Pclaf_HOM_tm1a (Coronal)
Pdcd2_HET_tm1b (Coronal)
Pdia4_HOM_tm1b (Coronal)
Pdk3_HEMI_tm2a (Coronal)
Pds5b_HET_tm1a (Coronal)
Pdxk_HET_tm1a (Coronal)
Pdzd3_HOM_tm1a (Coronal)
Pdzk1_HET_tm2b (Coronal)
Pear1_HOM_tm1a (Coronal)
Pepd_HOM_tm1a (Coronal)
Pex3_HOM_tm1a (Coronal)
Pfkfb4_HOM_tm1a (Coronal)
Pfkl_HET_tm1a (Coronal)
Pfn1_HET_tm1a (Coronal)
Pgap2_HET_tm1b (Coronal)
Phf20_HET_tm1a (Coronal)
Phf20_HOM_tm1a (Coronal)
Phtf2_HOM_tm1a (Coronal)
Pias2_HOM_tm1a (Coronal)
Pigf_HET_tm1a (Coronal)
Pigl_HET_tm1b (Coronal)
Pik3cb_HET_tm1a (Coronal)
1_TBA
1_LV
1_Cg
1_Cg_Width
1_Cg_Height
1_gcc
1_gcc_Width_T
1_gcc_Width_B
1_gcc_Height
1_CPu
1_aca
1_Pir
1_M1
1_S2
2_TBA
2_LV
2_D3V
2_RSGc
2_RSGc_Width
2_RSGc_Height
2_cc
2_cc_Width
2_cc_Height
2_dhc
2_AM
2_Pir
2_ic
2_opt
2_mt
2_fi
2_Hb
2_HP
2_TILpy
2_DG
2_Mol
2_Rad
2_Or
2_M1
2_S2
3_TBA
3_4V
3_Pons
3_py
3_g7
3_CN
3_Lat
3_IntA
3_IGL
Increase
[0;0.1[
[0.1;0.2[
[0.2;0.25[
[0.25;0.3[
[0.3;+infinity[
Decrease
[0;0.1[
[0.1;0.2[
[0.2;0.25[
[0.25;0.3[
[0.3;+infinity[

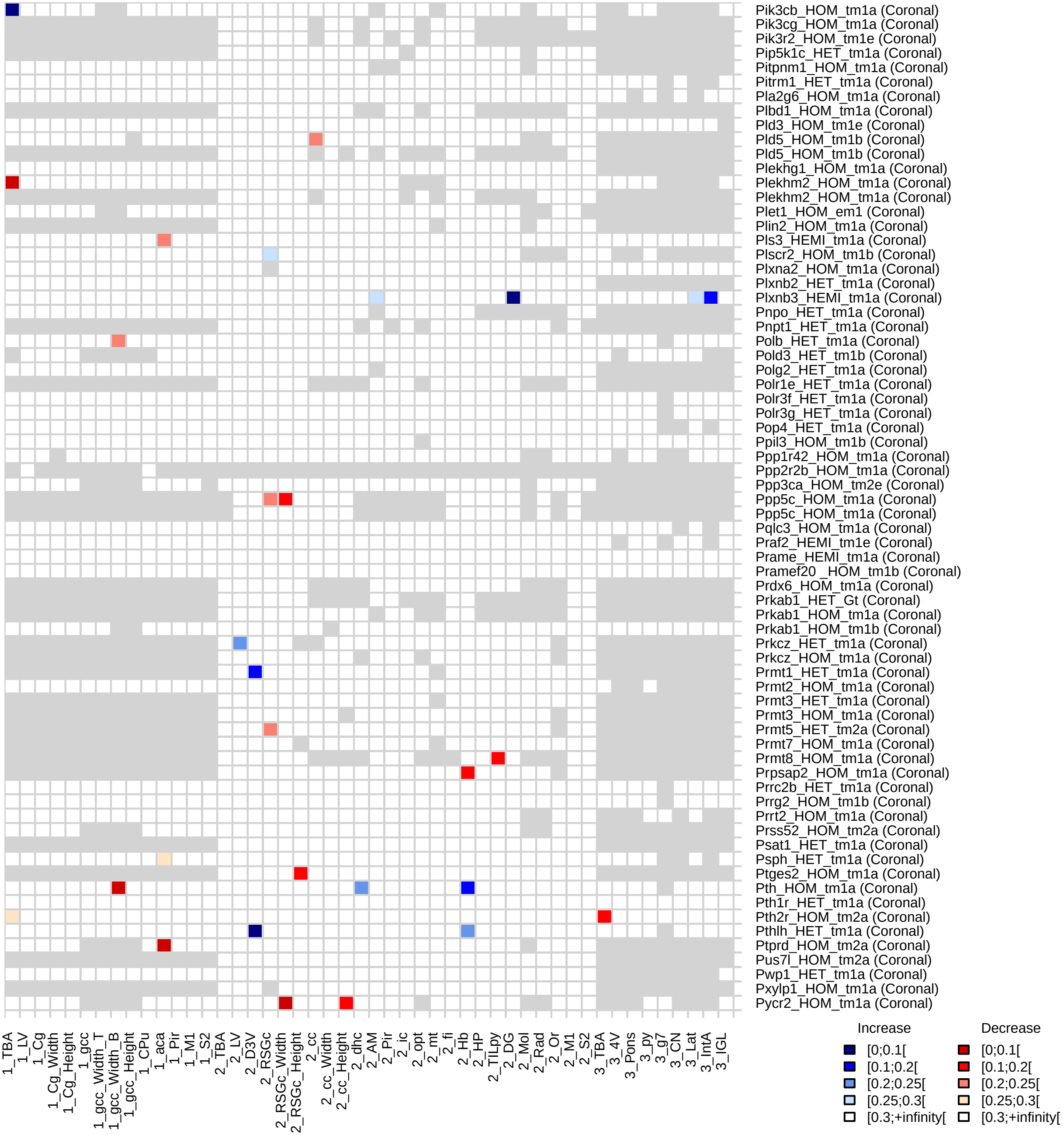
Pik3cb_HOM_tm1a (Coronal)
Pik3cg_HOM_tm1a (Coronal)
Pik3r2_HOM_tm1e (Coronal)
Pip5k1c_HET_tm1a (Coronal)
Pitpnm1_HOM_tm1a (Coronal)
Pitrm1_HET_tm1a (Coronal)
Pla2g6_HOM_tm1a (Coronal)
Plbd1_HOM_tm1a (Coronal)
Pld3_HOM_tm1e (Coronal)
Pld5_HOM_tm1b (Coronal)
Pld5_HOM_tm1b (Coronal)
Plekhg1_HOM_tm1a (Coronal)
Plekhm2_HOM_tm1a (Coronal)
Plekhm2_HOM_tm1a (Coronal)
Plet1_HOM_em1 (Coronal)
Plin2_HOM_tm1a (Coronal)
Pls3_HEMI_tm1a (Coronal)
Plscr2_HOM_tm1b (Coronal)
Plxna2_HOM_tm1a (Coronal)
Plxnb2_HET_tm1a (Coronal)
Plxnb3_HEMI_tm1a (Coronal)
Pnpo_HET_tm1a (Coronal)
Pnpt1_HET_tm1a (Coronal)
Polb_HET_tm1a (Coronal)
Pold3_HET_tm1b (Coronal)
Polg2_HET_tm1a (Coronal)
Polr1e_HET_tm1a (Coronal)
Polr3f_HET_tm1a (Coronal)
Polr3g_HET_tm1a (Coronal)
Pop4_HET_tm1a (Coronal)
Ppil3_HOM_tm1b (Coronal)
Ppp1r42_HOM_tm1a (Coronal)
Ppp2r2b_HOM_tm1a (Coronal)
Ppp3ca_HOM_tm2e (Coronal)
Ppp5c_HOM_tm1a (Coronal)
Ppp5c_HOM_tm1a (Coronal)
Pqlc3_HOM_tm1a (Coronal)
Praf2_HEMI_tm1e (Coronal)
Prame_HEMI_tm1a (Coronal)
Pramef20 _HOM_tm1b (Coronal)
Prdx6_HOM_tm1a (Coronal)
Prkab1_HET_Gt (Coronal)
Prkab1_HOM_tm1a (Coronal)
Prkab1_HOM_tm1b (Coronal)
Prkcz_HET_tm1a (Coronal)
Prkcz_HOM_tm1a (Coronal)
Prmt1_HET_tm1a (Coronal)
Prmt2_HOM_tm1a (Coronal)
Prmt3_HET_tm1a (Coronal)
Prmt3_HOM_tm1a (Coronal)
Prmt5_HET_tm2a (Coronal)
Prmt7_HOM_tm1a (Coronal)
Prmt8_HOM_tm1a (Coronal)
Prpsap2_HOM_tm1a (Coronal)
Prrc2b_HET_tm1a (Coronal)
Prrg2_HOM_tm1b (Coronal)
Prrt2_HOM_tm1a (Coronal)
Prss52_HOM_tm2a (Coronal)
Psat1_HET_tm1a (Coronal)
Psph_HET_tm1a (Coronal)
Ptges2_HOM_tm1a (Coronal)
Pth_HOM_tm1a (Coronal)
Pth1r_HET_tm1a (Coronal)
Pth2r_HOM_tm2a (Coronal)
Pthlh_HET_tm1a (Coronal)
Ptprd_HOM_tm2a (Coronal)
Pus7l_HOM_tm2a (Coronal)
Pwp1_HET_tm1a (Coronal)
Pxylp1_HOM_tm1a (Coronal)
Pycr2_HOM_tm1a (Coronal)
1_TBA
1_LV
1_Cg
1_Cg_Width
1_Cg_Height
1_gcc
1_gcc_Width_T
1_gcc_Width_B
1_gcc_Height
1_CPu
1_aca
1_Pir
1_M1
1_S2
2_TBA
2_LV
2_D3V
2_RSGc
2_RSGc_Width
2_RSGc_Height
2_cc
2_cc_Width
2_cc_Height
2_dhc
2_AM
2_Pir
2_ic
2_opt
2_mt
2_fi
2_Hb
2_HP
2_TILpy
2_DG
2_Mol
2_Rad
2_Or
2_M1
2_S2
3_TBA
3_4V
3_Pons
3_py
3_g7
3_CN
3_Lat
3_IntA
3_IGL
Increase
[0;0.1[
[0.1;0.2[
[0.2;0.25[
[0.25;0.3[
[0.3;+infinity[
Decrease
[0;0.1[
[0.1;0.2[
[0.2;0.25[
[0.25;0.3[
[0.3;+infinity[

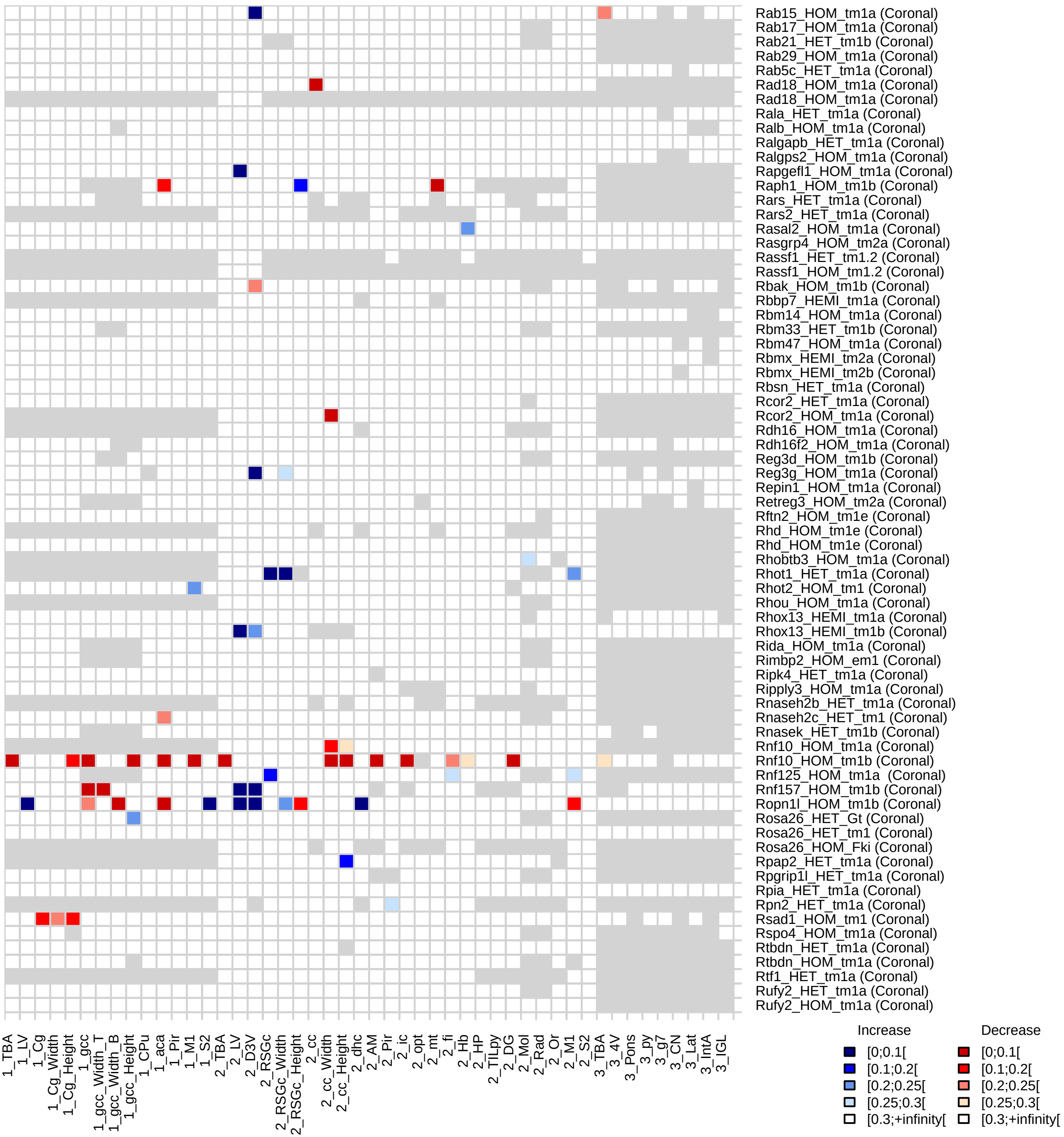

Rab15_HOM_tm1a (Coronal)
Rab17_HOM_tm1a (Coronal)
Rab21_HET_tm1b (Coronal)
Rab29_HOM_tm1a (Coronal)
Rab5c_HET_tm1a (Coronal)
Rad18_HOM_tm1a (Coronal)
Rad18_HOM_tm1a (Coronal)
Rala_HET_tm1a (Coronal)
Ralb_HOM_tm1a (Coronal)
Ralgapb_HET_tm1a (Coronal)
Ralgps2_HOM_tm1a (Coronal)
Rapgefl1_HOM_tm1a (Coronal)
Raph1_HOM_tm1b (Coronal)
Rars_HET_tm1a (Coronal)
Rars2_HET_tm1a (Coronal)
Rasal2_HOM_tm1a (Coronal)
Rasgrp4_HOM_tm2a (Coronal)
Rassf1_HET_tm1.2 (Coronal)
Rassf1_HOM_tm1.2 (Coronal)
Rbak_HOM_tm1b (Coronal)
Rbbp7_HEMI_tm1a (Coronal)
Rbm14_HOM_tm1a (Coronal)
Rbm33_HET_tm1b (Coronal)
Rbm47_HOM_tm1a (Coronal)
Rbmx_HEMI_tm2a (Coronal)
Rbmx_HEMI_tm2b (Coronal)
Rbsn_HET_tm1a (Coronal)
Rcor2_HET_tm1a (Coronal)
Rcor2_HOM_tm1a (Coronal)
Rdh16_HOM_tm1a (Coronal)
Rdh16f2_HOM_tm1a (Coronal)
Reg3d_HOM_tm1b (Coronal)
Reg3g_HOM_tm1a (Coronal)
Repin1_HOM_tm1a (Coronal)
Retreg3_HOM_tm2a (Coronal)
Rftn2_HOM_tm1e (Coronal)
Rhd_HOM_tm1e (Coronal)
Rhd_HOM_tm1e (Coronal)
Rhobtb3_HOM_tm1a (Coronal)
Rhot1_HET_tm1a (Coronal)
Rhot2_HOM_tm1 (Coronal)
Rhou_HOM_tm1a (Coronal)
Rhox13_HEMI_tm1a (Coronal)
Rhox13_HEMI_tm1b (Coronal)
Rida_HOM_tm1a (Coronal)
Rimbp2_HOM_em1 (Coronal)
Ripk4_HET_tm1a (Coronal)
Ripply3_HOM_tm1a (Coronal)
Rnaseh2b_HET_tm1a (Coronal)
Rnaseh2c_HET_tm1 (Coronal)
Rnasek_HET_tm1b (Coronal)
Rnf10_HOM_tm1a (Coronal)
Rnf10_HOM_tm1b (Coronal)
Rnf125_HOM_tm1a (Coronal)
Rnf157_HOM_tm1b (Coronal)
Ropn1l_HOM_tm1b (Coronal)
Rosa26_HET_Gt (Coronal)
Rosa26_HET_tm1 (Coronal)
Rosa26_HOM_Fki (Coronal)
Rpap2_HET_tm1a (Coronal)
Rpgrip1l_HET_tm1a (Coronal)
Rpia_HET_tm1a (Coronal)
Rpn2_HET_tm1a (Coronal)
Rsad1_HOM_tm1 (Coronal)
Rspo4_HOM_tm1a (Coronal)
Rtbdn_HET_tm1a (Coronal)
Rtbdn_HOM_tm1a (Coronal)
Rtf1_HET_tm1a (Coronal)
Rufy2_HET_tm1a (Coronal)
Rufy2_HOM_tm1a (Coronal)
1_TBA
1_LV
1_Cg
1_Cg_Width
1_Cg_Height
1_gcc
1_gcc_Width_T
1_gcc_Width_B
1_gcc_Height
1_CPu
1_aca
1_Pir
1_M1
1_S2
2_TBA
2_LV
2_D3V
2_RSGc
2_RSGc_Width
2_RSGc_Height
2_cc
2_cc_Width
2_cc_Height
2_dhc
2_AM
2_Pir
2_ic
2_opt
2_mt
2_fi
2_Hb
2_HP
2_TILpy
2_DG
2_Mol
2_Rad
2_Or
2_M1
2_S2
3_TBA
3_4V
3_Pons
3_py
3_g7
3_CN
3_Lat
3_IntA
3_IGL
Increase
[0;0.1[
[0.1;0.2[
[0.2;0.25[
[0.25;0.3[
[0.3;+infinity[
Decrease
[0;0.1[
[0.1;0.2[
[0.2;0.25[
[0.25;0.3[
[0.3;+infinity[

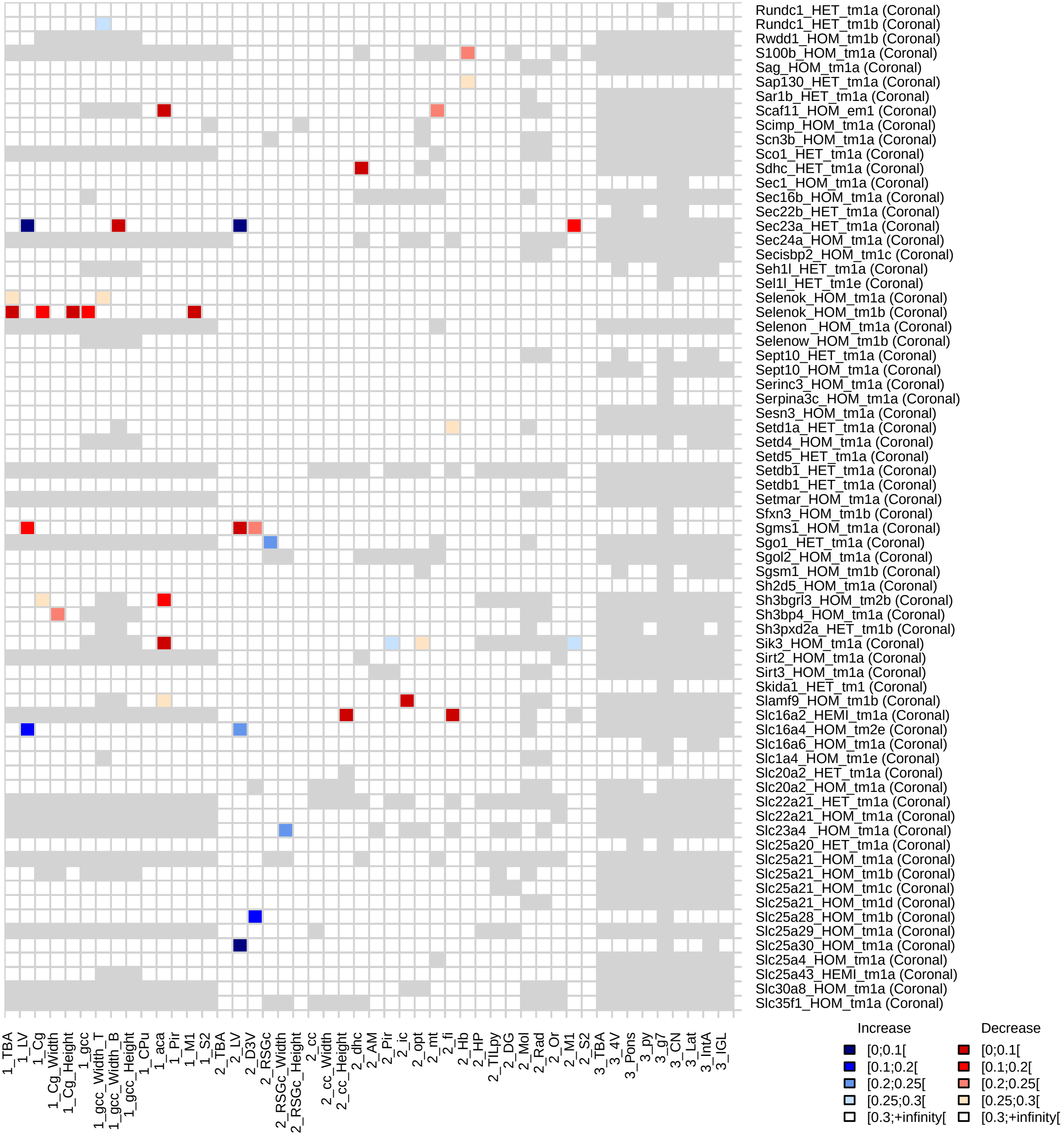
Rundc1_HET_tm1a (Coronal)
Rundc1_HET_tm1b (Coronal)
Rwdd1_HOM_tm1b (Coronal)
S100b_HOM_tm1a (Coronal)
Sag_HOM_tm1a (Coronal)
Sap130_HET_tm1a (Coronal)
Sar1b_HET_tm1a (Coronal)
Scaf11_HOM_em1 (Coronal)
Scimp_HOM_tm1a (Coronal)
Scn3b_HOM_tm1a (Coronal)
Sco1_HET_tm1a (Coronal)
Sdhc_HET_tm1a (Coronal)
Sec1_HOM_tm1a (Coronal)
Sec16b_HOM_tm1a (Coronal)
Sec22b_HET_tm1a (Coronal)
Sec23a_HET_tm1a (Coronal)
Sec24a_HOM_tm1a (Coronal)
Secisbp2_HOM_tm1c (Coronal)
Seh1l_HET_tm1a (Coronal)
Sel1l_HET_tm1e (Coronal)
Selenok_HOM_tm1a (Coronal)
Selenok_HOM_tm1b (Coronal)
Selenon _HOM_tm1a (Coronal)
Selenow_HOM_tm1b (Coronal)
Sept10_HET_tm1a (Coronal)
Sept10_HOM_tm1a (Coronal)
Serinc3_HOM_tm1a (Coronal)
Serpina3c_HOM_tm1a (Coronal)
Sesn3_HOM_tm1a (Coronal)
Setd1a_HET_tm1a (Coronal)
Setd4_HOM_tm1a (Coronal)
Setd5_HET_tm1a (Coronal)
Setdb1_HET_tm1a (Coronal)
Setdb1_HET_tm1a (Coronal)
Setmar_HOM_tm1a (Coronal)
Sfxn3_HOM_tm1b (Coronal)
Sgms1_HOM_tm1a (Coronal)
Sgo1_HET_tm1a (Coronal)
Sgol2_HOM_tm1a (Coronal)
Sgsm1_HOM_tm1b (Coronal)
Sh2d5_HOM_tm1a (Coronal)
Sh3bgrl3_HOM_tm2b (Coronal)
Sh3bp4_HOM_tm1a (Coronal)
Sh3pxd2a_HET_tm1b (Coronal)
Sik3_HOM_tm1a (Coronal)
Sirt2_HOM_tm1a (Coronal)
Sirt3_HOM_tm1a (Coronal)
Skida1_HET_tm1 (Coronal)
Slamf9_HOM_tm1b (Coronal)
Slc16a2_HEMI_tm1a (Coronal)
Slc16a4_HOM_tm2e (Coronal)
Slc16a6_HOM_tm1a (Coronal)
Slc1a4_HOM_tm1e (Coronal)
Slc20a2_HET_tm1a (Coronal)
Slc20a2_HOM_tm1a (Coronal)
Slc22a21_HET_tm1a (Coronal)
Slc22a21_HOM_tm1a (Coronal)
Slc23a4 _HOM_tm1a (Coronal)
Slc25a20_HET_tm1a (Coronal)
Slc25a21_HOM_tm1a (Coronal)
Slc25a21_HOM_tm1b (Coronal)
Slc25a21_HOM_tm1c (Coronal)
Slc25a21_HOM_tm1d (Coronal)
Slc25a28_HOM_tm1b (Coronal)
Slc25a29_HOM_tm1a (Coronal)
Slc25a30_HOM_tm1a (Coronal)
Slc25a4_HOM_tm1a (Coronal)
Slc25a43_HEMI_tm1a (Coronal)
Slc30a8_HOM_tm1a (Coronal)
Slc35f1_HOM_tm1a (Coronal)
1_TBA
1_LV
1_Cg
1_Cg_Width
1_Cg_Height
1_gcc
1_gcc_Width_T
1_gcc_Width_B
1_gcc_Height
1_CPu
1_aca
1_Pir
1_M1
1_S2
2_TBA
2_LV
2_D3V
2_RSGc
2_RSGc_Width
2_RSGc_Height
2_cc
2_cc_Width
2_cc_Height
2_dhc
2_AM
2_Pir
2_ic
2_opt
2_mt
2_fi
2_Hb
2_HP
2_TILpy
2_DG
2_Mol
2_Rad
2_Or
2_M1
2_S2
3_TBA
3_4V
3_Pons
3_py
3_g7
3_CN
3_Lat
3_IntA
3_IGL
Increase
[0;0.1[
[0.1;0.2[
[0.2;0.25[
[0.25;0.3[
[0.3;+infinity[
Decrease
[0;0.1[
[0.1;0.2[
[0.2;0.25[
[0.25;0.3[
[0.3;+infinity[

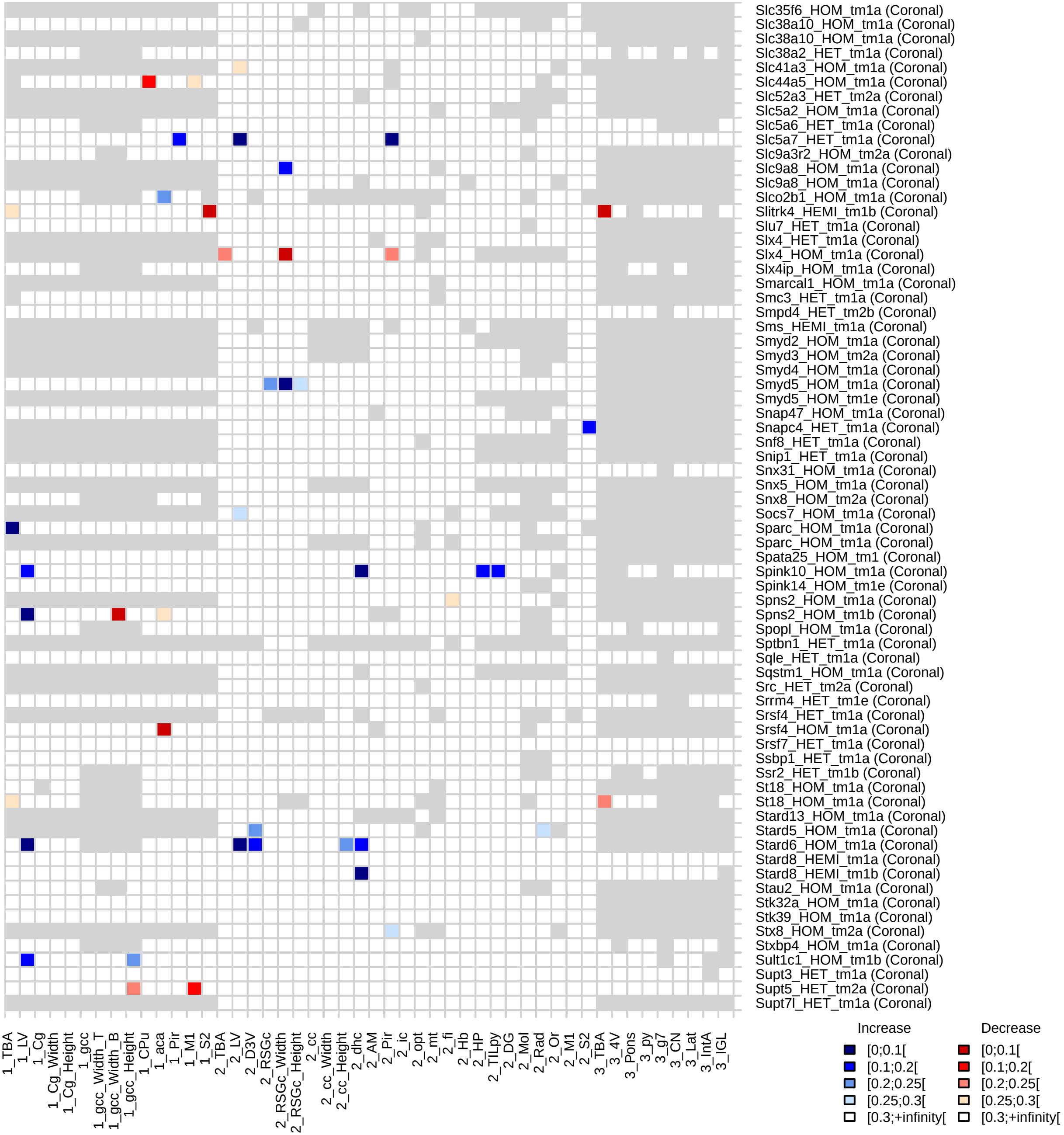
Slc35f6_HOM_tm1a (Coronal)
Slc38a10_HOM_tm1a (Coronal)
Slc38a10_HOM_tm1a (Coronal)
Slc38a2_HET_tm1a (Coronal)
Slc41a3_HOM_tm1a (Coronal)
Slc44a5_HOM_tm1a (Coronal)
Slc52a3_HET_tm2a (Coronal)
Slc5a2_HOM_tm1a (Coronal)
Slc5a6_HET_tm1a (Coronal)
Slc5a7_HET_tm1a (Coronal)
Slc9a3r2_HOM_tm2a (Coronal)
Slc9a8_HOM_tm1a (Coronal)
Slc9a8_HOM_tm1a (Coronal)
Slco2b1_HOM_tm1a (Coronal)
Slitrk4_HEMI_tm1b (Coronal)
Slu7_HET_tm1a (Coronal)
Slx4_HET_tm1a (Coronal)
Slx4_HOM_tm1a (Coronal)
Slx4ip_HOM_tm1a (Coronal)
Smarcal1_HOM_tm1a (Coronal)
Smc3_HET_tm1a (Coronal)
Smpd4_HET_tm2b (Coronal)
Sms_HEMI_tm1a (Coronal)
Smyd2_HOM_tm1a (Coronal)
Smyd3_HOM_tm2a (Coronal)
Smyd4_HOM_tm1a (Coronal)
Smyd5_HOM_tm1a (Coronal)
Smyd5_HOM_tm1e (Coronal)
Snap47_HOM_tm1a (Coronal)
Snapc4_HET_tm1a (Coronal)
Snf8_HET_tm1a (Coronal)
Snip1_HET_tm1a (Coronal)
Snx31_HOM_tm1a (Coronal)
Snx5_HOM_tm1a (Coronal)
Snx8_HOM_tm2a (Coronal)
Socs7_HOM_tm1a (Coronal)
Sparc_HOM_tm1a (Coronal)
Sparc_HOM_tm1a (Coronal)
Spata25_HOM_tm1 (Coronal)
Spink10_HOM_tm1a (Coronal)
Spink14_HOM_tm1e (Coronal)
Spns2_HOM_tm1a (Coronal)
Spns2_HOM_tm1b (Coronal)
Spopl_HOM_tm1a (Coronal)
Sptbn1_HET_tm1a (Coronal)
Sqle_HET_tm1a (Coronal)
Sqstm1_HOM_tm1a (Coronal)
Src_HET_tm2a (Coronal)
Srrm4_HET_tm1e (Coronal)
Srsf4_HET_tm1a (Coronal)
Srsf4_HOM_tm1a (Coronal)
Srsf7_HET_tm1a (Coronal)
Ssbp1_HET_tm1a (Coronal)
Ssr2_HET_tm1b (Coronal)
St18_HOM_tm1a (Coronal)
St18_HOM_tm1a (Coronal)
Stard13_HOM_tm1a (Coronal)
Stard5_HOM_tm1a (Coronal)
Stard6_HOM_tm1a (Coronal)
Stard8_HEMI_tm1a (Coronal)
Stard8_HEMI_tm1b (Coronal)
Stau2_HOM_tm1a (Coronal)
Stk32a_HOM_tm1a (Coronal)
Stk39_HOM_tm1a (Coronal)
Stx8_HOM_tm2a (Coronal)
Stxbp4_HOM_tm1a (Coronal)
Sult1c1_HOM_tm1b (Coronal)
Supt3_HET_tm1a (Coronal)
Supt5_HET_tm2a (Coronal)
Supt7l_HET_tm1a (Coronal)
1_TBA
1_LV
1_Cg
1_Cg_Width
1_Cg_Height
1_gcc
1_gcc_Width_T
1_gcc_Width_B
1_gcc_Height
1_CPu
1_aca
1_Pir
1_M1
1_S2
2_TBA
2_LV
2_D3V
2_RSGc
2_RSGc_Width
2_RSGc_Height
2_cc
2_cc_Width
2_cc_Height
2_dhc
2_AM
2_Pir
2_ic
2_opt
2_mt
2_fi
2_Hb
2_HP
2_TILpy
2_DG
2_Mol
2_Rad
2_Or
2_M1
2_S2
3_TBA
3_4V
3_Pons
3_py
3_g7
3_CN
3_Lat
3_IntA
3_IGL
Increase
[0;0.1[
[0.1;0.2[
[0.2;0.25[
[0.25;0.3[
[0.3;+infinity[
Decrease
[0;0.1[
[0.1;0.2[
[0.2;0.25[
[0.25;0.3[
[0.3;+infinity[

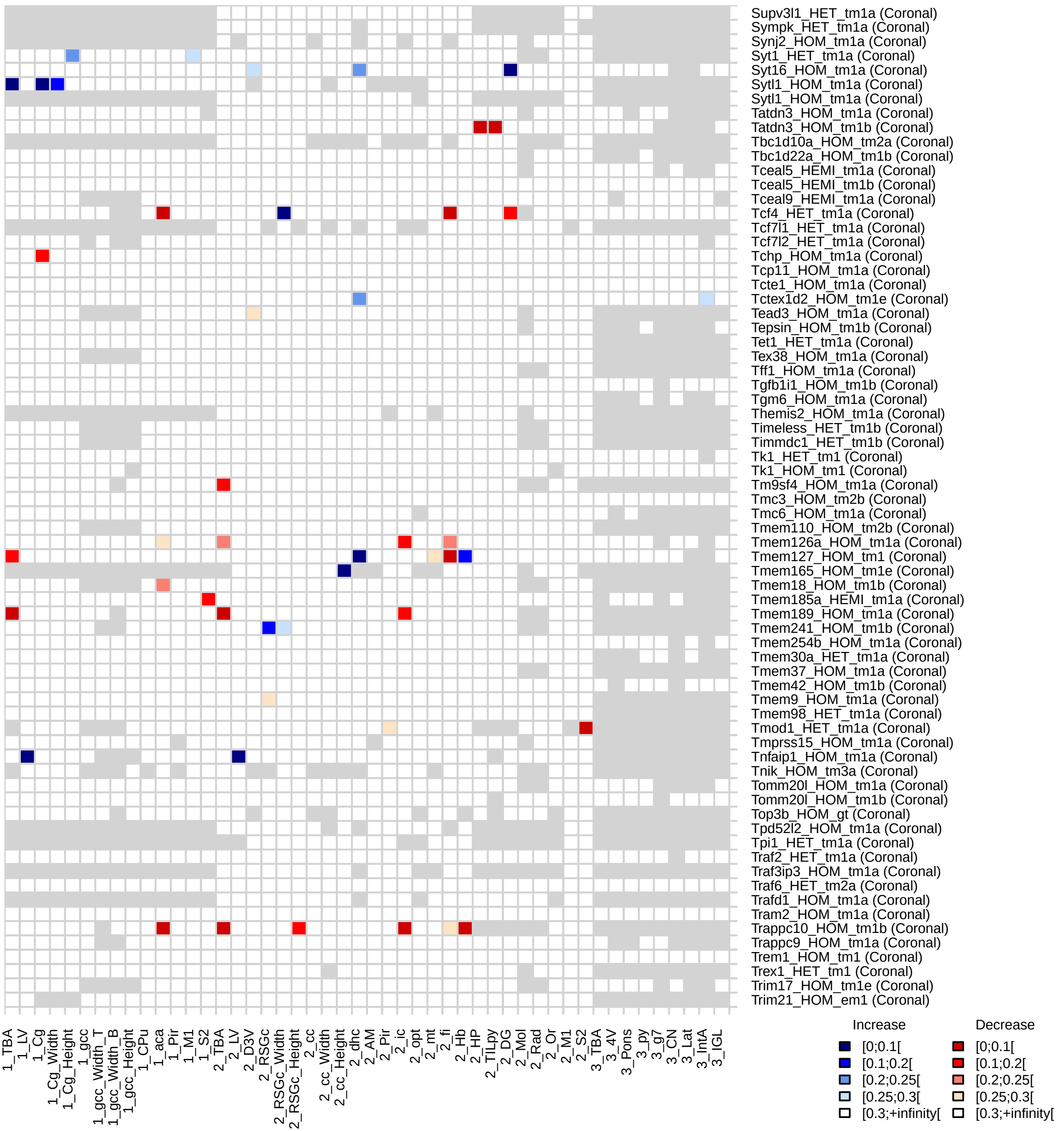

Supv3l1_HET_tm1a (Coronal)
Sympk_HET_tm1a (Coronal)
Synj2_HOM_tm1a (Coronal)
Syt1_HET_tm1a (Coronal)
Syt16_HOM_tm1a (Coronal)
Sytl1_HOM_tm1a (Coronal)
Sytl1_HOM_tm1a (Coronal)
Tatdn3_HOM_tm1a (Coronal)
Tatdn3_HOM_tm1b (Coronal)
Tbc1d10a_HOM_tm2a (Coronal)
Tbc1d22a_HOM_tm1b (Coronal)
Tceal5_HEMI_tm1a (Coronal)
Tceal5_HEMI_tm1b (Coronal)
Tceal9_HEMI_tm1a (Coronal)
Tcf4_HET_tm1a (Coronal)
Tcf7l1_HET_tm1a (Coronal)
Tcf7l2_HET_tm1a (Coronal)
Tchp_HOM_tm1a (Coronal)
Tcp11_HOM_tm1a (Coronal)
Tcte1_HOM_tm1a (Coronal)
Tctex1d2_HOM_tm1e (Coronal)
Tead3_HOM_tm1a (Coronal)
Tepsin_HOM_tm1b (Coronal)
Tet1_HET_tm1a (Coronal)
Tex38_HOM_tm1a (Coronal)
Tff1_HOM_tm1a (Coronal)
Tgfb1i1_HOM_tm1b (Coronal)
Tgm6_HOM_tm1a (Coronal)
Themis2_HOM_tm1a (Coronal)
Timeless_HET_tm1b (Coronal)
Timmdc1_HET_tm1b (Coronal)
Tk1_HET_tm1 (Coronal)
Tk1_HOM_tm1 (Coronal)
Tm9sf4_HOM_tm1a (Coronal)
Tmc3_HOM_tm2b (Coronal)
Tmc6_HOM_tm1a (Coronal)
Tmem110_HOM_tm2b (Coronal)
Tmem126a_HOM_tm1a (Coronal)
Tmem127_HOM_tm1 (Coronal)
Tmem165_HOM_tm1e (Coronal)
Tmem18_HOM_tm1b (Coronal)
Tmem185a_HEMI_tm1a (Coronal)
Tmem189_HOM_tm1a (Coronal)
Tmem241_HOM_tm1b (Coronal)
Tmem254b_HOM_tm1a (Coronal)
Tmem30a_HET_tm1a (Coronal)
Tmem37_HOM_tm1a (Coronal)
Tmem42_HOM_tm1b (Coronal)
Tmem9_HOM_tm1a (Coronal)
Tmem98_HET_tm1a (Coronal)
Tmod1_HET_tm1a (Coronal)
Tmprss15_HOM_tm1a (Coronal)
Tnfaip1_HOM_tm1a (Coronal)
Tnik_HOM_tm3a (Coronal)
Tomm20l_HOM_tm1a (Coronal)
Tomm20l_HOM_tm1b (Coronal)
Top3b_HOM_gt (Coronal)
Tpd52l2_HOM_tm1a (Coronal)
Tpi1_HET_tm1a (Coronal)
Traf2_HET_tm1a (Coronal)
Traf3ip3_HOM_tm1a (Coronal)
Traf6_HET_tm2a (Coronal)
Trafd1_HOM_tm1a (Coronal)
Tram2_HOM_tm1a (Coronal)
Trappc10_HOM_tm1b (Coronal)
Trappc9_HOM_tm1a (Coronal)
Trem1_HOM_tm1 (Coronal)
Trex1_HET_tm1 (Coronal)
Trim17_HOM_tm1e (Coronal)
Trim21_HOM_em1 (Coronal)
1_TBA
1_LV
1_Cg
1_Cg_Width
1_Cg_Height
1_gcc
1_gcc_Width_T
1_gcc_Width_B
1_gcc_Height
1_CPu
1_aca
1_Pir
1_M1
1_S2
2_TBA
2_LV
2_D3V
2_RSGc
2_RSGc_Width
2_RSGc_Height
2_cc
2_cc_Width
2_cc_Height
2_dhc
2_AM
2_Pir
2_ic
2_opt
2_mt
2_fi
2_Hb
2_HP
2_TILpy
2_DG
2_Mol
2_Rad
2_Or
2_M1
2_S2
3_TBA
3_4V
3_Pons
3_py
3_g7
3_CN
3_Lat
3_IntA
3_IGL
Increase
[0;0.1[
[0.1;0.2[
[0.2;0.25[
[0.25;0.3[
[0.3;+infinity[
Decrease
[0;0.1[
[0.1;0.2[
[0.2;0.25[
[0.25;0.3[
[0.3;+infinity[

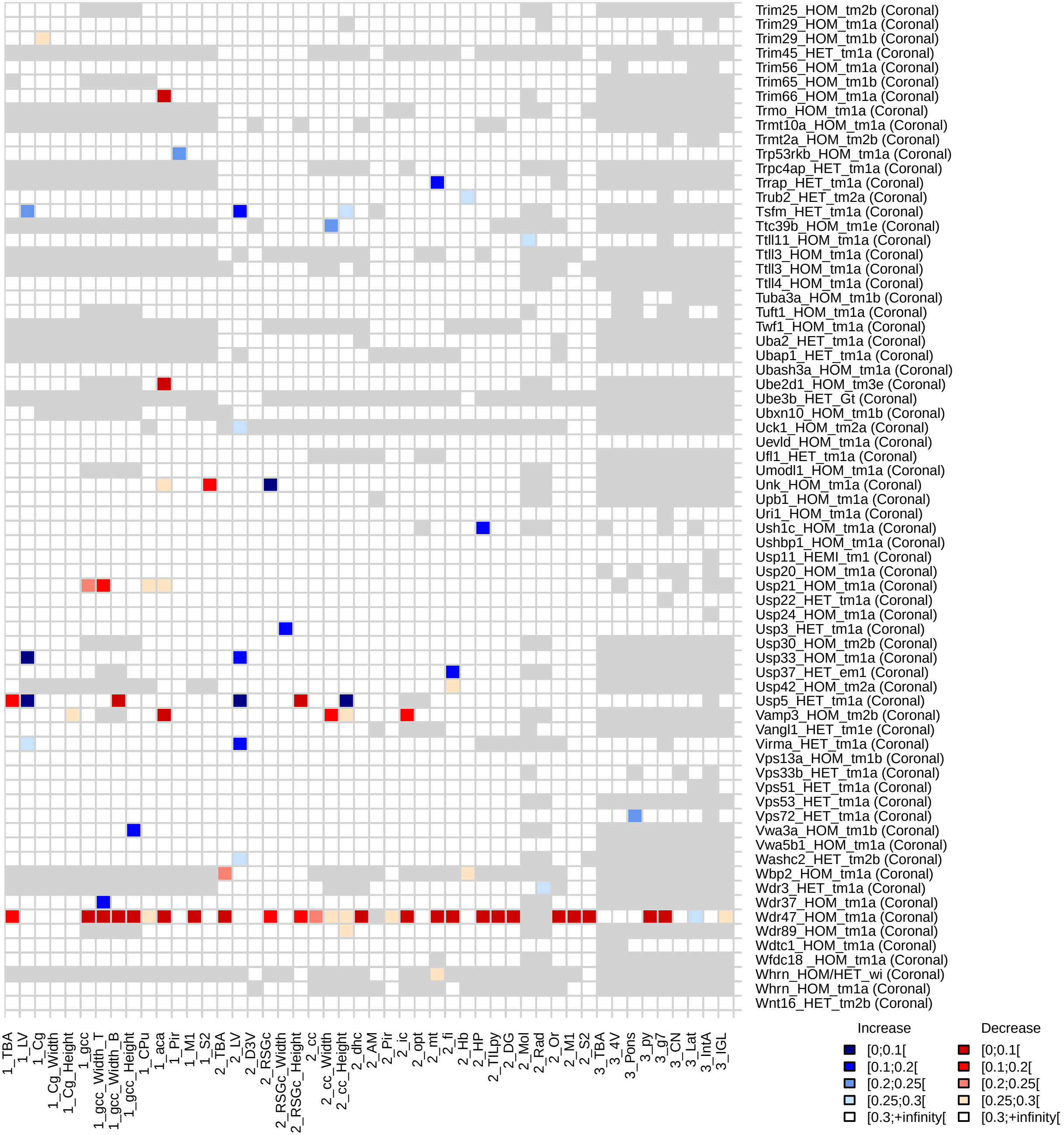
Trim25_HOM_tm2b (Coronal)
Trim29_HOM_tm1a (Coronal)
Trim29_HOM_tm1b (Coronal)
Trim45_HET_tm1a (Coronal)
Trim56_HOM_tm1a (Coronal)
Trim65_HOM_tm1b (Coronal)
Trim66_HOM_tm1a (Coronal)
Trmo_HOM_tm1a (Coronal)
Trmt10a_HOM_tm1a (Coronal)
Trmt2a_HOM_tm2b (Coronal)
Trp53rkb_HOM_tm1a (Coronal)
Trpc4ap_HET_tm1a (Coronal)
Trrap_HET_tm1a (Coronal)
Trub2_HET_tm2a (Coronal)
Tsfm_HET_tm1a (Coronal)
Ttc39b_HOM_tm1e (Coronal)
Ttll11_HOM_tm1a (Coronal)
Ttll3_HOM_tm1a (Coronal)
Ttll3_HOM_tm1a (Coronal)
Ttll4_HOM_tm1a (Coronal)
Tuba3a_HOM_tm1b (Coronal)
Tuft1_HOM_tm1a (Coronal)
Twf1_HOM_tm1a (Coronal)
Uba2_HET_tm1a (Coronal)
Ubap1_HET_tm1a (Coronal)
Ubash3a_HOM_tm1a (Coronal)
Ube2d1_HOM_tm3e (Coronal)
Ube3b_HET_Gt (Coronal)
Ubxn10_HOM_tm1b (Coronal)
Uck1_HOM_tm2a (Coronal)
Uevld_HOM_tm1a (Coronal)
Ufl1_HET_tm1a (Coronal)
Umodl1_HOM_tm1a (Coronal)
Unk_HOM_tm1a (Coronal)
Upb1_HOM_tm1a (Coronal)
Uri1_HOM_tm1a (Coronal)
Ush1c_HOM_tm1a (Coronal)
Ushbp1_HOM_tm1a (Coronal)
Usp11_HEMI_tm1 (Coronal)
Usp20_HOM_tm1a (Coronal)
Usp21_HOM_tm1a (Coronal)
Usp22_HET_tm1a (Coronal)
Usp24_HOM_tm1a (Coronal)
Usp3_HET_tm1a (Coronal)
Usp30_HOM_tm2b (Coronal)
Usp33_HOM_tm1a (Coronal)
Usp37_HET_em1 (Coronal)
Usp42_HOM_tm2a (Coronal)
Usp5_HET_tm1a (Coronal)
Vamp3_HOM_tm2b (Coronal)
Vangl1_HET_tm1e (Coronal)
Virma_HET_tm1a (Coronal)
Vps13a_HOM_tm1b (Coronal)
Vps33b_HET_tm1a (Coronal)
Vps51_HET_tm1a (Coronal)
Vps53_HET_tm1a (Coronal)
Vps72_HET_tm1a (Coronal)
Vwa3a_HOM_tm1b (Coronal)
Vwa5b1_HOM_tm1a (Coronal)
Washc2_HET_tm2b (Coronal)
Wbp2_HOM_tm1a (Coronal)
Wdr3_HET_tm1a (Coronal)
Wdr37_HOM_tm1a (Coronal)
Wdr47_HOM_tm1a (Coronal)
Wdr89_HOM_tm1a (Coronal)
Wdtc1_HOM_tm1a (Coronal)
Wfdc18_HOM_tm1a (Coronal)
Whrn_HOM/HET_wi (Coronal)
Whrn_HOM_tm1a (Coronal)
Wnt16_HET_tm2b (Coronal)
1_TBA
1_LV
1_Cg
1_Cg_Width
1_Cg_Height
1_gcc
1_gcc_Width_T
1_gcc_Width_B
1_gcc_Height
1_CPu
1_aca
1_Pir
1_M1
1_S2
2_TBA
2_LV
2_D3V
2_RSGc
2_RSGc_Width
2_RSGc_Height
2_cc
2_cc_Width
2_cc_Height
2_dhc
2_AM
2_Pir
2_ic
2_opt
2_mt
2_fi
2_Hb
2_HP
2_TILpy
2_DG
2_Mol
2_Rad
2_Or
2_M1
2_S2
3_TBA
3_4V
3_Pons
3_py
3_g7
3_CN
3_Lat
3_IntA
3_IGL
Increase
[0;0.1[
[0.1;0.2[
[0.2;0.25[
[0.25;0.3[
[0.3;+infinity[
Decrease
[0;0.1[
[0.1;0.2[
[0.2;0.25[
[0.25;0.3[
[0.3;+infinity[

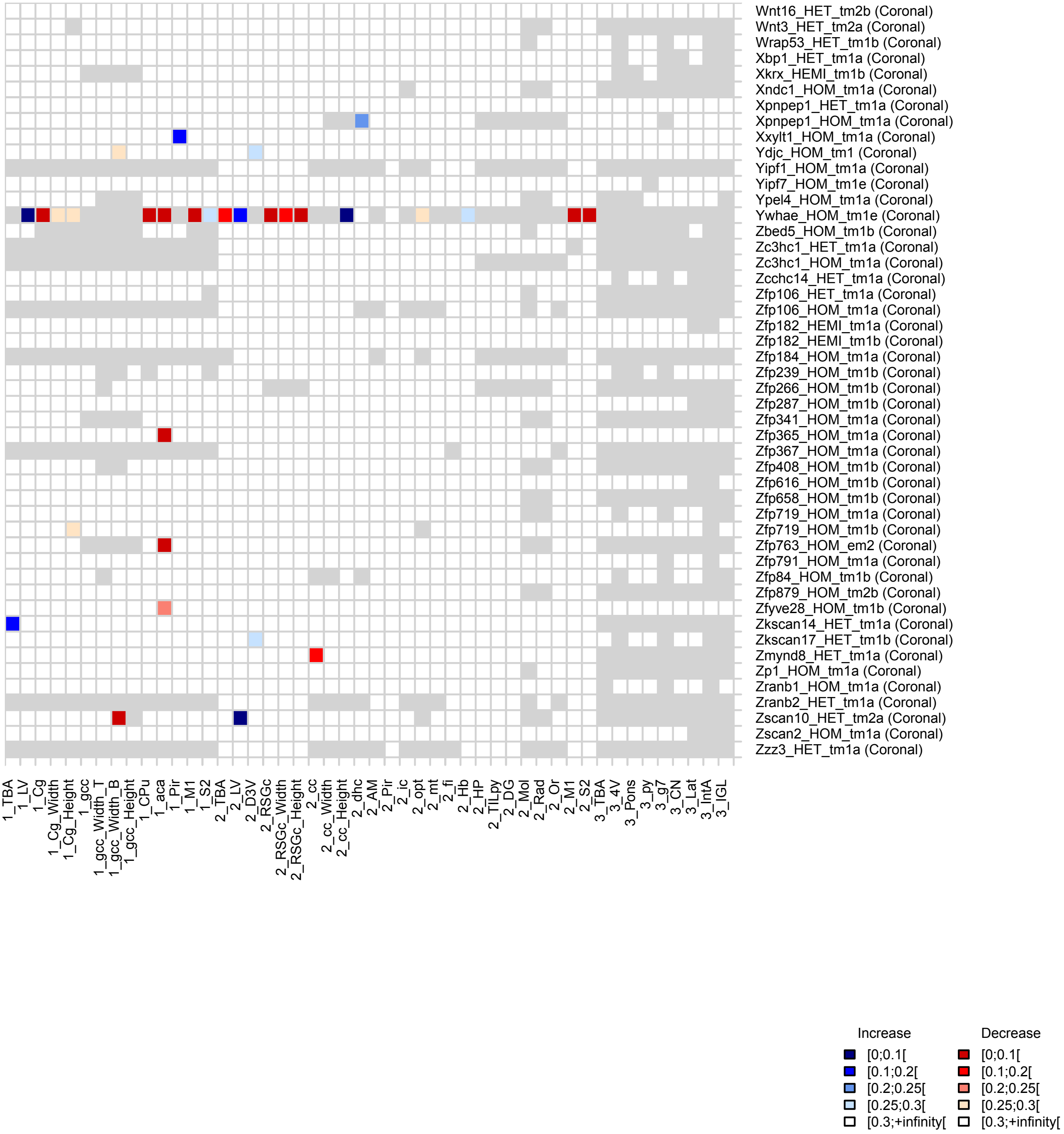

Wnt16_HET_tm2b (Coronal)
Wnt3_HET_tm2a (Coronal)
Wrap53_HET_tm1b (Coronal)
Xbp1_HET_tm1a (Coronal)
Xkrx_HEMI_tm1b (Coronal)
Xndc1_HOM_tm1a (Coronal)
Xpnpep1_HET_tm1a (Coronal)
Xpnpep1_HOM_tm1a (Coronal)
Xxylt1_HOM_tm1a (Coronal)
Ydjc_HOM_tm1 (Coronal)
Yipf1_HOM_tm1a (Coronal)
Yipf7_HOM_tm1e (Coronal)
Ypel4_HOM_tm1a (Coronal)
Ywhae_HOM_tm1e (Coronal)
Zbed5_HOM_tm1b (Coronal)
Zc3hc1_HET_tm1a (Coronal)
Zc3hc1_HOM_tm1a (Coronal)
Zcchc14_HET_tm1a (Coronal)
Zfp106_HET_tm1a (Coronal)
Zfp106_HOM_tm1a (Coronal)
Zfp182_HEMI_tm1a (Coronal)
Zfp182_HEMI_tm1b (Coronal)
Zfp184_HOM_tm1a (Coronal)
Zfp239_HOM_tm1b (Coronal)
Zfp266_HOM_tm1b (Coronal)
Zfp287_HOM_tm1b (Coronal)
Zfp341_HOM_tm1a (Coronal)
Zfp365_HOM_tm1a (Coronal)
Zfp367_HOM_tm1a (Coronal)
Zfp408_HOM_tm1b (Coronal)
Zfp616_HOM_tm1b (Coronal)
Zfp658_HOM_tm1b (Coronal)
Zfp719_HOM_tm1a (Coronal)
Zfp719_HOM_tm1b (Coronal)
Zfp763_HOM_em2 (Coronal)
Zfp791_HOM_tm1a (Coronal)
Zfp84_HOM_tm1b (Coronal)
Zfp879_HOM_tm2b (Coronal)
Zfyve28_HOM_tm1b (Coronal)
Zkscan14_HET_tm1a (Coronal)
Zkscan17_HET_tm1b (Coronal)
Zmynd8_HET_tm1a (Coronal)
Zp1_HOM_tm1a (Coronal)
Zranb1_HOM_tm1a (Coronal)
Zranb2_HET_tm1a (Coronal)
Zscan10_HET_tm2a (Coronal)
Zscan2_HOM_tm1a (Coronal)
Zzz3_HET_tm1a (Coronal)
1_TBA
1_LV
1_Cg
1_Cg_Width
1_Cg_Height
1_gcc
1_gcc_Width_T
1_gcc_Width_B
1_gcc_Height
1_CPu
1_aca
1_Pir
1_M1
1_S2
2_TBA
2_LV
2_D3V
2_RSGc
2_RSGc_Width
2_RSGc_Height
2_cc
2_cc_Width
2_cc_Height
2_dhc
2_AM
2_Pir
2_ic
2_opt
2_mt
2_fi
2_Hb
2_HP
2_TILpy
2_DG
2_Mol
2_Rad
2_Or
2_M1
2_S2
3_TBA
3_4V
3_Pons
3_py
3_g7
3_CN
3_Lat
3_IntA
3_IGL
Increase
[0;0.1[
[0.1;0.2[
[0.2;0.25[
[0.25;0.3[
[0.3;+infinity[
Decrease
[0;0.1[
[0.1;0.2[
[0.2;0.25[
[0.25;0.3[
[0.3;+infinity[

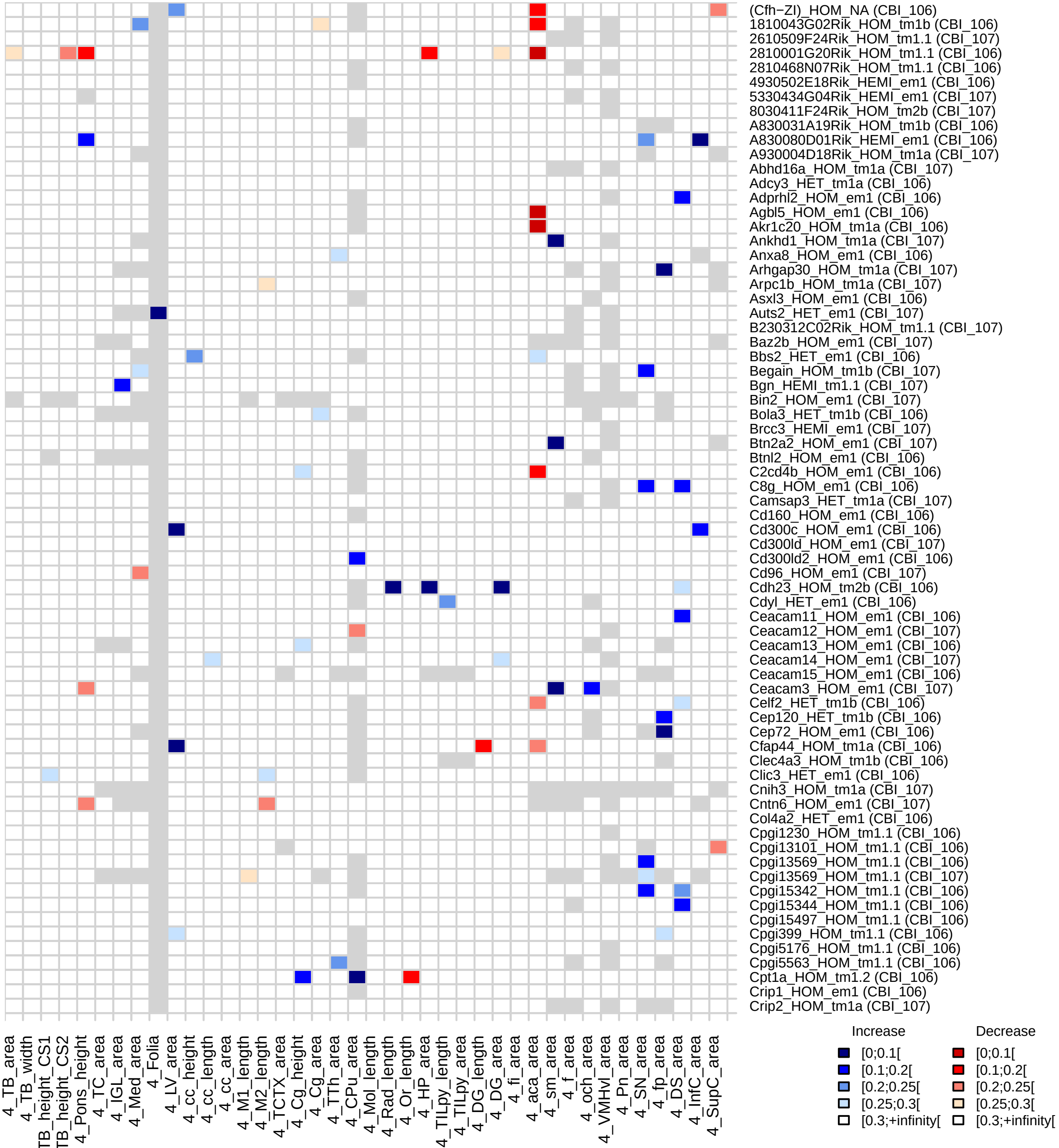

(Cfh−Zl)_HOM_NA (CBI_106)
1810043G02Rik_HOM_tm1b (CBI_106)
2610509F24Rik_HOM_tm1.1 (CBI_107)
2810001G20Rik_HOM_tm1.1 (CBI_106)
2810468N07Rik_HOM_tm1.1 (CBI_106)
4930502E18Rik_HEMI_em1 (CBI_106)
5330434G04Rik_HEMI_em1 (CBI_107)
8030411F24Rik_HOM_tm2b (CBI_107)
A830031A19Rik_HOM_tm1b (CBI_106)
A830080D01Rik_HEMI_em1 (CBI_106)
A930004D18Rik_HOM_tm1a (CBI_107)
Abhd16a_HOM_tm1a (CBI_107)
Adcy3_HET_tm1a (CBI_106)
Adprhl2_HOM_em1 (CBI_106)
Agbl5_HOM_em1 (CBI_106)
Akr1c20_HOM_tm1a (CBI_106)
Ankhd1_HOM_tm1a (CBI_107)
Anxa8_HOM_em1 (CBI_106)
Arhgap30_HOM_tm1a (CBI_107)
Arpc1b_HOM_tm1a (CBI_107)
Asxl3_HOM_em1 (CBI_106)
Auts2_HET_em1 (CBI_107)
B230312C02Rik_HOM_tm1.1 (CBI_107)
Baz2b_HOM_em1 (CBI_107)
Bbs2_HET_em1 (CBI_106)
Begain_HOM_tm1b (CBI_107)
Bgn_HEMI_tm1.1 (CBI_107)
Bin2_HOM_em1 (CBI_107)
Bola3_HET_tm1b (CBI_106)
Brcc3_HEMI_em1 (CBI_107)
Btn2a2_HOM_em1 (CBI_107)
Btnl2_HOM_em1 (CBI_106)
C2cd4b_HOM_em1 (CBI_106)
C8g_HOM_em1 (CBI_106)
Camsap3_HET_tm1a (CBI_107)
Cd160_HOM_em1 (CBI_106)
Cd300c_HOM_em1 (CBI_106)
Cd300ld_HOM_em1 (CBI_107)
Cd300ld2_HOM_em1 (CBI_106)
Cd96_HOM_em1 (CBI_107)
Cdh23_HOM_tm2b (CBI_106)
Cdyl_HET_em1 (CBI_106)
Ceacam11_HOM_em1 (CBI_106)
Ceacam12_HOM_em1 (CBI_107)
Ceacam13_HOM_em1 (CBI_106)
Ceacam14_HOM_em1 (CBI_107)
Ceacam15_HOM_em1 (CBI_106)
Ceacam3_HOM_em1 (CBI_107)
Celf2_HET_tm1b (CBI_106)
Cep120_HET_tm1b (CBI_106)
Cep72_HOM_em1 (CBI_106)
Cfap44_HOM_tm1a (CBI_106)
Clec4a3_HOM_tm1b (CBI_106)
Clic3_HET_em1 (CBI_106)
Cnih3_HOM_tm1a (CBI_107)
Cntn6_HOM_em1 (CBI_107)
Col4a2_HET_em1 (CBI_106)
Cpgi1230_HOM_tm1.1 (CBI_106)
Cpgi13101_HOM_tm1.1 (CBI_106)
Cpgi13569_HOM_tm1.1 (CBI_106)
Cpgi13569_HOM_tm1.1 (CBI_107)
Cpgi15342_HOM_tm1.1 (CBI_106)
Cpgi15344_HOM_tm1.1 (CBI_106)
Cpgi15497_HOM_tm1.1 (CBI_106)
Cpgi399_HOM_tm1.1 (CBI_106)
Cpgi5176_HOM_tm1.1 (CBI_106)
Cpgi5563_HOM_tm1.1 (CBI_106)
Cpt1a_HOM_tm1.2 (CBI_106)
Crip1_HOM_em1 (CBI_106)
Crip2_HOM_tm1a (CBI_107)
4_TB_area
4_TB_width
TB_height_CS1
TB_height_CS2
4_Pons_height
4_TC_area
4_IGL_area
4_Med_area
4_Folia
4_LV_area
4_cc_height
4_cc_length
4_cc_area
4_M1_length
4_M2_length
4_TCTX_area
4_Cg_height
4_Cg_area
4_TTh_area
4_CPu_area
4_Mol_length
4_Rad_length
4_Or_length
4_HP_area
4_TILpy_length
4_TILpy_area
4_DG_length
4_DG_area
4_fi_area
4_aca_area
4_sm_area
4_f_area
4_och_area
4_VMHvl_area
4_Pn_area
4_SN_area
4_fp_area
4_DS_area
4_InfC_area
4_SupC_area
Increase
[0;0.1[
[0.1;0.2[
[0.2;0.25[
[0.25;0.3[
[0.3;+infinity[
Decrease
[0;0.1[
[0.1;0.2[
[0.2;0.25[
[0.25;0.3[
[0.3;+infinity[

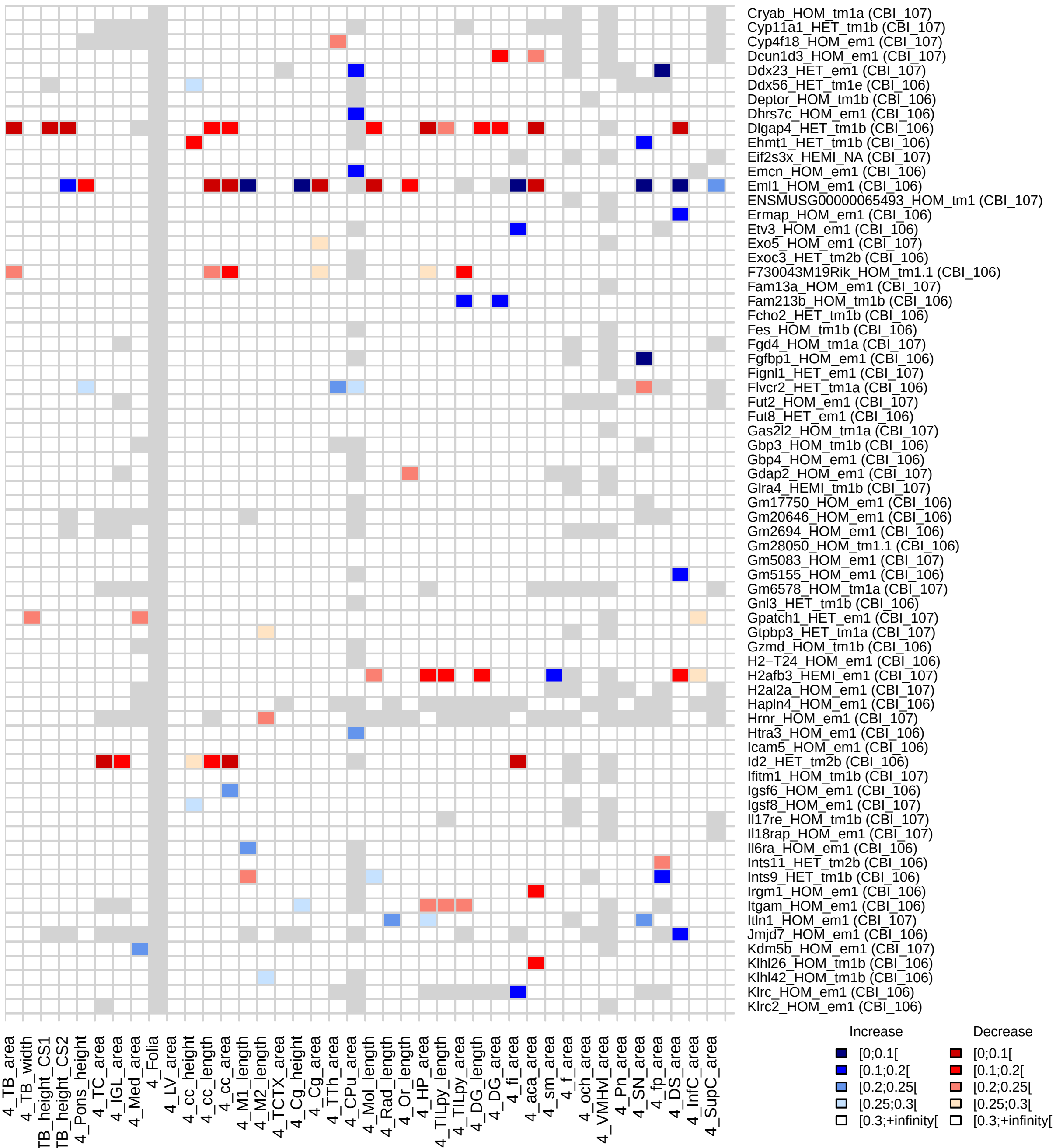

Cryab_HOM_tm1a (CBI_107)
Cyp11a1_HET_tm1b (CBI_107)
Cyp4f18_HOM_em1 (CBI_107)
Dcun1d3_HOM_em1 (CBI_107)
Ddx23_HET_em1 (CBI_107)
Ddx56_HET_tm1e (CBI_106)
Deptor_HOM_tm1b (CBI_106)
Dhrs7c_HOM_em1 (CBI_106)
Dlgap4_HET_tm1b (CBI_106)
Ehmt1_HET_tm1b (CBI_106)
Eif2s3x_HEMI_NA (CBI_107)
Emcn_HOM_em1 (CBI_106)
Eml1_HOM_em1 (CBI_106)
ENSMUSG00000065493_HOM_tm1 (CBI_107)
Ermap_HOM_em1 (CBI_106)
Etv3_HOM_em1 (CBI_106)
Exo5_HOM_em1 (CBI_107)
Exoc3_HET_tm2b (CBI_106)
F730043M19Rik_HOM_tm1.1 (CBI_106)
Fam13a_HOM_em1 (CBI_107)
Fam213b_HOM_tm1b (CBI_106)
Fcho2_HET_tm1b (CBI_106)
Fes_HOM_tm1b (CBI_106)
Fgd4_HOM_tm1a (CBI_107)
Fgfbp1_HOM_em1 (CBI_106)
Fignl1_HET_em1 (CBI_107)
Flvcr2_HET_tm1a (CBI_106)
Fut2_HOM_em1 (CBI_107)
Fut8_HET_em1 (CBI_106)
Gas2l2_HOM_tm1a (CBI_107)
Gbp3_HOM_tm1b (CBI_106)
Gbp4_HOM_em1 (CBI_106)
Gdap2_HOM_em1 (CBI_107)
Glra4_HEMI_tm1b (CBI_107)
Gm17750_HOM_em1 (CBI_106)
Gm20646_HOM_em1 (CBI_106)
Gm2694_HOM_em1 (CBI_106)
Gm28050_HOM_tm1.1 (CBI_106)
Gm5083_HOM_em1 (CBI_107)
Gm5155_HOM_em1 (CBI_106)
Gm6578_HOM_tm1a (CBI_107)
Gnl3_HET_tm1b (CBI_106)
Gpatch1_HET_em1 (CBI_107)
Gtpbp3_HET_tm1a (CBI_107)
Gzmd_HOM_tm1b (CBI_106)
H2−T24_HOM_em1 (CBI_106)
H2afb3_HEMI_em1 (CBI_107)
H2al2a_HOM_em1 (CBI_107)
Hapln4_HOM_em1 (CBI_106)
Hrnr_HOM_em1 (CBI_107)
Htra3_HOM_em1 (CBI_106)
Icam5_HOM_em1 (CBI_106)
Id2_HET_tm2b (CBI_106)
Ifitm1_HOM_tm1b (CBI_107)
Igsf6_HOM_em1 (CBI_106)
Igsf8_HOM_em1 (CBI_107)
Il17re_HOM_tm1b (CBI_107)
Il18rap_HOM_em1 (CBI_107)
Il6ra_HOM_em1 (CBI_106)
Ints11_HET_tm2b (CBI_106)
Ints9_HET_tm1b (CBI_106)
Irgm1_HOM_em1 (CBI_106)
Itgam_HOM_em1 (CBI_106)
Itln1_HOM_em1 (CBI_107)
Jmjd7_HOM_em1 (CBI_106)
Kdm5b_HOM_em1 (CBI_107)
Klhl26_HOM_tm1b (CBI_106)
Klhl42_HOM_tm1b (CBI_106)
Klrc_HOM_em1 (CBI_106)
Klrc2_HOM_em1 (CBI_106)
4_TB_area
4_TB_width
TB_height_CS1
TB_height_CS2
4_Pons_height
4_TC_area
4_IGL_area
4_Med_area
4_Folia
4_LV_area
4_cc_height
4_cc_length
4_cc_area
4_M1_length
4_M2_length
4_TCTX_area
4_Cg_height
4_Cg_area
4_TTh_area
4_CPu_area
4_Mol_length
4_Rad_length
4_Or_length
4_HP_area
4_TILpy_length
4_TILpy_area
4_DG_length
4_DG_area
4_fi_area
4_aca_area
4_sm_area
4_f_area
4_och_area
4_VMHvl_area
4_Pn_area
4_SN_area
4_fp_area
4_DS_area
4_InfC_area
4_SupC_area
Increase
[0;0.1[
[0.1;0.2[
[0.2;0.25[
[0.25;0.3[
[0.3;+infinity[
Decrease
[0;0.1[
[0.1;0.2[
[0.2;0.25[
[0.25;0.3[
[0.3;+infinity[

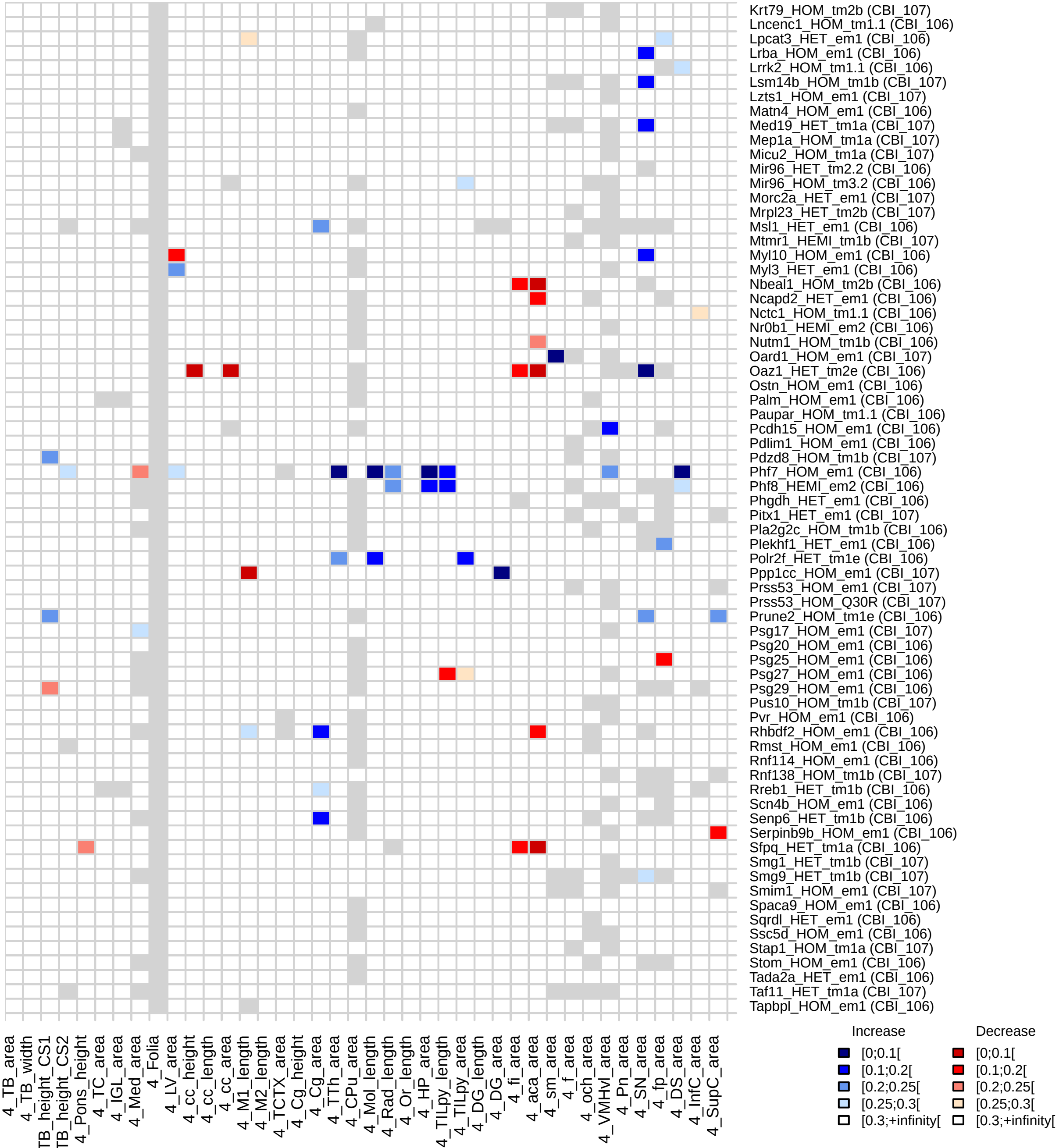
Krt79_HOM_tm2b (CBI_107)
Lncenc1_HOM_tm1.1 (CBI_106)
Lpcat3_HET_em1 (CBI_106)
Lrba_HOM_em1 (CBI_106)
Lrrk2_HOM_tm1.1 (CBI_106)
Lsm14b_HOM_tm1b (CBI_107)
Lzts1_HOM_em1 (CBI_107)
Matn4_HOM_em1 (CBI_106)
Med19_HET_tm1a (CBI_107)
Mep1a_HOM_tm1a (CBI_107)
Micu2_HOM_tm1a (CBI_107)
Mir96_HET_tm2.2 (CBI_106)
Mir96_HOM_tm3.2 (CBI_106)
Morc2a_HET_em1 (CBI_107)
Mrpl23_HET_tm2b (CBI_107)
Msl1_HET_em1 (CBI_106)
Mtmr1_HEMI_tm1b (CBI_107)
Myl10_HOM_em1 (CBI_106)
Myl3_HET_em1 (CBI_106)
Nbeal1_HOM_tm2b (CBI_106)
Ncapd2_HET_em1 (CBI_106)
Nctc1_HOM_tm1.1 (CBI_106)
Nr0b1_HEMI_em2 (CBI_106)
Nutm1_HOM_tm1b (CBI_106)
Oard1_HOM_em1 (CBI_107)
Oaz1_HET_tm2e (CBI_106)
Ostn_HOM_em1 (CBI_106)
Palm_HOM_em1 (CBI_106)
Paupar_HOM_tm1.1 (CBI_106)
Pcdh15_HOM_em1 (CBI_106)
Pdlim1_HOM_em1 (CBI_106)
Pdzd8_HOM_tm1b (CBI_107)
Phf7_HOM_em1 (CBI_106)
Phf8_HEMI_em2 (CBI_106)
Phgdh_HET_em1 (CBI_106)
Pitx1_HET_em1 (CBI_107)
Pla2g2c_HOM_tm1b (CBI_106)
Plekhf1_HET_em1 (CBI_106)
Polr2f_HET_tm1e (CBI_106)
Ppp1cc_HOM_em1 (CBI_107)
Prss53_HOM_em1 (CBI_107)
Prss53_HOM_Q30R (CBI_107)
Prune2_HOM_tm1e (CBI_106)
Psg17_HOM_em1 (CBI_107)
Psg20_HOM_em1 (CBI_106)
Psg25_HOM_em1 (CBI_106)
Psg27_HOM_em1 (CBI_106)
Psg29_HOM_em1 (CBI_106)
Pus10_HOM_tm1b (CBI_107)
Pvr_HOM_em1 (CBI_106)
Rhbdf2_HOM_em1 (CBI_106)
Rmst_HOM_em1 (CBI_106)
Rnf114_HOM_em1 (CBI_106)
Rnf138_HOM_tm1b (CBI_107)
Rreb1_HET_tm1b (CBI_106)
Scn4b_HOM_em1 (CBI_106)
Senp6_HET_tm1b (CBI_106)
Serpinb9b_HOM_em1 (CBI_106)
Sfpq_HET_tm1a (CBI_106)
Smg1_HET_tm1b (CBI_107)
Smg9_HET_tm1b (CBI_107)
Smim1_HOM_em1 (CBI_107)
Spaca9_HOM_em1 (CBI_106)
Sqrdl_HET_em1 (CBI_106)
Ssc5d_HOM_em1 (CBI_106)
Stap1_HOM_tm1a (CBI_107)
Stom_HOM_em1 (CBI_106)
Tada2a_HET_em1 (CBI_106)
Taf11_HET_tm1a (CBI_107)
Tapbpl_HOM_em1 (CBI_106)
4_TB_area
4_TB_width
TB_height_CS1
TB_height_CS2
4_Pons_height
4_TC_area
4_IGL_area
4_Med_area
4_Folia
4_LV_area
4_cc_height
4_cc_length
4_cc_area
4_M1_length
4_M2_length
4_TCTX_area
4_Cg_height
4_Cg_area
4_TTh_area
4_CPu_area
4_Mol_length
4_Rad_length
4_Or_length
4_HP_area
4_TILpy_length
4_TILpy_area
4_DG_length
4_DG_area
4_fi_area
4_aca_area
4_sm_area
4_f_area
4_och_area
4_VMHvl_area
4_Pn_area
4_SN_area
4_fp_area
4_DS_area
4_InfC_area
4_SupC_area
Increase
[0;0.1[
[0.1;0.2[
[0.2;0.25[
[0.25;0.3[
[0.3;+infinity[
Decrease
[0;0.1[
[0.1;0.2[
[0.2;0.25[
[0.25;0.3[
[0.3;+infinity[

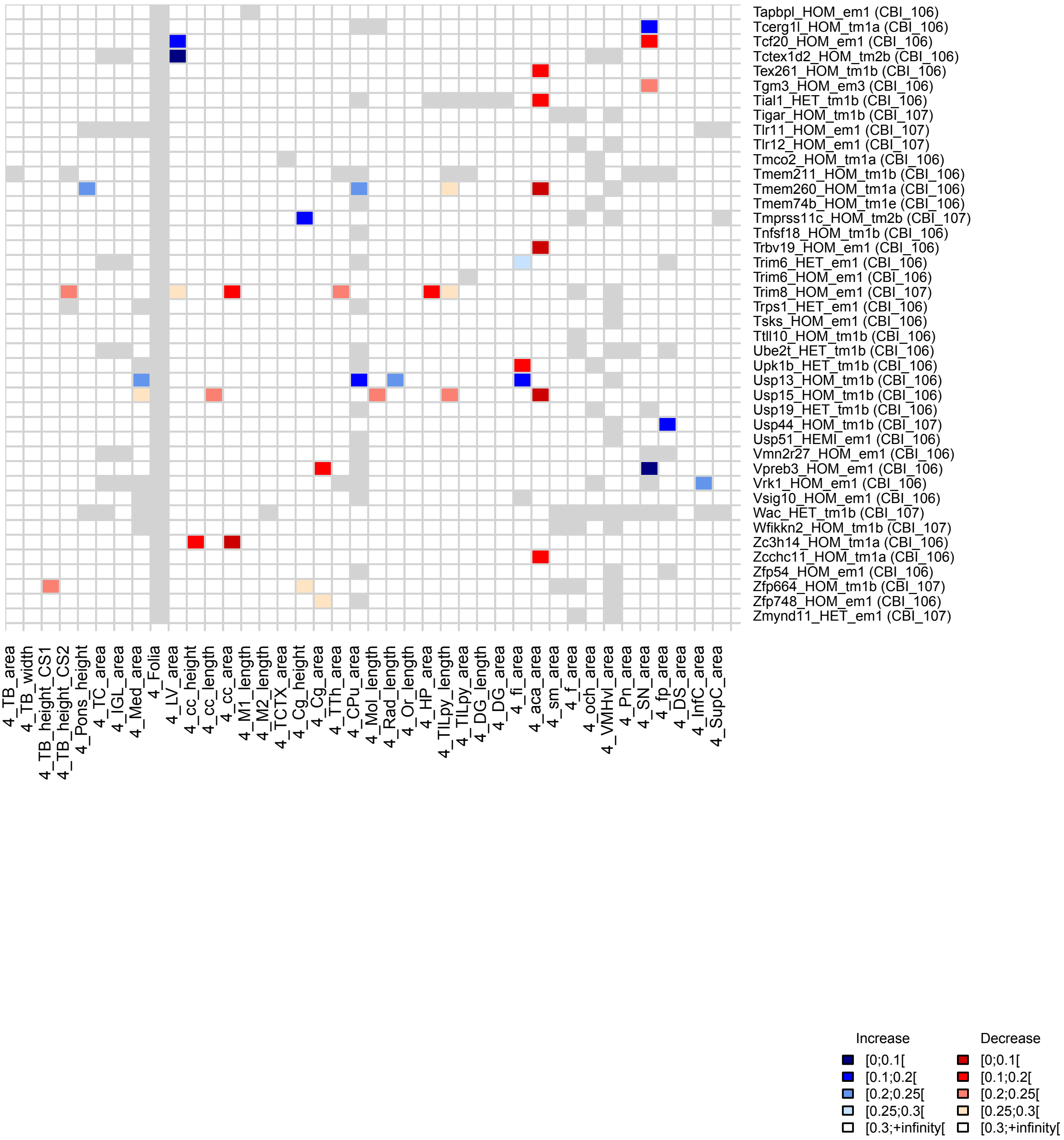

Tapbpl_HOM_em1 (CBI_106)
Tcerg1l_HOM_tm1a (CBI_106)
Tcf20_HOM_em1 (CBI_106)
Tctex1d2_HOM_tm2b (CBI_106)
Tex261_HOM_tm1b (CBI_106)
Tgm3_HOM_em3 (CBI_106)
Tial1_HET_tm1b (CBI_106)
Tigar_HOM_tm1b (CBI_107)
Tlr11_HOM_em1 (CBI_107)
Tlr12_HOM_em1 (CBI_107)
Tmco2_HOM_tm1a (CBI_106)
Tmem211_HOM_tm1b (CBI_106)
Tmem260_HOM_tm1a (CBI_106)
Tmem74b_HOM_tm1e (CBI_106)
Tmprss11c_HOM_tm2b (CBI_107)
Tnfsf18_HOM_tm1b (CBI_106)
Trbv19_HOM_em1 (CBI_106)
Trim6_HET_em1 (CBI_106)
Trim6_HOM_em1 (CBI_106)
Trim8_HOM_em1 (CBI_107)
Trps1_HET_em1 (CBI_106)
Tsks_HOM_em1 (CBI_106)
Ttll10_HOM_tm1b (CBI_106)
Ube2t_HET_tm1b (CBI_106)
Upk1b_HET_tm1b (CBI_106)
Usp13_HOM_tm1b (CBI_106)
Usp15_HOM_tm1b (CBI_106)
Usp19_HET_tm1b (CBI_106)
Usp44_HOM_tm1b (CBI_107)
Usp51_HEMI_em1 (CBI_106)
Vmn2r27_HOM_em1 (CBI_106)
Vpreb3_HOM_em1 (CBI_106)
Vrk1_HOM_em1 (CBI_106)
Vsig10_HOM_em1 (CBI_106)
Wac_HET_tm1b (CBI_107)
Wfikkn2_HOM_tm1b (CBI_107)
Zc3h14_HOM_tm1a (CBI_106)
Zcchc11_HOM_tm1a (CBI_106)
Zfp54_HOM_em1 (CBI_106)
Zfp664_HOM_tm1b (CBI_107)
Zfp748_HOM_em1 (CBI_106)
Zmynd11_HET_em1 (CBI_107)
4_TB_area
4_TB_width
4_TB_height_CS1
4_TB_height_CS2
4_Pons_height
4_TC_area
4_IGL_area
4_Med_area
4_Folia
4_LV_area
4_cc_height
4_cc_length
4_cc_area
4_M1_length
4_M2_length
4_TCTX_area
4_Cg_height
4_Cg_area
4_TTh_area
4_CPu_area
4_Mol_length
4_Rad_length
4_Or_length
4_HP_area
4_TILpy_length
4_TILpy_area
4_DG_length
4_DG_area
4_fi_area
4_aca_area
4_sm_area
4_f_area
4_och_area
4_VMHvl_area
4_Pn_area
4_SN_area
4_fp_area
4_DS_area
4_InfC_area
4_SupC_area
Increase
[0;0.1[
[0.1;0.2[
[0.2;0.25[
[0.25;0.3[
[0.3;+infinity[
Decrease
[0;0.1[
[0.1;0.2[
[0.2;0.25[
[0.25;0.3[
[0.3;+infinity[
