# Supplementary material for: Large-scale neuroanatomical study uncovers 198 gene associations in mouse brain morphogenesis
Source: Nat Commun. 2019 Aug 1;10:3465. doi: 10.1038/s41467-019-11431-2 (PMC6671969; doi:10.1038/s41467-019-11431-2)
Supplement: Supplementary file 2 — Peer Review [file 41467_2019_11431_MOESM2_ESM.pdf]

Editorial Note: This manuscript has been previously reviewed at another journal that is not operating a transparent peer review scheme. This document only contains reviewer comments and rebuttal letters for versions considered at Nature Communications.
